# Targeted Chemical Monitoring and Integrated Preclinical Evaluation of Zishui Qinggan Yin in 4-NQO-Induced Esophageal Precancer: Histopathological and PI3K/AKT/mTOR-Associated Changes

**DOI:** 10.3390/life16091574

**Published:** 2026-09-21

**Authors:** Yueheng Ouyang, Jianxin Guo, Jianfeng Yuan, Zhongbing Wu, Jing Li

**Affiliations:** College of Integrated Chinese and Western Medicine, Hebei Medical University, Shijiazhuang 050017, China

**Keywords:** Zishui Qinggan Yin, esophageal precancer, 4-nitroquinoline-1-oxide, targeted UPLC-Q exactive/MS, digital pathology, AKT, PI3K/AKT/mTOR, drug affinity responsive target stability

## Abstract

Esophageal squamous cell carcinoma frequently develops through a prolonged precancerous phase, creating an opportunity for intervention. Zishui Qinggan Yin (ZSQGY) is a multi-component botanical preparation, but its effects in esophageal precancer and its formulation-level chemical profile remain incompletely defined. We monitored a predefined 40-analyte panel by UPLC-Q Exactive/MS in three independently extracted aliquots from the same formulation batch administered to mice. A 4-nitroquinoline-1-oxide model was then evaluated by consensus histopathology, mouse-grouped whole-slide digital pathology, network-based target prioritization, molecular docking, exploratory drug affinity responsive target stability (DARTS), Western blotting, qPCR, and serial-section immunohistochemistry. Signals corresponding to 38 predefined analytes were reported. ZSQGY treatment was associated with a lower-grade lesion pattern in the evaluated mouse-level specimens. Exploratory DARTS was analyzed in two independent *n* = 6 tissue-background datasets. In the original 4-NQO model-background cohort, ex vivo ZSQGY (100 μg/mL) increased the AKT1-immunoreactive relative-protection signal in five of six lysates (mean 2.04 ± 1.15). In an additional cohort from mice that had received ZSQGY in vivo, ex vivo ZSQGY produced mean relative-protection values of 1.21, 1.75, and 1.92 at 50, 100, and 200 μg/mL, respectively. The nominal exact *p* value for the 100 μg/mL versus vehicle contrast in the additional exploratory dataset was 0.0313; This exploratory result should be interpreted cautiously. Neither dataset establishes direct binding or in vivo target occupancy. PI3K p110α DARTS was technically inconclusive. Endpoint tissues showed lower AKT, mTOR, S6K1, and 4E-BP1 phosphorylation, together with PTEN-associated changes, reduced Ki-67, increased cleaved caspase-3 staining, and selected downstream transcript changes, including a non-significant downward trend in PARP1. These findings support a preclinical association between ZSQGY treatment, attenuation of esophageal precancer progression, and AKT-centered PI3K/AKT/mTOR pathway modulation. They do not establish direct binding, in vivo target occupancy, pathway necessity, active constituents, or causal mediation.

## 1. Introduction

Esophageal cancer remains a major cause of cancer mortality worldwide [1]. Esophageal squamous cell carcinoma (ESCC) predominates in high-risk regions of China, where graded squamous dysplasia and carcinoma in situ form a clinically actionable precancerous continuum [2]. Histologic grade is associated with subsequent malignant transformation risk [2]. More broadly, cancer interception seeks to intervene before invasive disease becomes established [3,4,5], and endoscopic treatment can be effective for selected early lesions [6]. However, invasive procedures and resource requirements motivate the evaluation of lower-intensity interventions during the precancerous window.

Progression toward ESCC involves genetic and epigenetic alterations affecting proliferation, survival, and metabolism [7,8]. PI3K/AKT/mTOR signaling is frequently dysregulated in ESCC and is biologically relevant to growth, protein synthesis, metabolic adaptation, and resistance to apoptosis [9,10,11,12,13,14,15]. Whole-slide computational pathology can complement expert review by providing reproducible spatial summaries of heterogeneous tissue, but performance estimates depend strongly on the unit of data partitioning and require validation beyond patch-level splits [16,17,18].

Zishui Qinggan Yin (ZSQGY) is an 11-component botanical formula historically described in traditional Chinese medicine. Here, it was studied as a defined multi-component preparation in an experimental model, rather than as evidence for a traditional syndrome classification. Its formulation-level chemical profile, effects on esophageal precancer progression, and relationship to pathway-associated tissue readouts have not been adequately characterized.

We therefore evaluated ZSQGY in a 4-nitroquinoline-1-oxide (4-NQO)-induced murine model of esophageal squamous precancer [19,20,21]. Targeted UPLC-Q Exactive/MS monitoring of three independently extracted aliquots characterized the predefined analyte panel in the formulation batch administered to mice. Consensus histopathology and mouse-grouped whole-slide digital pathology were combined with hypothesis-generating network analysis and docking. Exploratory DARTS was then used to assess whether an AKT1-immunoreactive band detected using an AKT1-directed antibody or PI3K p110α showed ligand-associated protease protection, followed by Western blotting, qPCR, and serial-section IHC to evaluate pathway-associated effects. We tested whether ZSQGY-associated attenuation of lesion progression was accompanied by an AKT-centered change in PI3K/AKT/mTOR signaling; the study was not designed to identify active constituents, prove direct binding, or establish a single-target causal mechanism.

## 2. Materials and Methods

### 2.1. ZSQGY Composition, Preparation, and Dose

ZSQGY was prepared from 11 commercially manufactured dispensing-granule products. The botanical sources, medicinal parts, and mouse-equivalent doses are provided in Supplementary Table S1 and listed in the main text below. The granules were purchased from Shixing Pharmacy (Shijiazhuang, China), and the supplied records named Guangdong Yifang Pharmaceutical Co., Ltd. (Guangzhou, China) and Jiangyin Tianjiang Pharmaceutical Co., Ltd. (Jiangyin, China) as manufacturers. However, the available records did not map each individual herb to one manufacturer and did not provide herb-specific lot numbers or extraction-equivalence specifications; these details were therefore not inferred. The formulation material submitted for LC-MS analysis and the material administered in the animal study originated from the same formulation batch; this batch was chemically characterized by three independent extractions. The component doses were converted using a body-surface-area factor of 9.1 (mouse-equivalent dose = corresponding human dose × 9.1). The resulting 11 converted component doses summed to 6.638 g/kg/day on the basis of actual dispensing-granule mass. For a 20 g mouse, this corresponded to 0.13276 g (132.76 mg) per day. At a reference administration volume of 0.1 mL/10 g, the corresponding solution concentration was 663.8 mg/mL. The dosing solution was prepared on each dosing day, held temporarily at 4 °C, rewarmed to 37 °C and mixed before administration, and used on the same day; the holding period did not exceed 24 h and the freeze–thaw count was zero. No formal long-term stability study was performed, so no long-term stability claim is made. From weeks 14 to 28, ZSQGY was administered once daily by oral gavage at 6.638 g/kg/day. To minimize gavage-associated mechanical injury, oral administration was performed by trained personnel using a stainless-steel gavage needle with a blunted tip (manufacturer and model not retained in the supplied records) (inner diameter: 0.9 mm, length: 3 cm). Mice were gently restrained during dosing, and the needle was advanced smoothly without force. The administration volume was adjusted to the dosing-day body weight at 0.1 mL/10 g, corresponding to approximately 0.18, 0.20, and 0.22 mL per mouse per day at 18, 20, and 22 g, respectively. These precautions reduce but do not eliminate the possibility of gavage-related mechanical injury; no dedicated gavage-injury score was prespecified.

For completeness, the 11-component formula and mouse-equivalent daily doses were: Paeoniae Radix Alba (0.430 g/kg), Bupleuri Radix (0.250 g/kg), Moutan Cortex (0.334 g/kg), Angelicae Sinensis Radix (1.334 g/kg), Poria (0.080 g/kg), Gardeniae Fructus (0.100 g/kg), Dioscoreae Rhizoma (0.750 g/kg), Corni Fructus (0.300 g/kg), Rehmanniae Radix Praeparata (2.310 g/kg), Ziziphi Spinosae Semen (0.500 g/kg), and Alismatis Rhizoma (0.250 g/kg); the total was 6.638 g/kg/day.

### 2.2. Targeted UPLC-Q Exactive/MS Monitoring

A predefined panel of 40 flavonoid-, phenolic-acid-, and related analytes was monitored. The panel comprised gallic acid, 3,4-dihydroxybenzoic acid, protocatechualdehyde, 4-hydroxybenzoic acid, phthalic acid, catechin, vanillic acid, caffeic acid, syringic acid, epicatechin, dihydromyricetin, vanillin, p-hydroxycinnamic acid, syringaldehyde, rutin, vitexin, trans-ferulic acid, salicylic acid, sinapic acid, quercetin 3-β-D-glucoside, luteoloside, (+)-dihydroquercetin, genistin, benzoic acid, kaempferol-3-O-glucoside, (+)-dihydrokaempferol, resveratrol, daidzein, luteolin, quercetin, hydrocinnamic acid, trans-cinnamic acid, naringenin chalcone, phloretin, apigenin, naringenin, kaempferol, isorhamnetin, isoliquiritigenin, and gossypol. A matched reference-standard mixture was analyzed to provide expected retention times and accurate masses for the panel entries. Assignments required agreement with the corresponding reference-standard entry for these features; complete analyte-specific vendor metadata and sample-to-standard MS/MS traces were not retained in the supplied records. Sample signals are therefore described as targeted/putative annotations rather than fully orthogonally confirmed identities. The LC-MS material came from the same formulation batch administered to mice, and records zsqgy1-1, zsqgy1-2, and zsqgy1-3 represented three independent extractions from that single batch. The sample was extracted twice with 0.5 mL of 80% methanol-water containing 0.2% vitamin C, sonicated for 30 min, centrifuged at 12,000 rpm for 10 min, and the supernatants were combined. Separation used a Waters HSS T3 column (Waters Corporation, Milford, MA, USA; 50 × 2.1 mm, 1.8 micrometers) at 40 °C, 0.3 mL/min, and a 2-microliter injection. Mobile phases were 0.1% formic acid in water (A) and acetonitrile (B); the A/B program was 90:10 at 0–2 min, 40:60 at 6–9 min, and 90:10 at 9.1–12 min. A Vanquish UPLC-Q Exactive Orbitrap system (Thermo Fisher Scientific, Waltham, MA, USA) acquired negative-ion full scan-ddMS2 data from m/z 100–900 (spray voltage -2.8 kV; sheath gas 40 arb; auxiliary gas 10 arb; capillary 320 °C; heater 350 °C). Xcalibur 4.1 and TraceFinder 4.1 Clinical were used for acquisition and targeted processing. TraceFinder outputs were used to summarize peak response and software-reported injected-solution concentration. Three QC injections were used to assess short-term instrumental stability.

### 2.3. Animal Model and Experimental Design

**Figure 1:**
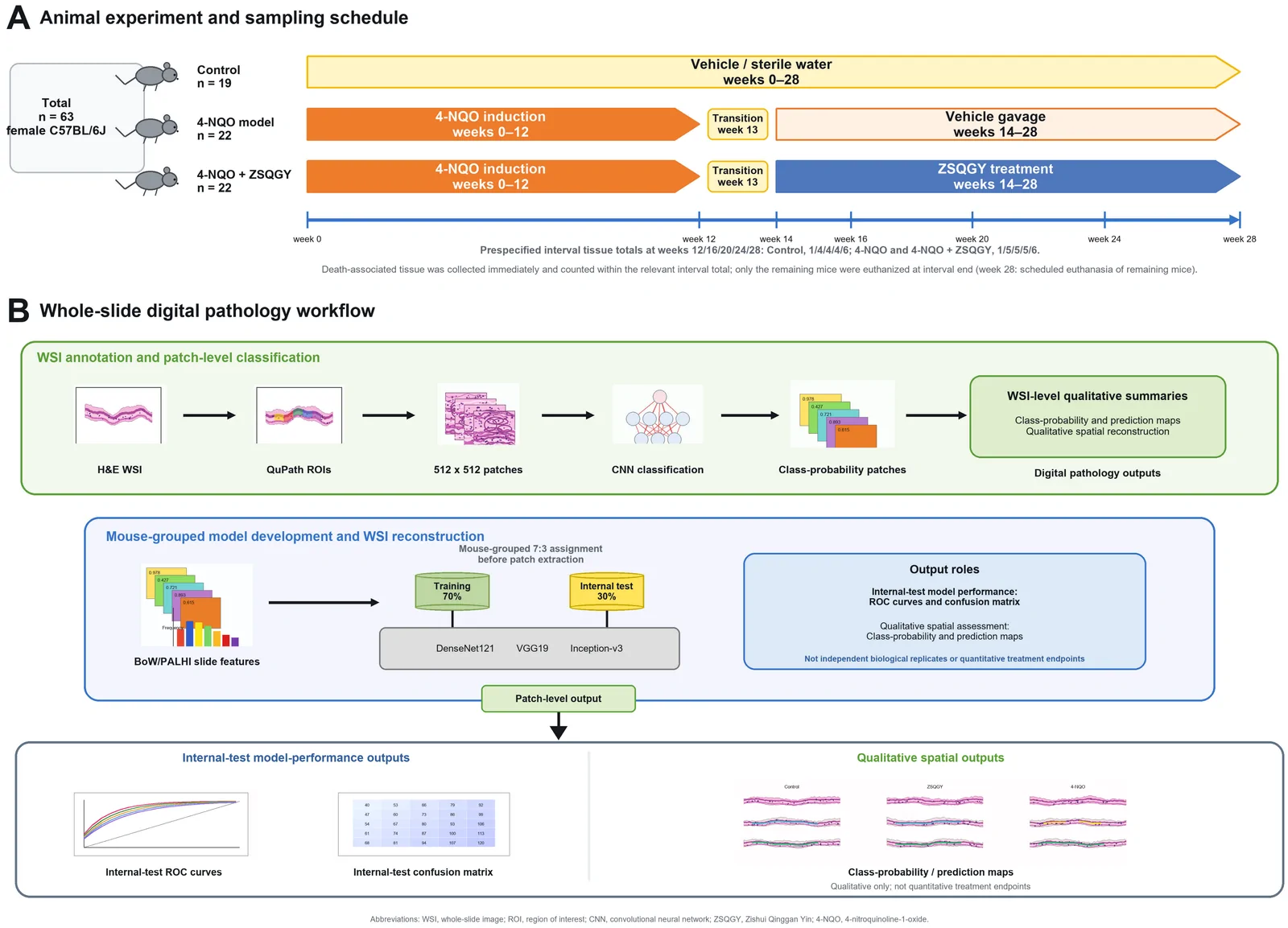
Study design and whole-slide digital pathology workflow. (**A**) Sixty-three female C57BL/6J mice were assigned to three groups: Control (n = 19), 4-NQO model (n = 22), and 4-NQO + ZSQGY treatment (n = 22). Control mice received sterile water throughout weeks 0–28. Model mice received 4-NQO during weeks 0–12 followed by a one-week transition and equal-volume sterile-water vehicle gavage during weeks 14–28, whereas the treatment group received 4-NQO induction followed by once-daily ZSQGY oral gavage during weeks 14–28. Prespecified collection intervals ended at weeks 12, 16, 20, 24, and 28, with target totals of 1/4/4/4/6 for Control and 1/5/5/5/6 for each 4-NQO-exposed group. A spontaneous death within an interval was followed by immediate tissue collection and preservation and counted toward that interval total; only the remaining number was euthanized at the scheduled collection point. Thus, mortality was not additional to the planned sampling totals. At the week-28 endpoint, mice remaining in the terminal allocation were euthanized as planned, and the six tissue sets per group were used for H&E/IHC, Western blotting, and qPCR, with each mouse treated as one independent biological replicate. (**B**) Whole-slide digital pathology workflow. The composite figure was assembled in Adobe Illustrator 2025. Mouse identifiers were retained through WSI scanning, QuPath ROI annotation, and patch extraction. The 7:3 model-development assignment was performed at mouse level before patch extraction. DenseNet121 patch-level outputs were reconstructed as qualitative spatial probability and prediction maps; treatment interpretation remained based on the consensus mouse-level pathology grade. WSI, whole-slide image; ROI, region of interest; CNN, convolutional neural network; ZSQGY, Zishui Qinggan Yin; 4-NQO, 4-nitroquinoline-1-oxide.

A 4-nitroquinoline-1-oxide (4-NQO)-induced murine model was used to reproduce the stepwise histopathological progression of esophageal precancerous lesions (Figure 1A) [19,20,21].

Sixty-three female C57BL/6J mice (SPF grade, 6–8 weeks old, 16 ± 1 g) were obtained from Vital River Laboratories (Beijing, China) and housed in individually ventilated cages under controlled temperature (22 ± 1 °C), humidity (50 ± 5%), and a 12 h/12 h light–dark cycle, with free access to food and water. After one week of acclimatization, mice were randomized into three groups: Control (n = 19), receiving sterile water throughout the 28-week experiment; 4-NQO model (n = 22), receiving 4-NQO (100 μg/mL in drinking water; Sigma-Aldrich/Merck, St. Louis, MO, USA, #N8141-5G) during weeks 0–12 followed by equal-volume sterile water vehicle gavage during weeks 14–28; and ZSQGY treatment (n = 22), receiving the same 4-NQO induction followed by once-daily Zishui Qinggan Yin oral gavage (6.638 g/kg/day) during weeks 14–28. Fresh drinking solutions were prepared and replaced at least weekly. The supplied logs do not retain exact replacement dates, preparation volumes, or individual 4-NQO intake. Spontaneous mortality was 0/19 in Control, 5/22 in the 4-NQO model group (weeks 8, 18, 23, 25, and 27), and 2/22 in the ZSQGY group (weeks 13 and 23). Mice were euthanized by progressive-fill compressed CO2 followed by confirmation of death under the approved animal protocol.

The experimental timeline consisted of a carcinogen-induction phase (weeks 0–12), a one-week transition phase after 4-NQO withdrawal (week 13), and an intervention phase (weeks 14–28). Dynamic histologic sampling was organized into prespecified collection intervals ending at weeks 12, 16, 20, 24, and 28. The target collection totals were 1/4/4/4/6 mice for the Control group and 1/5/5/5/6 mice for the 4-NQO model and ZSQGY treatment groups across these intervals, respectively. These values represent the total numbers of mice contributing tissue within each interval. If a mouse died spontaneously during an interval, tissues were collected and preserved immediately, the mouse was counted within that interval’s target, and the number euthanized at the scheduled end of the interval was reduced accordingly; deaths were therefore not added to the planned collection totals. Exploratory H&E assessment from the first collection interval was used to evaluate lesion development, and 4-NQO administration was discontinued at week 12 according to schedule. Mice then underwent a one-week transition before ZSQGY, or vehicle gavage was initiated at week 14. At week 28, the mice remaining in the terminal allocation were euthanized as planned. The terminal collection set comprised six mice per group; esophageal tissues were partitioned for H&E/IHC, Western blotting, and qPCR, with each mouse treated as one independent biological replicate. All animal procedures complied with ARRIVE 2.0 guidelines [22] and were approved by the Institutional Animal Care and Use Committee of Hebei Medical University (IACUC-Hebmu-2023021).

#### Study Workflow

The study design and digital pathology workflow are summarized in Figure 1. Briefly, the study established a 4-NQO-induced murine model of esophageal precancer, stopped 4-NQO at week 12, and applied ZSQGY during the intervention phase. Interval-based H&E sampling followed prespecified collection accounting: tissues from spontaneous deaths were collected and preserved immediately and counted toward the relevant prespecified interval total, and only the remaining number was euthanized at the scheduled collection point. The terminal collection associated with the week-28 endpoint comprised six tissue sets per group for histology, IHC, Western blotting, and qPCR. Three pathologists independently reviewed H&E-stained whole-slide images. Disagreements referred to differences in categorical lesion-grade assignment and/or interpretation of epithelial versus inflammatory/reactive regions; they were resolved by joint discussion until consensus. No formal inter-rater coefficient for the pathology review was retained. For CNN development, mice were assigned at a 7:3 ratio to training and internal test subsets before patch extraction; all slides, ROIs, and patches from one mouse remained in the same subset. DenseNet121 was retained for downstream spatial inference, and patch-level outputs were reconstructed as probability and prediction maps. These maps were qualitative spatial adjuncts, whereas biological inference used one consensus pathological grade per mouse. Figure 1 was assembled in Adobe Illustrator 2025.

### 2.4. Body Weight, Survival, and General Condition

Body weight was measured weekly and summarized as mean ± SD using the observations recorded for the formal experimental cohort at each time point. The weekly *n* values describe records contributing to the corresponding body-weight summary and should not be interpreted as end-of-week survivor counts. Weights from mice that died were not carried forward into later weeks; the week-28 summary reflects measurements obtained at the scheduled endpoint. The extra backup animal maintained for contingency was not included in manuscript-facing body-weight statistics. Cage-side general condition was monitored throughout the experiment, including fur appearance, activity, responsiveness, food intake, defecation, body-weight change, and signs potentially related to gavage injury. Mortality, immediate tissue collection after death, scheduled sampling, and other notable husbandry events were recorded prospectively. No formal composite clinical score or numerical 0–3 welfare scale was prespecified; these observations were recorded and interpreted descriptively.

### 2.5. Histopathological Evaluation

Paraffin-embedded esophageal tissues were sectioned (4 µm), deparaffinized, rehydrated, stained with hematoxylin and eosin, dehydrated, cleared, and mounted using a neutral mounting medium. The histology reagents and mounting medium were obtained from Servicebio (Wuhan, China).

Histopathological evaluation was conducted on H&E-stained sections using an Olympus BX63 microscope (Olympus Corporation, Tokyo, Japan). Whole-slide scanning was performed with a Pannoramic Slide Scanner (3DHISTECH Ltd., Budapest, Hungary) using a 20× objective to generate MRXS images. Original slide metadata showed a baseline pixel size of approximately 0.274 μm/pixel (range, 0.273810–0.274358 μm/pixel). Three pathologists who were not involved in the experimental design independently reviewed the squamous esophageal epithelium, and disagreements were resolved by joint discussion until consensus. Disagreements referred to categorical lesion-grade assignments and/or the distinction between epithelial and inflammatory/reactive areas; no formal inter-rater coefficient was retained. QuPath Polygon ROIs were assigned to Normal, Inflammatory, mild dysplasia (run-time source label: LGIN), moderate dysplasia (run-time source label: HGIN), severe dysplasia (run-time source label: Dysplasia), or carcinoma in situ (CIS). These source labels were retained in the model outputs because the folders were not renamed before model execution. Each WSI and annotated ROI retained the corresponding mouse identifier. For mice with mixed ROI grades, the highest consensus pathologist-assigned grade was used as the final mouse-level pathological grade. This highest-grade endpoint was selected as a conservative ordinal summary that assigns one independent outcome to each mouse and avoids pseudo-replication from multiple ROIs; lesion counts, lesion area, and the full grade distribution were not prespecified as validated mouse-level endpoints. The consensus ROI diagnosis served as the reference label for model development. ROIs were annotated in QuPath (version 0.3.2) [23].

### 2.6. Digital Pathology and Deep-Learning Model

H&E-stained WSIs were processed in QuPath, and epithelial regions were annotated using the Polygon tool. For CNN development and debugging, mice were assigned at a 7:3 ratio to training and internal test subsets before patch extraction. Cohort membership was maintained at the mouse level, and all slides, ROIs, and patches derived from one mouse remained in the same subset throughout model development. This model-development split was separate from the original animal treatment allocation and from subsequent mouse-level pathological analysis.

Annotated epithelial ROIs were segmented into non-overlapping 512 × 512-pixel patches at 0.5 µm/pixel. Patches consisting entirely of white background were excluded. Macenko color normalization was applied to reduce staining variation [24], and only patches derived from annotated ROIs were used for model development. Each retained patch remained linked to its source mouse, WSI, ROI, and source histologic label.

Each patch inherited the source histologic label of its annotated ROI: Normal, Inflammatory, mild dysplasia (LGIN), moderate dysplasia (HGIN), severe dysplasia (Dysplasia), or CIS. The 7:3 partition was used for mouse-grouped model training and internal testing/debugging; no mouse, WSI, ROI, or patch appeared in both subsets, and the original assignment was retained without subsequent reassignment. After CNN development, study specimens were analyzed according to the prespecified mouse-level pathological grading scheme. For mixed lesions, the highest consensus pathologist-assigned ROI grade defined the final mouse-level grade. Patch-level model results were not treated as independent biological replicates or treatment-effect estimates.

Z-score normalization was applied to the RGB channels. During training, online augmentation included random cropping and horizontal and vertical flipping. Internal test patches underwent the same deterministic normalization without random augmentation.

DenseNet121, VGG19, and Inception-v3 were initialized with ImageNet-pretrained weights and compared using the mouse-grouped training and internal test data. Reported patch-level measures included one-vs-rest AUC with 95% confidence intervals, class-wise sensitivity, specificity, positive predictive value, negative predictive value, and confusion matrices. Across the six classes in the available internal test outputs, the mean class-wise AUC/F1 values were 0.9328/0.6538 for DenseNet121, 0.9254/0.6002 for Inception-v3, and 0.9151/0.5639 for VGG19. DenseNet121 was retained for downstream spatial visualization in accordance with this retrospective model comparison; a separately prespecified architecture-selection criterion was not documented. Because the internal test subset was used within the same experimental workflow, these metrics were not interpreted as external diagnostic validation.

$$\eta_{t}=\eta_{min}^{i}+\frac{1}{2}\left(\eta_{max}^{i}-\eta_{min}^{i}\right)\left(1+cos\left(\frac{T_{cur}}{T_{i}}\pi \right)\right)$$

Models were trained for 128 epochs using stochastic gradient descent and softmax cross-entropy loss. A cosine-decay learning-rate schedule was used, with a reported maximum learning rate of 0.01 and a specified minimum of 0. Performance plots were generated with Matplotlib 3.3.4. The supplied records do not retain exact per-class counts of independent mice, slides, ROIs, or patches in the training and internal-test subsets, nor do they document a verified class-imbalance strategy. Accordingly, no class weighting, oversampling, or undersampling procedure is claimed. Other training-environment, checkpoint, multiclass-decision, and confidence-interval details were not available and were not inferred.

#### Heatmap Visualization and Lesion-Burden Mapping

For visual interpretation, two complementary outputs were generated. First, Grad-CAM was applied [25] to representative high-magnification fields to identify image regions contributing to DenseNet121 predictions. Second, class probabilities and final predicted labels were projected back to the original WSI coordinates to generate spatial probability and prediction maps. These maps were reviewed alongside the source H&E images and consensus annotations and were used as qualitative spatial summaries rather than quantitative treatment endpoints.

### 2.7. Network Pharmacology Analysis

The chemical-monitoring panel contained 40 predefined entries. For computational target prediction and network analysis, the two entries reported as not found in the administered formulation (naringenin chalcone and isoliquiritigenin) were excluded, leaving a revised 38-candidate computational panel. Structures of these 38 retained candidates were retrieved from PubChem [26]. Candidates with signals outside a provisional working range remained computational inputs but were not treated as quantitatively validated constituents. Potential targets were predicted with SwissTargetPrediction [27] and standardized to human proteins in UniProt [28]. ESCC-associated genes were compiled from GeneCards [29], NCBI Gene [30], and CTD [31]. Overlapping targets were submitted to STRING [32] (Homo sapiens; confidence > 0.4). Venn diagrams were generated with Venny 2.1. TCGA esophageal-cancer expression data [33] were filtered for ESCC and analyzed with limma [34] (*p* < 0.05; |logFC| > 0.585) as an auxiliary disease-expression resource. Cytoscape 3.8.0 [35] and MCODE [36] were used for network visualization and module screening. GO [37] and KEGG [38,39] enrichment was performed with clusterProfiler [40] using adjusted *p* < 0.05. Network outputs were treated as predictions for hypothesis generation, not evidence of compound activity, target engagement, or pathway causality [41,42].

### 2.8. Molecular Docking

Molecular docking was performed to explore plausible interactions between the revised 38-candidate computational panel and prioritized upstream pathway nodes. The two analytes reported as not found in the administered preparation, naringenin chalcone and isoliquiritigenin, were excluded from this ligand set. The retained candidates had differing levels of analytical support and were not interpreted as confirmed active constituents. PI3Kα (p110α) and AKT1 were selected as receptors based on the revised network-pharmacology results and the phosphorylation-based evaluation strategy. Protein and ligand structures were prepared using standard protonation, hydrogen-addition, and energy-minimization procedures. Docking was performed with AutoDock Vina 1.2.5 [43], and representative binding poses were inspected for binding-pocket occupancy, hydrogen-bond interactions, and hydrophobic contacts. Docking results were used only for mechanistic prioritization and were not interpreted as evidence of systemic exposure, direct target engagement, or therapeutic mediation.

### 2.9. Exploratory DARTS in Two Independent Tissue Cohorts

Exploratory ex vivo DARTS was conducted in two independent tissue-background datasets. The original model-background dataset used frozen esophageal tissues from the terminal collection interval associated with the week-28 endpoint (six mice in the 4-NQO model group) [44]. The six-mouse size reflected the available terminal tissue set allocated to this exploratory candidate screen, with one independently prepared lysate retained per animal for matched within-mouse comparisons; the assay was not powered as a confirmatory treatment experiment. The additional treatment-background dataset used esophageal tissues from six independently identified mice with documented prior in vivo ZSQGY treatment. Each independently prepared lysate represented one biological replicate and was divided into matched aliquots for within-mouse comparisons. Test materials were added only after tissue lysis; therefore, these assays evaluated ex vivo protease susceptibility rather than differences between in vivo treatment groups. Normal-control lysates were not included. The original model-background cohort consisted of 4-NQO-exposed mice that had received in vivo vehicle gavage, whereas the additional treatment-background cohort did not include a matched in vivo vehicle-treated DARTS cohort. Disease specificity and treatment-history effects were therefore not evaluated. For clarity, the original model-background cohort is designated Cohort A, and the additional prior-treatment cohort is designated Cohort B; mouse identifiers are cohort-specific. Tissues were homogenized on ice in a non-denaturing buffer containing 50 mM Tris-HCl (pH 7.5), 150 mM NaCl, 0.1% IGEPAL CA-630, 10% glycerol, and 1 mM DTT. These buffer components were obtained from Thermo Fisher Scientific (Shanghai, China). Protease inhibitors, phosphatase inhibitors, SDS, and sodium deoxycholate were omitted. After centrifugation at 16,000× *g* for 15 min at 4 °C, the supernatants were collected, quantified using a BCA protein assay kit (Thermo Scientific Pierce, Waltham, MA, USA), and adjusted to 1.0 mg/mL. Each lysate was divided into seven 45-μL aliquots containing 45 μg protein.

The original model-background run included no-Pronase, vehicle-plus-Pronase, ZSQGY (100 μg/mL dry-extract equivalent)-plus-Pronase, quercetin (50 μM)-plus-Pronase, luteolin (50 μM)-plus-Pronase, MK-2206 (10 μM)-plus-Pronase, and LY294002 (20 μM)-plus-Pronase conditions. For the LY294002-plus-Pronase treatment, LY294002 was obtained from Selleck Chemicals (Houston, TX, USA), and Pronase was obtained from Roche Diagnostics (Mannheim, Germany). The additional treatment-background run included no-Pronase, vehicle-plus-Pronase, ZSQGY at 50, 100, or 200 μg/mL-plus-Pronase, quercetin at 50 μM-plus-Pronase, and luteolin at 50 μM-plus-Pronase. The added 50, 100, and 200 μg/mL conditions were selected as an operational range bracketing the original 100 μg/mL screen condition; they were not derived from measured esophageal exposure, affinity constants, or a formal concentration-optimization study. Thus, the series was intended to explore whether a directional signal was reproducible across nearby ex vivo concentrations, not to establish an in vivo dose-response. In both datasets, the vehicle denotes the matched ex vivo incubation control and not an in vivo vehicle-treated animal group. Where DMSO was used, it was obtained from Sigma-Aldrich/Merck (Darmstadt, Germany), its final concentration was matched at 0.5%, with water and solvent volumes matched across conditions. The no-Pronase sample underwent the same vehicle incubation. Lysates were incubated with vehicle or test material for 60 min at 25 °C. MK-2206 and LY294002 were included only in the original model-background run.

Limited proteolysis was performed with Pronase from Streptomyces griseus (Sigma-Aldrich/Merck, St. Louis, MO, USA, P5147) at a Pronase-to-protein ratio of 1:1000 (*w*/*w*; 1 μg Pronase per mg protein) for 15 min at 37 °C. Reactions were immediately quenched by adding 4× reducing Laemmli buffer to a final concentration of 1×, followed by heating at 95 °C for 5 min. The no-Pronase sample was incubated for the same duration and at the same temperature without enzyme addition.

Twenty micrograms of protein per lane were separated on 8% SDS-PAGE gels and transferred to 0.45-μm PVDF membranes. Membranes were probed overnight at 4 °C with an AKT1-directed antibody (Cell Signaling Technology, Danvers, MA, USA, C73H10, #2938; 1:1000) and, in the separate PI3K assay, a PI3K p110α antibody (Cell Signaling Technology, Danvers, MA, USA, C73F8, #4249; 1:1000), followed by HRP-conjugated anti-rabbit IgG (Cell Signaling Technology, Danvers, MA, USA, #7074; 1:5000). The AKT signal was designated an AKT1-immunoreactive candidate band rather than an independently validated AKT1-specific band. Signals were acquired using a Bio-Rad ChemiDoc MP system (Bio-Rad Laboratories, Hercules, CA, USA) and Image Lab 6.1. The PI3K assay was analyzed as a separate exploratory dataset and was not pooled with either AKT dataset.

Before unblinding, the AKT candidate-band region was defined as 55–65 kDa and the PI3K p110α region as 110–120 kDa using molecular-weight markers. Identically sized target and local-background ROIs were applied across lanes within each membrane. Lane-specific selection of different upper or lower bands was not permitted, and doublets were not summed unless both bands had been independently validated as target-specific. For the original model-background cohort, AKT1-immunoreactive relative protection was calculated as the background-corrected treatment net integral divided by the same-mouse vehicle-plus-Pronase net integral (vehicle = 1). For the additional treatment-background cohort, the supplied workbook values were lane-integrated signals; no additional local-background subtraction was applied, and each treatment value was divided by the same-mouse vehicle-plus-Pronase value. The vehicle residual fraction was calculated as the vehicle-plus-Pronase signal divided by the matched no-Pronase signal. No-Pronase controls were used to inspect baseline signal and digestion but cannot remove biological confounding from differences in total AKT abundance, phosphorylation, protein complexes, or tissue composition. β-actin was evaluated only as a technical quality-control signal and was not used as the DARTS normalization denominator. DARTS was interpreted as an exploratory protease-protection assay, not as proof of direct binding, in vivo occupancy, or functional target dependence.

### 2.10. Western Blotting

Esophageal tissues from the terminal collection interval associated with the week-28 endpoint (Control, 4-NQO model, and ZSQGY-treated groups; *n* = 6 mice per group) were homogenized individually in ice-cold RIPA buffer (Beyotime, Shanghai, China) with protease/phosphatase inhibitor cocktails were obtained from Beyotime Biotechnology (Shanghai, China). Each mouse was treated as one independent biological replicate. Proteins were quantified by BCA, equal amounts were separated by SDS-PAGE, transferred to PVDF, blocked (5% BSA for phospho-targets; 5% milk for total proteins), and probed overnight at 4 °C with primary antibodies: p-AKT Ser473 and p-AKT Thr308 (Proteintech, Shanghai, China); all others from Abcam, Beijing, China (mTOR, p-mTOR Ser2448, S6K1, p-S6K1 Thr389, 4E-BP1, p-4E-BP1 Ser65, PTEN, cleaved caspase-3, and β-actin). HRP-conjugated secondary antibodies were obtained from Abcam (Beijing, China), and signals were visualized using an enhanced chemiluminescence reagent from Thermo Scientific Pierce (Waltham, MA, USA). Phospho-forms were detected first where applicable, membranes were stripped and reprobed for the corresponding total proteins, and total AKT was measured to permit calculation of p-AKT Ser473/AKT and p-AKT Thr308/AKT ratios; p/total ratios were quantified in ImageJ 1.54t. β-actin served as the loading control for non-phosphorylation-normalized targets, but its stability across experimental groups was not formally tested. The supplied antibody records do not retain complete catalog numbers, host species, lot numbers, or working dilutions for every Western-blot antibody; these metadata are therefore not inferred.

### 2.11. Immunohistochemistry

Paraffin-embedded esophageal sections from the terminal collection interval associated with the week-28 endpoint (n = 6 mice per group) were deparaffinized, rehydrated, and subjected to heat-induced epitope retrieval. EDTA buffer (pH 9.0) was used for phospho-epitopes, including p-AKT, p-mTOR, p-S6K1, and p-4E-BP1, whereas citrate buffer (pH 6.0) was used for PTEN, Ki-67, and cleaved caspase-3 according to antibody requirements. Endogenous peroxidase was quenched with 3% H2O2, and sections were blocked with 5% BSA. Slides were incubated overnight at 4 °C with primary antibodies against p-AKT (Ser473), p-mTOR (Ser2448), p-S6K1 (Thr389), p-4E-BP1 (Ser65), PTEN, Ki-67, and cleaved caspase-3, followed by incubation with HRP-conjugated secondary antibodies and development with DAB. Antigen-retrieval buffers, hydrogen peroxide, blocking reagent, and DAB reagent were obtained from Servicebio (Wuhan, China). Negative controls omitting primary antibody were included. IHC was performed on serial sections adjacent to H&E-stained slides, and ROIs were selected from dysplastic epithelial regions matched to histologic grades. Necrotic debris, luminal exfoliated cells, stromal inflammatory cells, and non-epithelial areas were excluded. For each mouse, 3–5 non-overlapping epithelial ROIs were analyzed, and the mean ROI value within each mouse was used as one biological replicate. H-score was calculated as 0 × P0 + 1 × P1 + 2 × P2 + 3 × P3 (range, 0–300), where Pi is the percentage of cells assigned intensity i; p-AKT, p-mTOR, p-S6K1, p-4E-BP1, PTEN, and cleaved caspase-3 were quantified by H-score [45]. Ki-67 labeling index was calculated as the percentage of positive epithelial nuclei among all counted epithelial nuclei. Reference [45] supports the scoring framework and was not used as evidence of antibody specificity. Quantification was performed by two blinded observers, with discrepancies resolved by consensus. All groups followed the same nominal fixation, retrieval, staining, and imaging workflow; exact fixation duration, processing dates, and complete antibody catalog/dilution records were not retained in the supplied files.

### 2.12. Quantitative Real-Time PCR

Esophageal tissues from the terminal collection interval associated with the week-28 endpoint (Control, 4-NQO model, and ZSQGY-treated groups; *n* = 6 mice per group) were processed for total RNA using TRIzol Reagent (Thermo Fisher Scientific, Waltham, MA, USA). RNA concentration and purity were assessed by NanoDrop (Thermo Fisher Scientific, Waltham, MA, USA), and RNA integrity was evaluated by Bioanalyzer/RIN (Agilent Technologies, Santa Clara, CA, USA). Typical A260/A280 values were approximately 1.9–2.0, A260/A230 values exceeded 2.0, and RIN values were generally 8.0–9.2. Each mouse was treated as one independent biological replicate. cDNA was synthesized with the High-Capacity cDNA Reverse Transcription Kit (Thermo Fisher Scientific, Waltham, MA, USA) using 1 μg total RNA in a 20 μL reaction with the following program: 25 °C for 5 min, 55 °C for 15 min, and 85 °C for 5 min. qPCR was performed on a Bio-Rad CFX system (Bio-Rad Laboratories, Hercules, CA, USA) in 20 μL reactions containing 10 μL 2× master mix, 0.4 μL each of 10 μM forward and reverse primers. For quantitative PCR, the forward and reverse primers were synthesized by Sangon Biotech (Shanghai, China), and the PCR master mix was obtained from Thermo Fisher Scientific (Waltham, MA, USA). The primers were used at a final concentration of 0.2 μM, 2 μL cDNA, and nuclease-free water to volume. Cycling consisted of 95 °C for 1 min, followed by 41 cycles of 95 °C for 5 s and 60 °C for 20 s; melt curves were acquired from 65 to 95 °C in 0.5 °C increments. NTC and no-RT controls were included. PARP1, CCND1, PFKFB3, and AURKB were quantified relative to GAPDH using the 2^−ΔΔCt^ method [46,47], with the control group as calibrator; primer sequences are provided in Table 1. Each mouse was measured in three technical replicates, which were averaged before statistical analysis.

### 2.13. Statistical Analysis

Statistical analyses were performed using GraphPad Prism 10.1.2 and the analysis workflow supplied with the DARTS dataset. Continuous variables are presented as mean ± SD and were analyzed by one-way ANOVA followed by appropriate multiple-comparison testing when assumptions were met; otherwise, nonparametric tests were used. Ordinal pathological grades were analyzed using the Kruskal–Wallis test. For endpoint Western blotting, qPCR, and IHC analyses, each mouse was treated as one independent biological replicate (n = 6 mice per group). For IHC analyses, ROI-level values were first averaged within each animal before group-level statistics to avoid pseudo-replication. Technical repeats, when performed, were not counted as independent biological replicates. DARTS statistics were calculated separately for the original 4-NQO model-background cohort and the additional cohort with prior in vivo ZSQGY treatment. Within each cohort, treatment-versus-matched ex vivo vehicle comparisons were paired within mouse and evaluated with exact two-sided sign-flip tests; all sign assignments were enumerated. For the original AKT assay, Holm adjustment [48] was applied across ZSQGY 100 μg/mL, quercetin, luteolin, MK-2206, and LY294002. For the additional AKT assay, Holm adjustment was applied across ZSQGY 50, 100, and 200 μg/mL, quercetin, and luteolin. The overall difference among the three additional ZSQGY concentrations was evaluated by an exact within-mouse permutation Friedman test, and the three dose-pair contrasts used a separate Holm adjustment. Marginal 95% percentile bootstrap intervals for arithmetic means were obtained by resampling the six animals with replacement. These intervals were not multiplicity-adjusted. The two cohorts were not pooled, cross-paired, or formally compared because they represented different in vivo treatment backgrounds and separate exploratory datasets. Any nominal P value shown in the main text is identified as exploratory; complete unadjusted and Holm-adjusted results, individual values, sample SDs, and direction counts are provided in Supplementary Tables S10–S13 for Cohort A and Tables S15–S17 for Cohort B. Correlations between IHC markers and biological phenotypes were assessed using Spearman correlation for ordinal histologic grade and Pearson or Spearman correlation for continuous variables according to data distribution. *p* < 0.05 was considered statistically significant.

## 3. Results

### 3.1. Targeted Monitoring of the Predefined ZSQGY Analyte Panel

Three independently extracted aliquots from the same ZSQGY formulation batch used for animal dosing (analytical records zsqgy1-1, zsqgy1-2, and zsqgy1-3) were evaluated against a predefined panel of 40 flavonoid-, phenolic-acid-, and related analytes. TraceFinder outputs provided expected and detected accurate mass, retention time, peak response, and software-reported injected-solution concentration for each targeted entry. The absolute mass errors for reported sample signals were within approximately 3.33 ppm. Across three QC injections, analyte-specific RSD values ranged from 0.21% to 5.77%, supporting short-term instrumental stability during the analyzed sequence (Table S2). Agreement among the three independent extractions was summarized as within-batch extraction and analytical repeatability.

**Figure 2:**
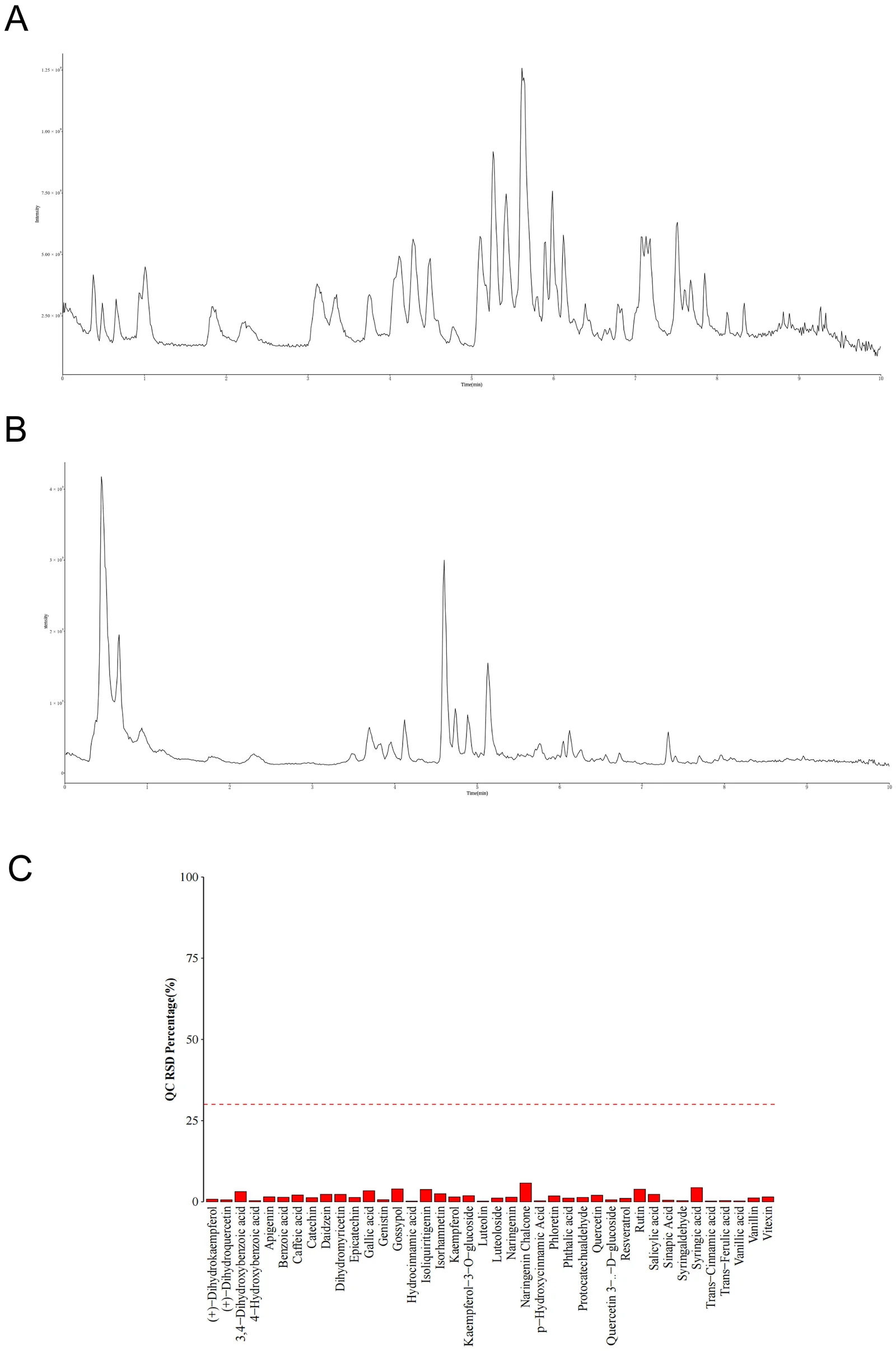
Targeted UPLC-Q Exactive/MS monitoring of a predefined ZSQGY analyte panel and short-term QC assessment. (**A**) TIC chromatogram of the standard mixture. (**B**) Representative TIC chromatogram from one of three independently extracted aliquots of the same ZSQGY formulation batch used for animal dosing. (**C**) RSD distribution across 40 targeted analytes calculated from three QC injections. The red dashed line indicates the vendor’s 30% threshold. The QC results summarize short-term instrumental stability during this analytical sequence. TIC, total ion chromatogram; QC, quality control; RSD, relative standard deviation.

Targeted monitoring yielded software-reported signals for 38 of the 40 predefined analyte entries (Figure 2; Tables S2–S4). Representative signals within the vendor-reported working ranges included rutin, trans-ferulic acid, quercetin, naringenin, kaempferol, and isorhamnetin. Gallic acid, catechin, and benzoic acid produced responses above the corresponding working ranges, whereas luteoloside, genistin, resveratrol, daidzein, luteolin, hydrocinnamic acid, phloretin, and apigenin produced values below them. Naringenin chalcone and isoliquiritigenin were reported as not found. These results characterized the targeted analyte profile of the administered formulation batch and informed candidate-compound selection for hypothesis generation.

### 3.2. Histopathological and Digital-Pathology Evaluation

#### 3.2.1. General Condition, Body Weight, and Mortality

**Figure 3:**
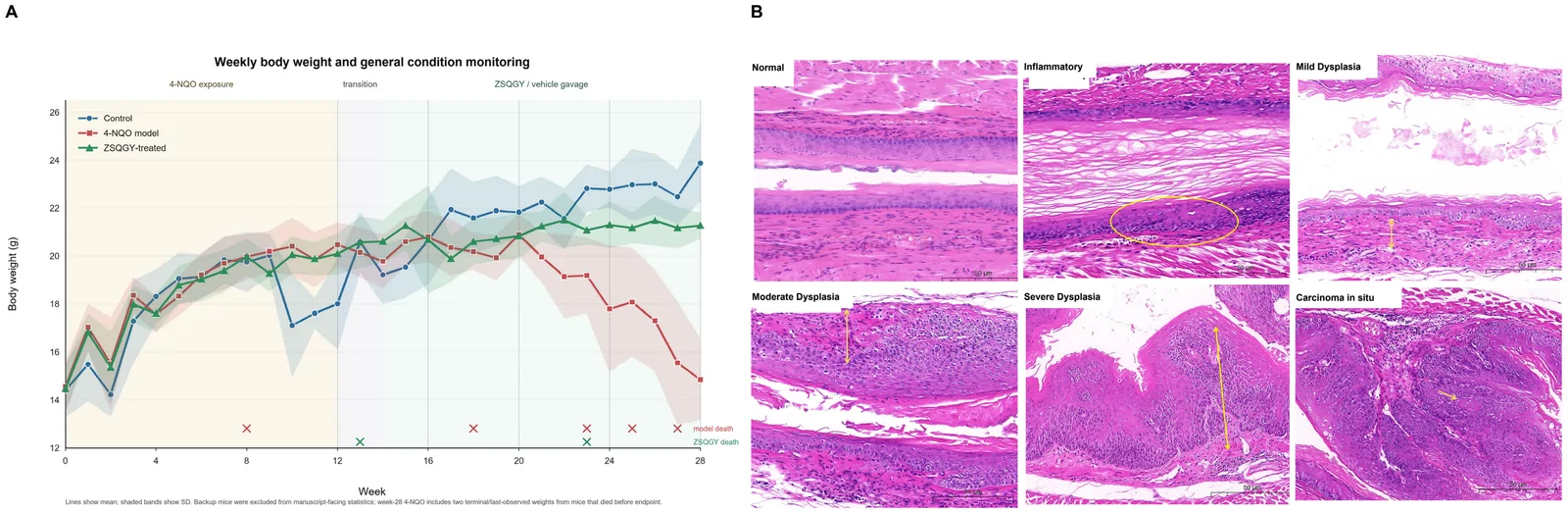
Body-weight monitoring and representative histopathological spectrum of esophageal epithelial lesions in mice. (**A**) Weekly body-weight trajectories in Control, 4-NQO model, and ZSQGY-treated mice. Data are shown as mean ± SD from the weekly source summaries; *n* denotes the number of body-weight records represented at that time point and not the end-of-week survivor count. Weights from mice that died were not carried forward, and the week-28 summary represents measurements obtained at the scheduled endpoint. Vertical dotted lines indicate prespecified tissue-collection points, and cross marks indicate weeks with recorded deaths. Death-associated tissues were collected and preserved immediately and counted within the relevant interval total. (**B**) Representative H&E-stained histopathological sections showing Normal, Inflammatory, Mild Dysplasia, Moderate Dysplasia, Severe Dysplasia, and Carcinoma in situ lesions. The yellow circle indicates inflammatory infiltration, and yellow arrows indicate epithelial thickening or dysplastic regions. Scale bars = 50 μm.

General condition and body weight were monitored weekly as descriptive tolerance measures. Control mice maintained stable appearance and activity, whereas 4-NQO-exposed mice developed variable late-phase deterioration. ZSQGY-treated mice showed less pronounced late deterioration in the observed cohort. The body-weight curves were treated as descriptive because group composition changed through prespecified interval sampling and mortality. Deaths occurred in the 4-NQO model group at weeks 8, 18, 23, 25, and 27 and in the ZSQGY group at weeks 13 and 23. For every spontaneous death, tissues were collected and preserved immediately, and the mouse was counted toward the corresponding interval’s target total; the number euthanized at the scheduled end of that interval was reduced accordingly. The week-28 body-weight summary represents measurements obtained at the scheduled endpoint and does not include carried-forward weights from mice that died earlier (Figure 3A; Table S5). Necropsy attribution was unavailable, so no specific cause was assigned.

#### 3.2.2. Histopathological Assessment

H&E-stained esophageal sections were reviewed independently by three pathologists, with disagreements resolved by joint discussion until consensus. ROIs were assigned to Normal, Inflammatory, mild dysplasia (run-time source label: LGIN), moderate dysplasia (HGIN), severe dysplasia (Dysplasia), or CIS. Representative examples are shown in Figure 3B. Consensus ROI diagnoses served as model-development reference labels, and the highest consensus ROI grade was used as the final mouse-level pathological grade when mixed grades were present.

#### 3.2.3. Patch-Level Model Performance

**Figure 4:**
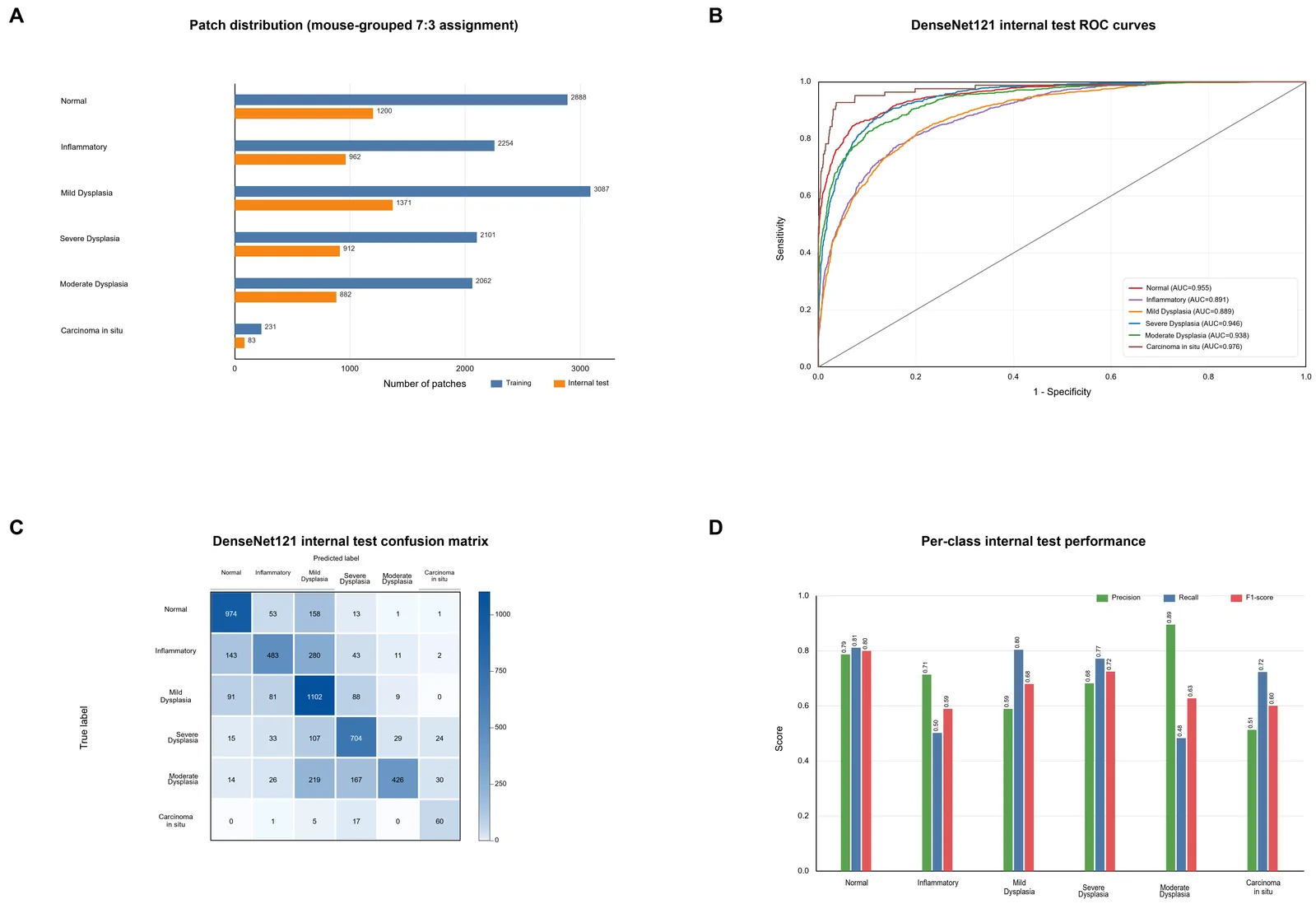
Patch-level performance of the DenseNet121 histologic classifier using mouse-grouped training and internal test subsets. (**A**) Patch distributions resulting from the 7:3 mouse-level assignment performed before patch extraction; the allocation method was not confirmed as random. (**B**) One-vs-rest ROC curves in the internal test subset for Normal, Inflammatory, mild dysplasia (run-time label LGIN), moderate dysplasia (run-time label HGIN), severe dysplasia (run-time label Dysplasia), and CIS. (**C**) DenseNet121 internal test confusion matrix; diagonal cells indicate correctly classified patches. (**D**) Per-class precision, recall, and F1-score. The 30% subset was an internal model-development test subset, not an independent validation cohort; AUC confidence intervals and other patch-level metrics are not mouse-level estimates. CNN, convolutional neural network; ROC, receiver operating characteristic; AUC, area under the curve; CIS, carcinoma in situ.

Whole-slide images were segmented into non-overlapping 512 × 512-pixel patches for patch-level histologic classification. DenseNet121, VGG19, and Inception-v3 were evaluated using mouse-grouped training and internal test subsets; the 7:3 assignment occurred before patch extraction, and all data from one mouse remained in one subset. The complete model-comparison results are provided in Supplementary Figure S1 and Table S6. DenseNet121 was retained for downstream spatial visualization based on the reported patch-level model-development results. In the internal test subset, its one-vs-rest AUC values were 0.955 for Normal, 0.891 for Inflammatory, 0.889 for mild dysplasia, 0.938 for moderate dysplasia (run-time source label: HGIN), 0.946 for severe dysplasia (run-time source label: Dysplasia), and 0.976 for CIS (Figure 4B). The confusion matrix showed most patches along the diagonal, with overlap among adjacent lesion categories (Figure 4C). Precision, recall, and F1-score were derived from the multiclass confusion matrix (Figure 4D). These are patch-level internal test results, not mouse-level treatment effects or external diagnostic performance estimates.

VGG19 and Inception-v3 also showed comparable internal patch-level AUC estimates. The internal test AUCs for VGG19 were 0.946 for Normal, 0.868 for Inflammatory, 0.836 for mild dysplasia, 0.925 for moderate dysplasia (HGIN), 0.946 for severe dysplasia (Dysplasia), and 0.970 for CIS. The corresponding Inception-v3 AUCs were 0.946, 0.883, 0.857, 0.934, 0.955, and 0.976, respectively (Supplementary Figure S1A). The DenseNet121 training confusion matrix contained 2888 Normal patches, correcting the previously reported value of 2890. Because model comparison and debugging occurred within the same experimental setting, the 30% subset was described as an internal test subset rather than an independent or external validation cohort.

#### 3.2.4. Heatmap and Whole-Slide Visualization

**Figure 5:**
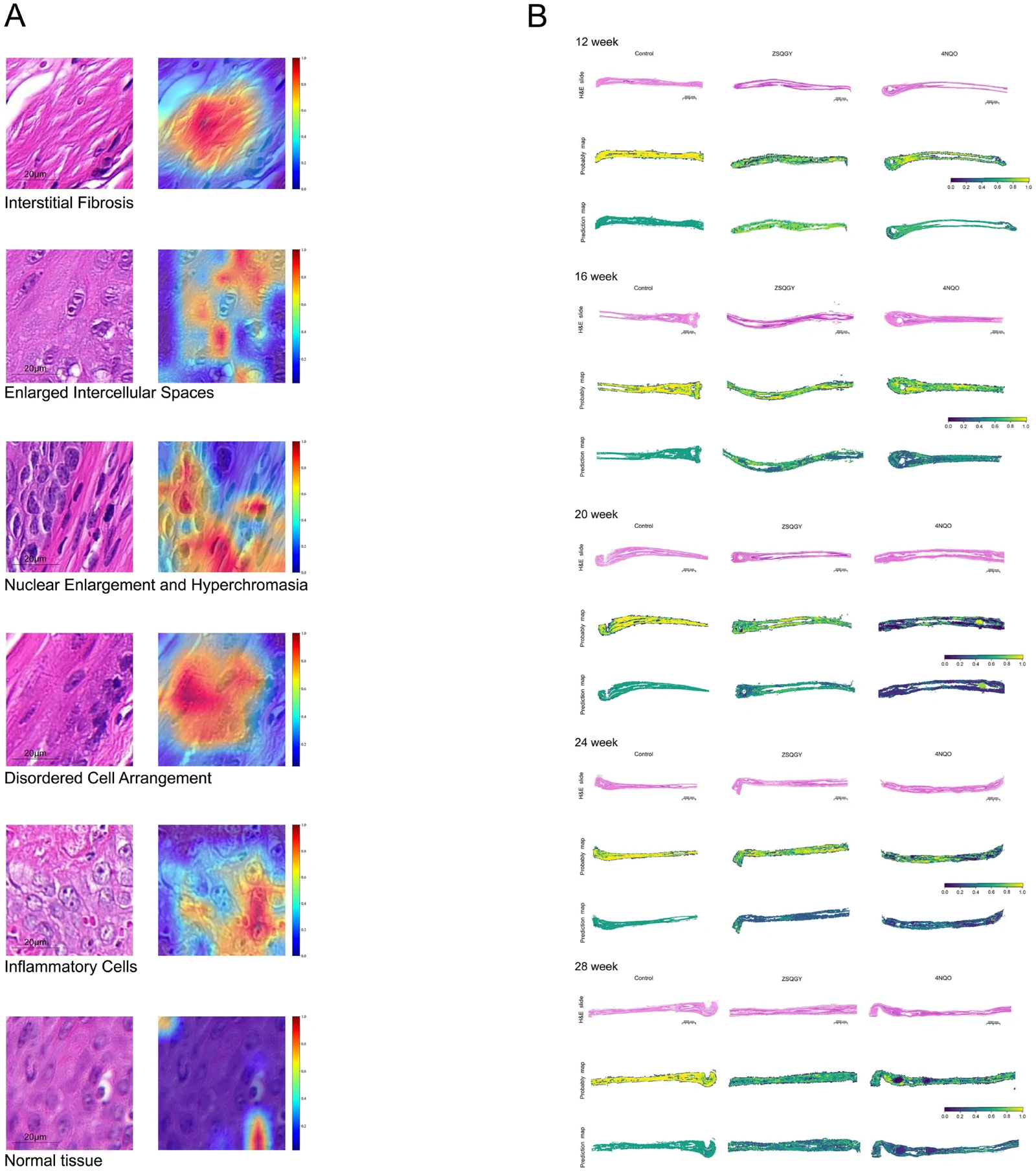
Visual interpretation and representative time-ordered spatial mapping of DenseNet121-based histologic predictions. (**A**) Representative H&E fields and corresponding Grad-CAM heatmaps showing model-attended regions. Warmer colors indicate greater model attention. (**B**) Representative whole-slide spatial visualization across weeks 12, 16, 20, 24, and 28 in Control, ZSQGY-treated, and 4-NQO groups. Probability maps represent class-specific model confidence on a 0–1 scale; prediction maps show the final class assigned to each patch. These maps provide qualitative spatial visualization and were interpreted together with pathologist-defined mouse-level grades rather than as independent biological replicates or quantitative treatment endpoints.

To visualize model behavior at both local and whole-slide levels, two complementary strategies were used. First, Grad-CAM-based local heatmaps were generated for representative high-magnification H&E fields to highlight image regions contributing to DenseNet121 classification. These heatmaps showed that model attention was concentrated around diagnostically relevant epithelial and stromal features, including enlarged intercellular spaces, nuclear enlargement and hyperchromasia, disordered epithelial arrangement, inflammatory cell infiltration, fibrotic change, and normal tissue architecture (Figure 5A).

Second, patch-level outputs were reconstructed onto the original WSI coordinates to generate spatial probability maps and prediction maps. In probability maps, color represented model confidence for a given diagnostic category: deep blue/purple indicated low probability, blue-green indicated intermediate probability, yellow-green/yellow indicated high probability, and white indicated background, blank regions, or excluded non-tissue areas. In prediction maps, color represented the final predicted category for each patch rather than probability magnitude; the model assigned each patch to the class with the highest predicted probability among Normal, Inflammatory, Mild Dysplasia, Moderate Dysplasia, Severe Dysplasia, and Carcinoma in situ. Large regions of the same color indicated spatially consistent predictions, whereas locally mixed colors suggested classification uncertainty or histologic heterogeneity (Figure 5B).

#### 3.2.5. Time-Ordered Lesion Mapping

Histopathological review and digital pathology visualization showed esophageal morphological changes across groups beginning at week 12. Week-12 exploratory H&E assessment in the 4-NQO-exposed groups showed early epithelial alterations consistent with lesion development, after which 4-NQO exposure was stopped, and mice entered the planned transition and intervention phases. During follow-up at weeks 16, 20, 24, and 28, the 4-NQO model group showed progressively abnormal epithelial regions in the time-ordered H&E sections and representative spatial maps, whereas the ZSQGY-treated group showed a lower-grade lesion pattern in the evaluated mouse-level specimens. Digital outputs were interpreted qualitatively alongside the consensus mouse-level pathology grade and were not analyzed as independent patch-level treatment endpoints. At the week-28 interval, CIS was observed among the evaluated 4-NQO specimens, whereas the evaluated ZSQGY specimens showed a lower-grade lesion pattern. The histopathological characteristics were consistent with published 4-NQO model observations [20,21].

### 3.3. Network-Based Target and Pathway Prioritization

During revision, the primary network analysis was restricted to the 38-candidate panel after exclusion of naringenin chalcone and isoliquiritigenin. Of these candidates, 34 contributed archived prediction records. The retained panel should not be interpreted as 38 confirmed active or quantitatively validated constituents. The original 40-candidate analysis was retained only as an explicitly labelled comparison.

#### 3.3.1. Network and Enrichment Analyses

**Figure 6:**
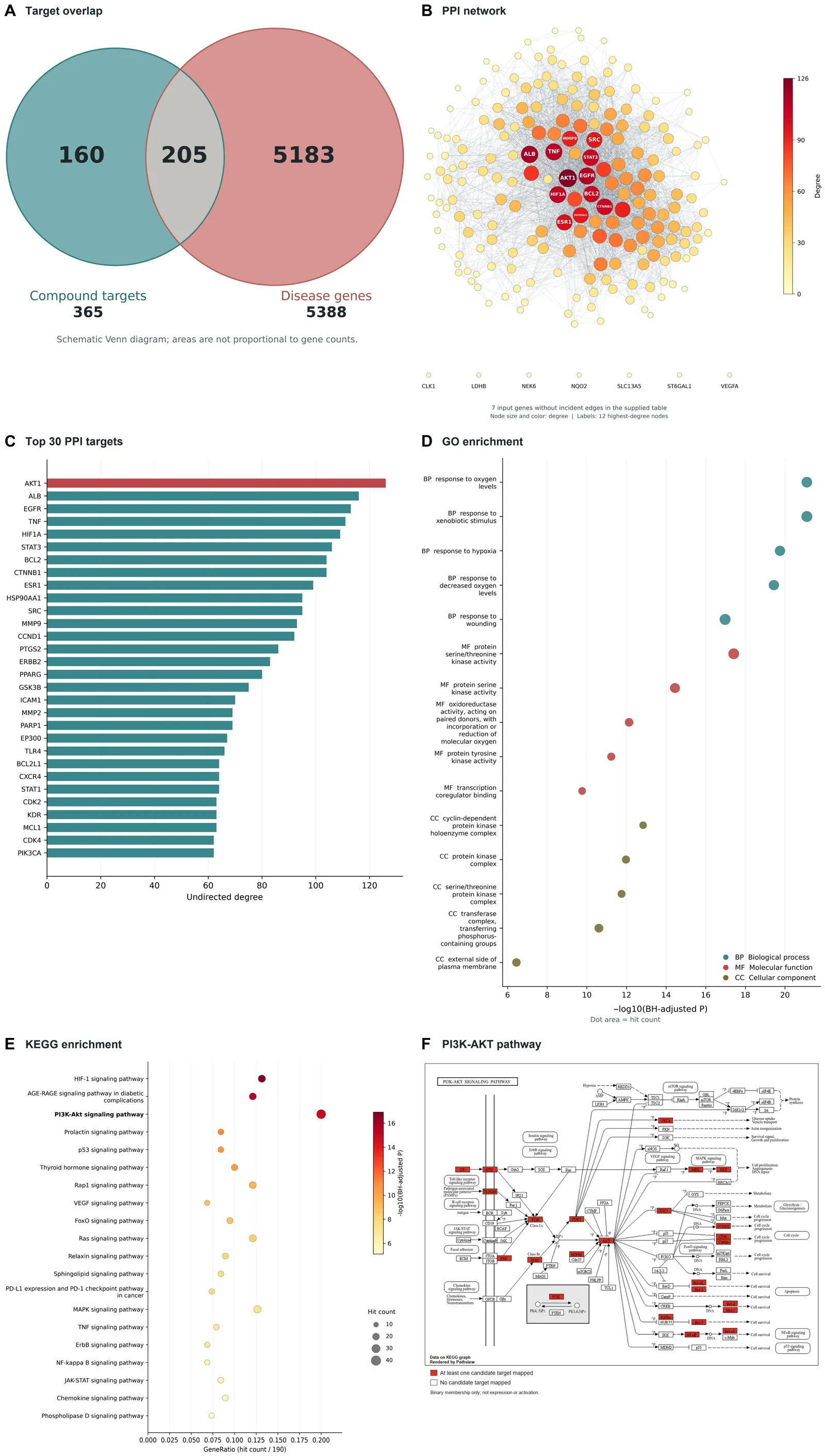
Revised hypothesis-generating network analysis after exclusion of two analytically unsupported candidates. (**A**) Overlap of 365 predicted drug-associated genes with 5388 ESCC-associated genes, yielding 205 common genes. The Venn diagram is schematic; circle and overlap areas are not proportional to gene counts. (**B**) Input-matched archived STRING graph containing 205 nodes and 3261 edges; seven input nodes lack incident edges in this supplied table. Node size/color encode degree, not a treatment response. (**C**) Top 30 PPI nodes by degree. (**D**) Top five GO terms per ontology under the frozen reanalysis. (**E**) The same 20 selected signaling pathways used in the archived KEGG display, with revised statistics; these are not the global top 20. Dot size represents hit count and color represents −log10(BH-adjusted P). (**F**) Human KEGG PI3K-AKT pathway hsa04151 rendered with Pathview using the revised 205-gene input. Red entries contain at least one mapped candidate gene; white entries contain none. Shared entries use maximum binary membership. The 38 mapped genes do not necessarily correspond to 38 graphical boxes. The colors do not encode expression changes or pathway activation. KEGG connector conventions are retained. The analysis does not establish active constituents, direct binding, target occupancy, or causality.

The revised network used the 38-candidate computational panel obtained after excluding the two analytical entries reported as not found. It contained 365 predicted drug-associated genes, of which 205 overlapped with the fixed 5388-gene disease set (Figure 6A). Exact input matching produced a PPI graph with 205 nodes and 3261 edges, including 198 nodes with incident edges in the archived table (Figure 6B). Its mean undirected degree was 31.815 (2E/N). AKT1 remained the highest-degree node (degree 126), followed by ALB (116) and EGFR (113); the top 30 nodes are shown in Figure 6C.

Six native MCODE modules were identified (Table S7). The highest-scoring module contained 45 nodes and 847 edges (score 38.500), including AKT1 and PARP1. Modules 2–6 contained 23/70, 23/51, 4/5, 4/5, and 16/22 nodes/edges, with scores 6.364, 4.636, 3.333, 3.333, and 2.933, respectively. All six memberships were identical between the input-matched baseline and exclusion analyses. The sixth module differed from the archived 17-node/24-edge module because COL18A1 was removed by input matching, not because two compounds were excluded. MCODE seeds are algorithmic starting nodes, not validated core targets. PARP1, TOP2A, CCNA1, PFKFB3, and AURKB remain descriptive network candidates; their historical experimental selection was not retrospectively redefined by this revision. Compound degrees ranked luteolin (64), isorhamnetin, kaempferol, and p-hydroxycinnamic acid (62 each), quercetin (61), and apigenin (60) highest (Table S8). These values reflect predicted connectivity rather than abundance, exposure, or contribution to the treatment effect.

Under the frozen reanalysis annotations, 1546 biological-process, 175 molecular-function, and 48 cellular-component terms met BH-adjusted *p* < 0.05 (Figure 6D). KEGG analysis identified 171 significant pathways among 352 eligible pathways. PI3K-AKT retained the same 38 candidate genes, with GeneRatio 38/190 and BH-adjusted *p* = 1.240 × 10^−15^; it ranked fifth in the full KEGG results. The selected signaling-pathway display is shown in Figure 6E. Differences from the historical enrichment totals reflect both annotation updates and candidate exclusion; the paired baseline analysis in the same frozen snapshot separates these effects. A smaller enrichment *p* value was not interpreted as a stronger treatment effect.

Among the enriched pathways, PI3K-Akt signaling provided a computational rationale for evaluating the proliferation-, survival-, metabolism-, and apoptosis-related phenotypes examined in this study. Considering enrichment strength, ESCC biological relevance, and feasibility of phosphorylation-based measurements, the PI3K/AKT/mTOR axis was selected for focused experimental evaluation. Accordingly, AKT, mTOR, S6K1, 4E-BP1, and PTEN were assessed as pathway-associated markers, while Ki-67 and cleaved caspase-3 were used as proliferation- and apoptosis-related phenotypic readouts. qPCR targets including CCND1, PFKFB3, AURKB, and PARP1 were selected as functional transcripts associated with cell-cycle progression, glycolytic metabolism, mitosis, and DNA-damage response.

#### 3.3.2. Pathway Mapping and Marker Selection

Mapping of the revised 205 overlapping predicted nodes onto the KEGG PI3K-Akt signaling pathway [38,39] showed that candidate nodes were distributed across upstream receptor signaling, PI3K/AKT activation, mTOR-mediated translational control [10,49], cell-cycle regulation, metabolic regulation, and apoptosis-related branches (Figure 6F). This distribution supported PI3K-Akt signaling as a biologically relevant and experimentally tractable axis for focused evaluation, rather than demonstrating pathway mediation. Pathway-associated changes were assessed primarily by phosphorylation-to-total protein ratios for AKT Thr308 and Ser473, mTOR Ser2448, S6K1 Thr389, and 4E-BP1 Ser65. PTEN was included as an upstream negative regulator [50] of PI3K/AKT signaling. At the tissue level, serial-section IHC was used to localize p-AKT, p-mTOR, p-S6K1, p-4E-BP1 (Ser65), and PTEN within dysplastic epithelium. In the paired 11-pathway comparison, only hsa05205 lost a mapped gene (CTSL, 29 to 28 hits); the other ten maps, including PI3K-AKT, were unchanged under identical binary mapping. These revision analyses assess computational robustness and did not determine the timing or original selection of experimental markers.

Ki-67 [51] and cleaved caspase-3 [52,53] were used as phenotype-level readouts of epithelial proliferation and apoptosis, respectively. Correlation analyses assessed whether pathway-associated IHC measures varied with Ki-67 labeling, cleaved caspase-3 H-score, and histologic grade. CCND1 was included because cyclin D1 is linked to ESCC biology and cell-cycle control [54,55]. PFKFB3, AURKB, and PARP1 were retained as network-prioritized functional transcripts representing glycolytic, mitotic, and DNA-damage-related programs; none was treated as a direct ZSQGY target.

### 3.4. Exploratory Molecular Docking and DARTS Assessment

#### 3.4.1. Molecular Docking as Hypothesis Generation

**Figure 7:**
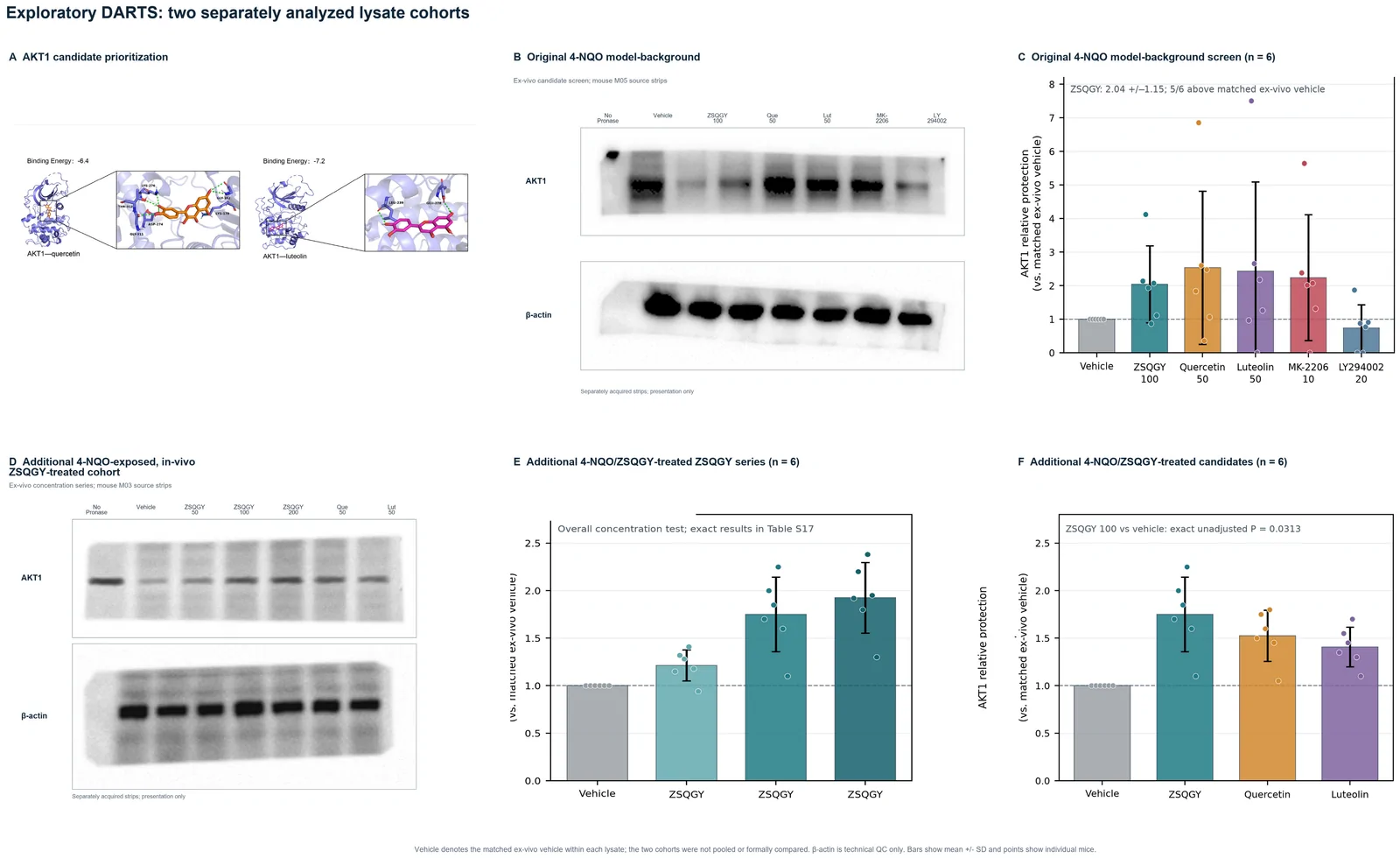
Exploratory DARTS in two independently analyzed tissue cohorts. (**A**) Representative docking poses of quercetin and luteolin with AKT1, used only for computational candidate prioritization. (**B**) Representative continuous seven-lane AKT1-immunoreactive and β-actin strips from the original 4-NQO model-background cohort (Cohort A, mouse M05), ordered as no Pronase, vehicle plus Pronase, ZSQGY 100 μg/mL, quercetin 50 μM, luteolin 50 μM, MK-2206 10 μM, and LY294002 20 μM. (**C**) Original Cohort A relative-protection values for all six biological samples; vehicle is the matched ex vivo control. (**D**) Representative AKT1-immunoreactive and β-actin strips from the additional 4-NQO-exposed, in vivo ZSQGY-treated cohort (Cohort B, mouse M03), ordered as no Pronase, vehicle plus Pronase, ZSQGY 50, 100, and 200 μg/mL, quercetin 50 μM, and luteolin 50 μM. (**E**) Cohort B ZSQGY concentration-series values for all six biological samples; the overall Friedman test is reported in Supplementary Table S16. (**F**) Cohort B descriptive candidate-compound comparisons. Bars show arithmetic mean ± SD and points show individual mice. β-actin was used only as a technical quality-control signal and was not the DARTS normalization denominator. The cohorts were not pooled, cross-paired, or formally compared. The principal nominal vehicle comparison shown in the main text is ZSQGY 100 μg/mL versus matched ex vivo vehicle (exact unadjusted *p* = 0.0313); complete exact and Holm-adjusted results are provided in the Supplementary Tables. Mouse identifiers are cohort-specific and are not cross-cohort identifiers. These observations represent directional AKT1-immunoreactive protease-protection signals and do not establish direct binding, in vivo target occupancy, or causal target dependence.

PI3Kalpha (p110alpha) and AKT1 were the receptors in the historical docking workflow. The revised descriptive extract of the available docking table excludes naringenin chalcone, isoliquiritigenin, the out-of-panel Morin record, and discordant duplicate Vitexin-AKT1 entries and reports only unambiguous retained-candidate records (Table S9); missing scores remain unavailable. No new docking scores or poses were generated. The archived source contained incomplete coverage and a table-to-figure score-assignment discrepancy requiring the original pose/log files. Accordingly, complete-screen rankings and definitive score-to-pose assignments are not asserted. Representative historical poses (Figure 7A; Figure S2) remain exploratory illustrations of the reported docking workflow [56,57], not independently verified evidence of binding. Experimental data in the accompanying panels were not modified.

#### 3.4.2. Two Independent Exploratory DARTS Datasets Show Directional AKT1-Immunoreactive Protease Protection

The DARTS findings were obtained from two separately analyzed six-mouse cohorts, designated Cohort A (original 4-NQO model-background/vehicle-gavage lysates) and Cohort B (additional prior in vivo ZSQGY-treatment lysates). In the original 4-NQO model-background cohort, ex vivo ZSQGY at 100 μg/mL increased the AKT1-immunoreactive relative-protection signal in five of six lysates (mean 2.04 ± 1.15; bootstrap 95% CI 1.31–2.95). Directionally similar increases were observed with quercetin (2.53 ± 2.28; 5/6), luteolin (2.43 ± 2.66; 4/6), and MK-2206 (2.24 ± 1.87; 5/6), whereas LY294002 was above matched vehicle in one of six lysates (0.736 ± 0.695). The original cohort was an ex vivo candidate screen in 4-NQO model-tissue lysates; its complete exact and Holm-adjusted tests are provided in Supplementary Table S10, with individual-animal measurements in Supplementary Table S11 and assay-quality documentation in Supplementary Tables S12 and S13, and no AKT comparison remained significant after correction (Figure 7B,C). In the additional cohort from six mice with prior in vivo ZSQGY treatment, the supplied workbook values were lane-integrated signals and were analyzed without additional local-background subtraction. The matched vehicle-plus-Pronase signal represented 38–43% of the no-Pronase input (40.3 ± 2.1%), consistent with partial digestion under the tested condition (Figure 7D; Table S17). Mean AKT1-immunoreactive relative protection was 1.213 ± 0.164 at 50 μg/mL, 1.750 ± 0.392 at 100 μg/mL, and 1.925 ± 0.371 at 200 μg/mL; the corresponding marginal bootstrap 95% confidence intervals were 1.088–1.323, 1.450–2.017, and 1.642–2.177 (Figure 7E). Signals were above matched vehicle in five of six samples at 50 μg/mL and in all six at 100 and 200 μg/mL. The within-mouse concentration test and dose-pair results are reported in Supplementary Table S16. The principal exploratory comparison of 100 μg/mL ZSQGY with matched ex vivo vehicle yielded a nominal exact *p* = 0.0313; complete Holm-adjusted results are retained in Supplementary Table S16. Quercetin and luteolin were descriptive comparators (1.525 ± 0.270 and 1.408 ± 0.208, respectively; 6/6 above vehicle; Figure 7F). This pattern is therefore described as a directional concentration-related signal, not as a validated monotonic dose-response or an optimal concentration.

The original PI3K p110α DARTS experiment showed a directional increase after ex vivo ZSQGY in all six model-background lysates; its complete statistics and assay-quality documentation are retained in Supplementary Tables S10–S13. Only a minority of PI3K samples met the suggested partial-digestion window, target-band resolution was weak and nonspecific, and the LY294002 response was inconsistent; the PI3K result therefore remains technically inconclusive. The additional treatment-background dataset did not include MK-2206 or LY294002 and did not include a validated target-specific positive ligand. Across both cohorts, the DARTS observations should be interpreted as AKT1-immunoreactive protease-protection patterns under ex vivo conditions. Prior in vivo ZSQGY treatment in the additional cohort does not establish target occupancy because ligand dissociation may occur during tissue lysis, dilution, and incubation. Differences may also reflect total AKT abundance, phosphorylation, protein-complex composition, or tissue-cell composition. Normal-control lysates were not included, and the additional treatment-background cohort lacked a matched in vivo vehicle-treated DARTS cohort; therefore, disease-specific protection and a treatment-induced change relative to an in vivo control cannot be inferred. Source-image, exposure, and candidate-band identity QC limitations are documented in the Supplementary Materials and further support a cautious, exploratory interpretation.

### 3.5. Western-Blot Evaluation of Pathway-Associated Proteins

Western blotting was performed using esophageal tissues from the terminal collection interval associated with the week-28 endpoint (six mice per group) to evaluate PI3K/AKT/mTOR-associated protein signaling. Pathway activation was assessed by normalizing site-specific phosphorylated proteins to their corresponding total proteins. Compared with the 4-NQO group, ZSQGY treatment reduced p-AKT^T308/AKT and p-AKT^S473/AKT, indicating attenuation of AKT activation. Consistently, p-mTOR/mTOR and the downstream mTORC1 effector ratios p-S6K/S6K and p-4E-BP1^S65/4E-BP1 were also decreased in ZSQGY-treated tissues. These changes support pathway-associated suppression of the AKT-mTOR signaling axis in the endpoint esophageal tissue dataset.

**Figure 8:**
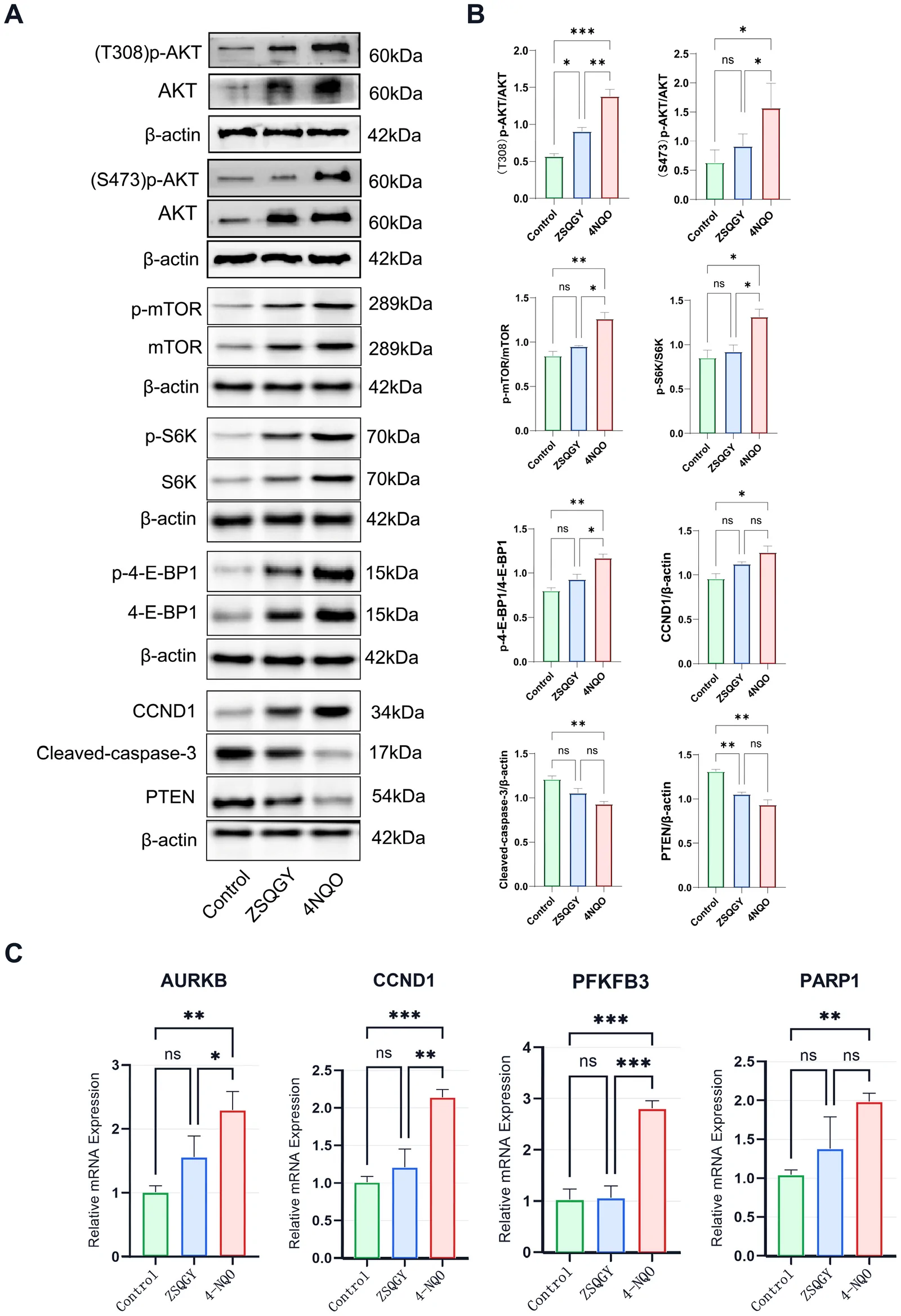
Western blot and qPCR validation of pathway-associated molecular changes in endpoint esophageal tissues. (**A**) Representative Western blots of esophageal tissue from Control, ZSQGY-treated, and 4-NQO model groups. Targets: p-AKT (Thr308/Ser473), AKT, p-mTOR/mTOR, p-S6K/S6K, p-4E-BP1 (Ser65)/4E-BP1, CCND1, cleaved caspase-3, and PTEN; β-actin, loading control. (**B**) Densitometry (mean ± SD; *n* = 6 mice/group): p/total ratios for AKT, mTOR, S6K, and 4E-BP1, plus CCND1, cleaved caspase-3, and PTEN, normalized to β-actin where applicable. Statistical significance is indicated as *, **, ***, or ns. (**C**) qPCR analysis of AURKB, CCND1, PFKFB3, and PARP1 mRNA expression in endpoint esophageal tissues (mean ± SD; *n* = 6 mice/group).

CCND1, PTEN, and cleaved caspase-3 were evaluated as functional or regulatory markers complementary to the phosphorylation readouts. These markers were interpreted together with the phospho/total protein ratios and the spatial IHC results, and non-significant comparisons were treated as supportive trends rather than definitive effects. Overall, the Western blot results provide bulk biochemical evidence consistent with reduced AKT-mTOR phosphorylation after ZSQGY treatment, whereas direct upstream PI3K target engagement requires further validation (Figure 8A,B).

### 3.6. Downstream Transcript Readouts

qPCR was performed using esophageal tissues from the terminal collection interval associated with the week-28 endpoint (six mice per group) to assess selected downstream or pathway-related functional transcripts. The analyzed genes included AURKB, CCND1, PFKFB3, and PARP1, representing mitotic regulation, cell-cycle progression, glycolytic metabolism, and DNA-damage response, respectively. Compared with the 4-NQO model group, ZSQGY-treated tissues showed reduced mRNA expression of AURKB, CCND1, and PFKFB3, whereas PARP1 showed a downward trend that did not reach statistical significance in the ZSQGY versus 4-NQO comparison. These transcriptional changes were broadly consistent with reduced proliferative and metabolic output downstream of AKT-mTOR-associated signaling.

Because PI3K/AKT/mTOR pathway activity is primarily regulated by protein phosphorylation rather than mRNA abundance, these qPCR results should be interpreted as downstream functional support rather than direct evidence of pathway inhibition. Together with the Western blot and spatial IHC data, the qPCR findings suggest that ZSQGY was associated with lower growth-, cell-cycle-, and glycolysis-related transcript output, together with a non-significant downward trend in PARP1 (Figure 8C).

### 3.7. Spatial IHC Evaluation

**Figure 9:**
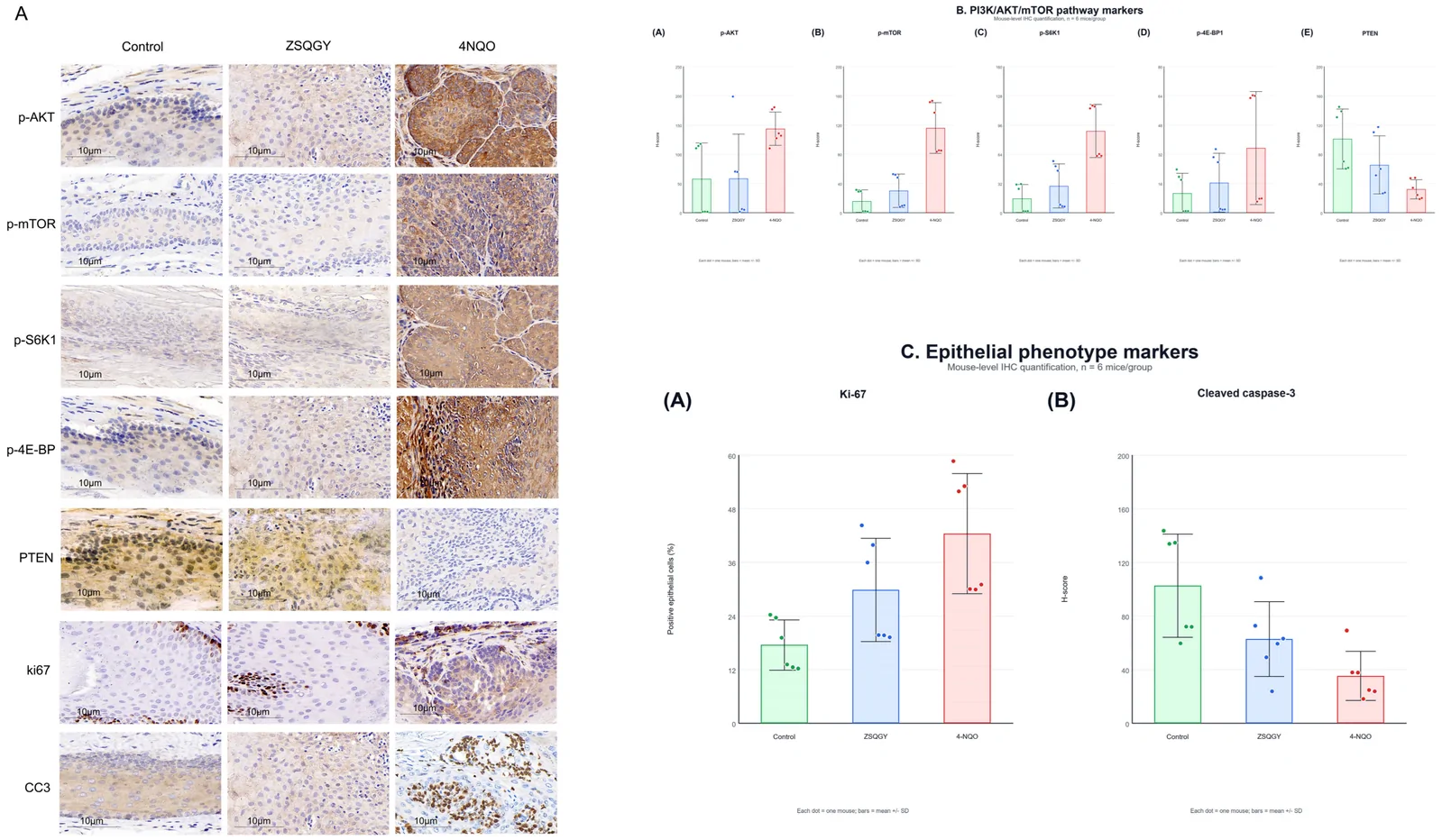
Expanded spatial IHC validation of PI3K/AKT/mTOR-associated signaling in esophageal precancerous lesions. (**A**) Representative IHC staining images of p-AKT, p-mTOR, p-S6K1, p-4E-BP1, PTEN, Ki-67, and cleaved caspase-3 (CC3) in terminal-interval esophageal tissues associated with the week-28 endpoint from Control, ZSQGY-treated, and 4-NQO model mice. DAB brown indicates positive immunostaining, and hematoxylin was used for nuclear counterstaining. Scale bar = 10 μm. (**B**) Mouse-level quantification of PI3K/AKT/mTOR pathway-associated markers, including p-AKT, p-mTOR, p-S6K1, p-4E-BP1, and PTEN, using H-score. (**C**) Mouse-level quantification of epithelial phenotype markers. Ki-67 was expressed as the percentage of positive epithelial cells, whereas cleaved caspase-3 was quantified by H-score. For panels (**B**,**C**), ROI-level measurements were first averaged within each mouse, and each dot represents one mouse. Bars show mean ± SD; *n* = 6 mice/group. ROI-level values were not treated as independent biological replicates.

Serial-section IHC was performed in esophageal tissues from the terminal collection interval associated with the week-28 endpoint (six mice per group) to determine whether the biochemical suppression of AKT-mTOR signaling was spatially localized to dysplastic epithelium. Compared with the 4-NQO group, ZSQGY-treated lesions showed lower H-scores for p-AKT (Ser473), p-mTOR (Ser2448), p-S6K1 (Thr389), and p-4E-BP1 (Ser65), together with a higher PTEN H-score. These changes indicate reduced pathway activity at both the AKT/mTOR core and mTORC1 downstream-output levels within lesional epithelial compartments. In the same matched ROIs, ZSQGY was associated with a lower Ki-67 labeling index and higher cleaved caspase-3 H-score, consistent with reduced proliferative activity and enhanced apoptosis-related epithelial turnover. p-AKT, p-mTOR, p-S6K1, p-4E-BP1, and PTEN were interpreted as spatial pathway readouts, whereas Ki-67 and cleaved caspase-3 were treated as phenotype markers rather than direct pathway-activity indicators. (Figure 9A–C).

Correlation analysis further supported spatial coherence between pathway activity and lesion phenotype. Animal-level IHC values were used for these analyses after averaging ROI-level measurements within each mouse. p-AKT and p-mTOR H-scores were positively correlated with Ki-67 labeling index, indicating that higher AKT/mTOR activity was associated with a more proliferative epithelial state. p-S6K1 and p-4E-BP1 H-scores were positively correlated with histologic grade, consistent with increased mTORC1 output in more advanced dysplastic lesions. In contrast, PTEN H-score was negatively correlated with histologic grade, whereas cleaved caspase-3 H-score was inversely correlated with Ki-67 labeling index. These relationships do not establish causality, but they strengthen the conclusion that ZSQGY treatment is accompanied by a coordinated spatial reduction in PI3K/AKT/mTOR signaling and proliferative output in dysplastic epithelium (Table S14).

## 4. Discussion

ESCC develops through a stepwise process from chronic epithelial injury and dysplasia to carcinoma in situ and invasive cancer. PI3K/AKT/mTOR signaling coordinates cell survival, growth, translation, and metabolism [9,10,11,12]. We evaluated whether ZSQGY-associated attenuation of 4-NQO-induced precancer progression occurred together with reduced activity of this signaling axis.

### 4.1. Integrative Interpretation

The consensus histopathological findings provide the phenotypic basis for this study. In the 4-NQO model, lesions followed the expected progression from inflammatory or reactive changes to dysplasia and CIS. ZSQGY treatment was associated with a lower-grade lesion pattern in the evaluated mouse-level specimens. The deep-learning analysis did not replace expert diagnosis; it supplied patch-level classification and qualitative whole-slide spatial visualization. Grouping by mouse prevented slide- and patch-level leakage between the model-development subsets, and subsequent biological comparisons used one consensus grade per mouse. Targeted chemical monitoring and network pharmacology then provided a hypothesis-generating framework for interpreting these tissue changes. UPLC-Q Exactive/MS analysis of three independently extracted aliquots from the administered formulation batch generated signals consistent with several predefined analytes, including rutin, trans-ferulic acid, quercetin, naringenin, kaempferol, and isorhamnetin. Network analysis linked candidate panel compounds to ESCC-associated targets and highlighted pathways related to cell-cycle progression, glycolytic metabolism, DNA-damage response, inflammation, and PI3K-AKT signaling. The original computational workflow informed focused experimental evaluation. The revision exclusion analysis retained AKT1 prioritization and the 38 PI3K-AKT mapped genes, but neither analysis establishes constituent-specific activity or mechanism.

The validation experiments supported this pathway-focused interpretation while preserving the distinction between an immunoreactive protease-protection signal and downstream pathway effects. The two DARTS datasets were analyzed separately. The original 4-NQO model-background screen showed directional AKT1-immunoreactive protection with ex vivo ZSQGY and reference compounds, whereas the additional cohort from mice with prior in vivo ZSQGY treatment showed a directional concentration-related pattern across ex vivo ZSQGY 50–200 μg/mL. The nominal exact P for the selected 100 μg/mL versus matched ex vivo vehicle contrast is reported for transparency in the Results, with complete multiplicity-adjusted results in the Supplementary Tables. The original PI3K p110α assay remained technically inconclusive. These DARTS observations complement, but do not mechanistically establish, the separate endpoint phosphorylation, histologic, and transcript findings. Separately, Western blotting in the terminal-interval esophageal tissue set showed reduced phosphorylation of AKT at Thr308 and Ser473, mTOR, S6K1, and 4E-BP1 Ser65, together with PTEN-associated and functional-marker changes. Expanded IHC localized the same pattern to dysplastic epithelium, where lower pathway-marker H-scores were accompanied by lower Ki-67 labeling and higher cleaved caspase-3 H-score. Animal-level correlations linked pathway activity with histologic grade and proliferation/apoptosis-related markers (Table S14), while selected transcripts showed lower cell-cycle-, glycolytic-, and mitotic-related output, together with a non-significant downward trend in PARP1.

Overall, the data support an association-based model in which ZSQGY is associated with a lower-grade 4-NQO-associated precancer lesion pattern together with AKT-centered PI3K/AKT/mTOR pathway modulation and lower proliferative output in dysplastic epithelium. The evidence is convergent across histology, time-ordered qualitative digital-pathology visualization, an exploratory AKT1-immunoreactive DARTS signal, protein phosphorylation, serial-section IHC, animal-level correlations, and selected transcript readouts. These layers do not show that ZSQGY directly binds AKT1, that PI3K is directly engaged, or that this pathway is the only or indispensable mediator of the observed phenotype.

### 4.2. Mechanistic Implications

Reduced phosphorylation of AKT at Thr308 and Ser473 is consistent with lower activation at its two major regulatory sites; Ser473 is regulated by mTORC2 [58]. Lower p-S6K1 and p-4E-BP1 Ser65 further indicated reduced mTORC1-associated output. The IHC correlations were directionally consistent with this interpretation, but correlation does not establish pathway mediation.

DARTS detects ligand-associated changes in protease susceptibility, but protection may reflect direct binding, conformational effects, or protein-complex stabilization [44]. Both independently analyzed cohorts showed directional AKT1-immunoreactive protease-protection patterns under ex vivo conditions. The additional cohort had a prior in vivo ZSQGY treatment history, but ligand dissociation during homogenization, dilution, and incubation could eliminate or alter any in vivo occupancy signal. Differences may also reflect total AKT1 abundance, phosphorylation, protein-complex composition, or tissue cellular composition; the no-Pronase input provides only partial control. The endpoint phosphorylation, histologic, and transcript comparisons were separate in vivo analyses and do not demonstrate that AKT1 protease susceptibility mediates the phenotype. No orthogonal binding assay, competition experiment, tissue-occupancy measurement, or AKT perturbation/rescue experiment was performed.

Quercetin, luteolin, kaempferol, and isorhamnetin were included in the targeted panel and docking analysis. These compounds belong to flavonoid classes widely described in natural products [59,60], while quercetin-specific structural evidence and broader review-level data support the plausibility of PI3K/AKT/mTOR-related effects for selected flavonoids in other experimental settings [56,61]. These external observations and the docking rankings do not show that the compounds were present at pharmacologically effective concentrations, systemically exposed, or responsible for the phenotype here. Direct binding, exposure, rescue, fractionation, and recombination studies are required before assigning constituent-specific roles.

### 4.3. Methodological Considerations

One strength of this study is the connection between computational prediction and experimental evaluation while preserving evidence levels. Mouse-grouped digital pathology supported patch classification and qualitative spatial visualization, while treatment interpretation remained at the mouse level. Targeted UPLC-MS monitoring characterized the administered formulation batch, network pharmacology and docking generated target hypotheses, exploratory DARTS assessed ligand-associated protease protection, and biochemical assays evaluated a focused signaling axis. Network analysis and docking prioritize hypotheses. DARTS provides an exploratory protein-protection signal but does not by itself establish direct binding or functional mediation. Western blotting measures bulk pathway-associated changes, serial-section IHC adds spatial localization within dysplastic epithelium, Ki-67 and cleaved caspase-3 reflect epithelial phenotype, and qPCR captures selected downstream programs. Keeping these evidence classes distinct avoids treating patches as biological replicates or converting computational predictions, protease protection, or expression changes into stronger causal claims than the designs support.

### 4.4. Limitations and Future Directions

Several limitations affect interpretation. The study used one 4-NQO model and one ZSQGY dosing schedule and chemically characterized only one formulation batch. Mouse-level grouping reduced digital-pathology leakage, but the reported 7:3 comparison remained an internal model-development assessment within one staining and scanning workflow; its independence may have been further limited if the 30% subset was inspected during debugging. Exact per-class mouse, slide, ROI, patch counts, and the class-imbalance strategy were unavailable, so these model metrics require cautious interpretation. Several training and confidence-interval details were unavailable, patch-class imbalance limits interpretation based on AUC alone, and external animal-level validation was not performed. The spatial maps were therefore used qualitatively. Prespecified interval sampling and mortality changed group composition over time and limit interpretation of the descriptive body-weight curves; however, weights from earlier deaths were not carried into the week-28 summary. Exact drinking-solution replacement dates, solution volumes, and individual 4-NQO intake were not retained. No formal composite clinical score was prespecified. Long-term stability of dissolved granules was not formally validated, and repeated gavage injury cannot be completely excluded despite same-day use, a blunted needle, and trained personnel. The DARTS datasets were exploratory and small (n = 6 per independent cohort). The original 4-NQO model-background cohort and the additional cohort from 4-NQO-exposed mice with prior in vivo ZSQGY treatment were not pooled or formally compared. Normal-control and matched in vivo vehicle-treated model lysates were unavailable, limiting assessment of disease specificity and treatment-history effects. The added 50–200 μg/mL concentrations were operational ex vivo conditions rather than measured tissue exposures or affinity estimates, and the added dataset lacked a validated positive reference ligand and a complete Pronase gradient. The original MK-2206 and LY294002 responses were directionally informative but not confirmatory, and the original PI3K p110α assay remained technically inconclusive because of target-band resolution, digestion-window, and reference-ligand limitations. Quantitative interpretation depends on antibody specificity, detector linearity, source-image provenance, molecular-weight assignment, and correspondence between image ROIs and integrated values; the added workbook was analyzed as supplied lane-integrated signal without extra local-background subtraction. Display panels do not substitute for quantitative source records. The additional treatment background does not prove in vivo target occupancy because tissue lysis and dilution can permit ligand dissociation, and no tissue-exposure measurements were available. Complete antibody metadata and exact fixation duration were not retained. No orthogonal target-engagement assay, pathway rescue, or independent-batch chemical-validation study was performed. External validation, a documented Pronase gradient, competition experiments, CETSA or biophysical binding assays [62], AKT perturbation/rescue, and constituent exposure studies are needed.

The chemical analysis focused on a predefined 40-analyte panel measured in three independent extractions from the formulation batch used for animal dosing. The observed targeted profile and short-term QC results provided batch-specific chemical characterization for this study. Future studies should extend the analysis to independently prepared production batches and evaluate plasma or tissue exposure to clarify the in vivo relevance of individual constituents.

**Figure 10:**
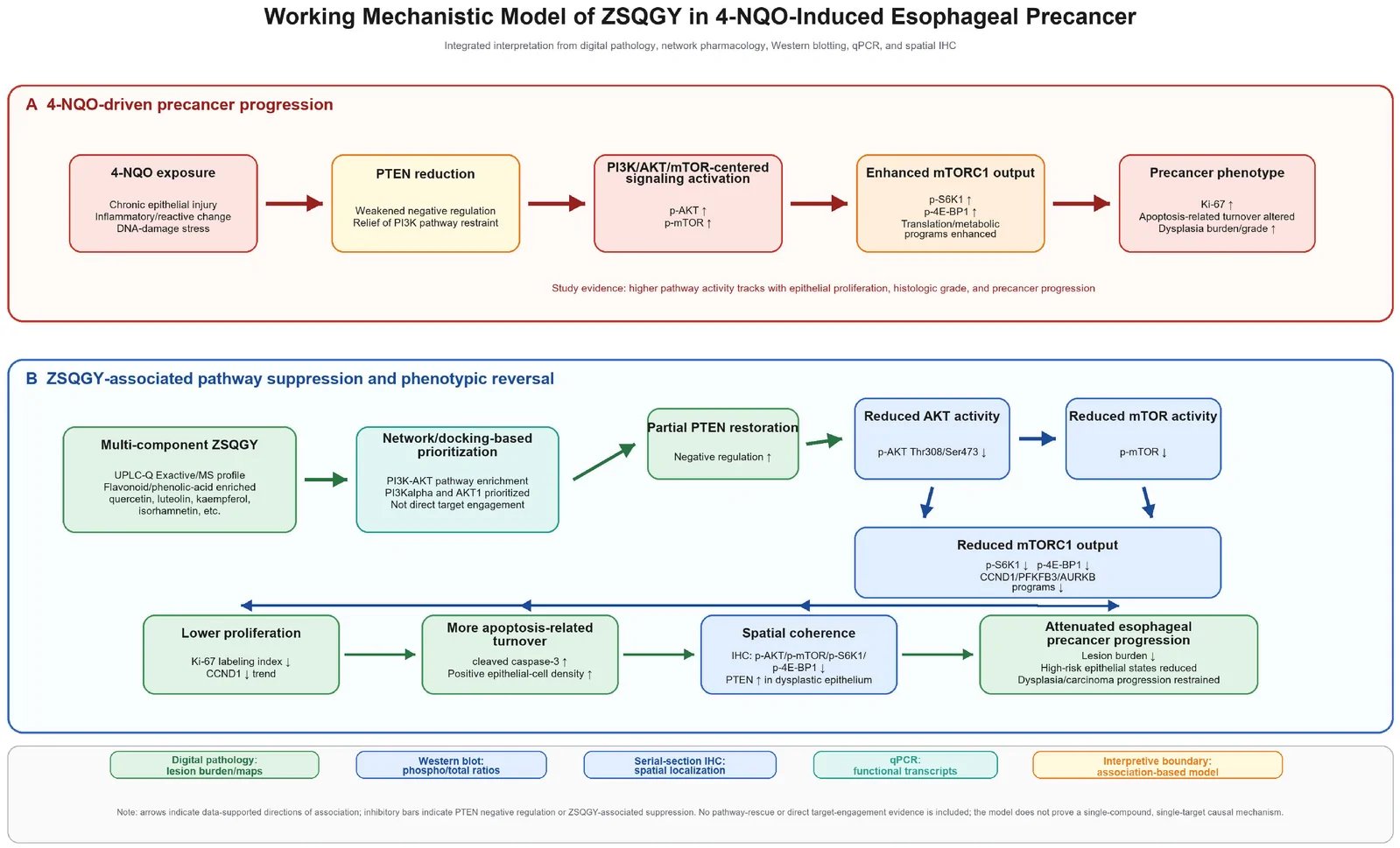
Working model of ZSQGY-associated attenuation of 4-NQO-induced esophageal precancer progression. (**A**) 4-NQO exposure was associated with chronic epithelial injury, inflammatory/reactive change, DNA-damage stress, PTEN-associated alterations, increased PI3K/AKT/mTOR pathway readouts, increased Ki-67, reduced cleaved caspase-3, and a higher-grade dysplastic lesion pattern. (**B**) Targeted UPLC-Q Exactive/MS monitoring characterized the administered formulation batch, while network analysis and docking prioritized AKT-centered PI3K/AKT/mTOR signaling. Exploratory DARTS provided a preliminary AKT1-immunoreactive protease-protection signal but did not establish direct binding or causal target engagement; PI3K p110α DARTS remained technically inconclusive. ZSQGY treatment was accompanied by decreased p-AKT, p-mTOR, p-S6K1, and p-4E-BP1 Ser65, PTEN-associated changes, reduced downstream functional programs, lower Ki-67 labeling, increased cleaved caspase-3 H-score, and spatially coherent changes within dysplastic epithelium. Arrows indicate data-supported directions of association rather than proven causal mediation. EPL, esophageal precancerous lesion; DARTS, drug affinity responsive target stability; IHC, immunohistochemistry; ZSQGY, Zishui Qinggan Yin; 4-NQO, 4-nitroquinoline-1-oxide.

This study focused mainly on epithelial signaling. The esophageal precancerous field also includes stromal and immune interactions and inflammatory signaling [63]. Spatial transcriptomics [64] and multiplex imaging [65] could provide a broader view of ZSQGY-associated changes in the precancerous microenvironment. Recent work on aberrant epithelial-cell interactions in ESCC further illustrates the value of resolving tissue-contextual signaling [66], while barrier and microbiome-related signals also warrant study. Pharmacokinetics, herb–drug interactions, and long-term safety require systematic evaluation. Figure 10 integrates the animal, digital-pathology, targeted chemical-monitoring, network, and molecular findings as an association-based working model rather than proof of a single-compound, single-target mechanism.

## 5. Conclusions

ZSQGY treatment was associated with a lower-grade lesion pattern in 4-NQO-exposed mice. Exploratory DARTS in the original 4-NQO model-background cohort and in an additional prior-treatment cohort showed directional AKT1-immunoreactive protease-protection patterns under ex vivo conditions. The two datasets were analyzed separately; neither establishes direct binding, in vivo target occupancy, disease-specific protection, or causal AKT1 dependence. The initial PI3K p110α assay remained technically inconclusive. Endpoint molecular and spatial readouts were consistent with AKT-centered PI3K/AKT/mTOR pathway modulation, lower Ki-67, higher cleaved caspase-3 staining, and changes in selected downstream transcripts. The study does not establish direct binding, in vivo target occupancy, active constituents, pathway necessity, or causal mediation. Orthogonal target-engagement, pathway-perturbation, exposure, and independent-batch chemical-validation studies are required before clinical translation.

## Figures and Tables

**Table 1:** Primers for qPCR.

Gene	Forward Primer	Reverse Primer
PARP1	GCTTTATCGAGTGGAGTACGC	GGAGGGAGTCCTTGGGAATAC
CCND1	GCGTACCCTGACACCAATCTC	ACTTGAAGTAAGATACGGAGGGC
PFKFB3	CCCAGAGCCGGGTACAGAA	GGGGAGTTGGTCAGCTTCG
AURKB	TTGTCCCAGAGAGTCCTACGG	TTGTCAATAGTGAAAGGCTGCTT
GAPDH	GGAGAGTGTTTCCTCGTCCC	GATGGGCTTCCCGTTGATGA

## Data Availability

De-identified derived data supporting the reported analyses are available from the corresponding author upon reasonable request. Availability of raw whole-slide images, model weights/configuration, analysis code, and complete DARTS source-image records remains subject to confirmation of file preservation, institutional sharing requirements, and repository capacity.

## Supplementary Materials

The following supporting information can be downloaded at: https://www.mdpi.com/article/10.3390/life16091574/s1, Supplementary Figure S1: Patch-level architecture comparison using mouse-grouped training and internal test subsets; Supplementary Figure S2: Representative molecular docking poses of candidate analytical-panel compounds with AKT1 and PI3Kα; Supplementary Figure S3: Original PI3K p110α DARTS results and assay-quality documentation; Supplementary Figure S4: Source-image documentation for the original 4-NQO model-background DARTS cohort; Supplementary Figure S5: Source-image and lane-map documentation for the six additional 4-NQO-exposed, in vivo ZSQGY-treated samples; the chemical-monitoring panel contained 40 entries, whereas the revised computational network/docking panel retained 38 entries after excluding two not-found signals; Table S1: Botanical composition, medicinal parts, and mouse-equivalent doses of ZSQGY; Table S2: QC-RSD values for targeted analytes; Table S3: Sample-level concentration summary for targeted analytes; Table S4: Representative targeted analytes and their analytical evidence status; Table S5: Mouse body weight, general condition, mortality, and interval-based sampling records; Table S6: Patch-level model performance in mouse-grouped training and internal test subsets, with retained run-time labels; Table S7: MCODE cluster analysis information; Table S8: Revised 38-candidate panel accounting and topology; Table S9: Unambiguous historical molecular-docking records for retained candidates at PI3Kα and AKT1; Table S10: Complete exploratory DARTS summary statistics; Table S11: Individual-animal exploratory DARTS data; Table S12: DARTS assay quality-control summary; Table S13: β-actin technical quality-control measurements; Table S14: Mouse-level spatial correlation analysis of IHC pathway and phenotype readouts; Table S15: Supplied lane-integrated signals and matched ex vivo vehicle ratios for the additional cohort; Table S16: Exploratory summary statistics, concentration tests, and complete exact/Holm-adjusted comparisons for the additional cohort; Table S17: Matched vehicle residual signal relative to no-Pronase input. Source-image and lane-map documentation for both cohorts is provided with the corresponding supplementary figures and associated source records.

## Abbreviations

The following abbreviations are used in this manuscript:

EC	Esophageal Cancer
EPLs	Esophageal Precancerous Lesions
ZSQGY	Zishui Qinggan Yin
4-NQO	4-nitroquinoline-1-oxide
WB	Western Blot
IHC	Immunohistochemistry
DCNNs	Deep Convolutional Neural Networks
TCM	Traditional Chinese Medicine

## References

[1] Sung H., Ferlay J., Siegel R.L., Laversanne M., Soerjomataram I., Jemal A., Bray F. (2021). Global Cancer Statistics 2020: GLOBOCAN Estimates of Incidence and Mortality Worldwide for 36 Cancers in 185 Countries. CA Cancer J. Clin..

[2] Gao D., Lu P., Zhang N., Zhao L., Liu J., Yang J., Liu J., Zhao D., Wang J. (2023). Progression of precancerous lesions of esophageal squamous cell carcinomas in a high-risk, rural Chinese population. Cancer Med..

[3] Zhou R., Tang X., Wang Y. (2024). Emerging strategies to investigate the biology of early cancer. Nat. Rev. Cancer.

[4] Domchek S.M., Vonderheide R.H. (2024). Advancing Cancer Interception. Cancer Discov..

[5] Dashwood R.H. (2021). Cancer interception by interceptor molecules: Mechanistic, preclinical and human translational studies with chlorophylls. Genes Environ..

[6] Wu Y., Zhang H., Zhou B., Han S., Zhang Y. (2018). Clinical efficacy of endoscopic submucosal dissection in the treatment of early esophageal cancer and precancerous lesions. J. Cancer Res. Ther..

[7] Chang J., Zhao X., Wang Y., Liu T., Zhong C., Lao Y., Zhang S., Liao H., Bai F., Lin D. (2023). Genomic alterations driving precancerous to cancerous lesions in esophageal cancer development. Cancer Cell.

[8] Zhou C., Li J., Li Q. (2017). CDKN2A methylation in esophageal cancer: A meta-analysis. Oncotarget.

[9] Manning B.D., Toker A. (2017). AKT/PKB signaling: Navigating the network. Cell.

[10] Saxton R.A., Sabatini D.M. (2017). mTOR signaling in growth, metabolism, and disease. Cell.

[11] Luo Q., Du R., Liu W., Huang G., Dong Z., Li X. (2022). PI3K/Akt/mTOR signaling pathway: Role in esophageal squamous cell carcinoma, regulatory mechanisms and opportunities for targeted therapy. Front. Oncol..

[12] Huang R., Dai Q., Yang R., Duan Y., Zhao Q., Haybaeck J., Yang Z. (2022). A review: PI3K/AKT/mTOR signaling pathway and its regulated eukaryotic translation initiation factors may be a potential therapeutic target in esophageal squamous cell carcinoma. Front. Oncol..

[13] Ma L., Yao N., Chen P., Zhuang Z. (2019). TRIM27 promotes the development of esophagus cancer via regulating PTEN/AKT signaling pathway. Cancer Cell Int..

[14] Kim S.-H., Chau G.C., Jang Y.-H., Lee S.I., Pyo S., Um S.H. (2013). Clinicopathologic significance and function of mammalian target of rapamycin activation in esophageal squamous cell carcinoma. Hum. Pathol..

[15] Zhu Z., Yu W., Fu X., Sun M., Wei Q., Li D., Chen H., Xiang J., Li H., Zhang Y. (2015). Phosphorylated AKT1 is associated with poor prognosis in esophageal squamous cell carcinoma. J. Exp. Clin. Cancer Res..

[16] Campanella G., Hanna M.G., Geneslaw L., Miraflor A., Werneck Krauss Silva V., Busam K.J., Brogi E., Reuter V.E., Klimstra D.S., Fuchs T.J. (2019). Clinical-grade computational pathology using weakly supervised deep learning on whole-slide images. Nat. Med..

[17] Lu M.Y., Williamson D.F.K., Chen T.Y., Chen R.J., Barbieri M., Mahmood F. (2021). Data-efficient and weakly supervised computational pathology on whole-slide images. Nat. Biomed. Eng..

[18] Dolezal J.M., Srisuwananukorn A., Karpeyev D., Ramesh S., Kochanny S., Cody B., Mansfield A.S., Rakshit S., Bansal R., Bois M.C. (2022). Uncertainty-informed deep learning models enable high-confidence predictions for digital histopathology. Nat. Commun..

[19] Aziz Z., Washington M.K., Jacobse J., Choksi Y. (2023). A method for scoring 4-nitroquinoline 1-oxide-induced murine esophageal squamous neoplasia. Vet. Pathol..

[20] Liu Z., Su R., Ahsan A., Liu C., Liao X., Tian D., Su M. (2022). Esophageal squamous cancer from 4NQO-induced mice model: CNV alterations. Int. J. Mol. Sci..

[21] Huang M., Li J., Wang Y., Jia L., Guo J., Wu Z., Gao S., Li J., Zhang Y. (2024). Ethanol exposure exacerbates 4-nitroquinoline-1-oxide induced esophageal carcinogenesis and induces invasive carcinoma with muscularis propria infiltration in a mouse model. Toxicol. Appl. Pharmacol..

[22] Percie du Sert N., Hurst V., Ahluwalia A., Alam S., Avey M.T., Baker M., Browne W.J., Clark A., Cuthill I.C., Dirnagl U. (2020). The ARRIVE guidelines 2.0: Updated guidelines for reporting animal research. PLoS Biol..

[23] Bankhead P., Loughrey M.B., Fernández J.A., Dombrowski Y., McArt D.G., Dunne P.D., McQuaid S., Gray R.T., Murray L.J., Coleman H.G. (2017). QuPath: Open-source software for digital pathology image analysis. Sci. Rep..

[24] Macenko M., Niethammer M., Marron J.S., Borland D., Woosley J.T., Guan X., Schmitt C., Thomas N.E. A method for normalizing histology slides for quantitative analysis. Proceedings of the 2009 IEEE International Symposium on Biomedical Imaging.

[25] Selvaraju R.R., Cogswell M., Das A., Vedantam R., Parikh D., Batra D. Grad-CAM: Visual explanations from deep networks via gradient-based localization. Proceedings of the 2017 IEEE International Conference on Computer Vision (ICCV).

[26] Kim S., Chen J., Cheng T., Gindulyte A., He J., He S., Li Q., Shoemaker B.A., Thiessen P.A., Yu B. (2023). PubChem 2023 update. Nucleic Acids Res..

[27] Daina A., Michielin O., Zoete V. (2019). SwissTargetPrediction: Updated data and new features for efficient prediction of protein targets of small molecules. Nucleic Acids Res..

[28] The UniProt Consortium (2023). UniProt: The Universal Protein Knowledgebase in 2023. Nucleic Acids Res..

[29] Safran M., Rosen N., Twik M., BarShir R., Stein T.I., Dahary D., Fishilevich S., Lancet D. (2022). The GeneCards Suite. Practical Guide to Life Science Databases.

[30] Brown G.R., Hem V., Katz K.S., Ovetsky M., Wallin C., Ermolaeva O., Tolstoy I., Tatusova T., Pruitt K.D., Maglott D.R. (2015). Gene: A gene-centered information resource at NCBI. Nucleic Acids Res..

[31] Davis A.P., Wiegers T.C., Johnson R.J., Sciaky D., Wiegers J., Mattingly C.J. (2023). Comparative Toxicogenomics Database (CTD): Update 2023. Nucleic Acids Res..

[32] Szklarczyk D., Gable A.L., Nastou K.C., Lyon D., Kirsch R., Pyysalo S., Doncheva N.T., Legeay M., Fang T., Bork P. (2021). The STRING database in 2021: Customizable protein-protein networks, and functional characterization of user-uploaded gene/measurement sets. Nucleic Acids Res..

[33] The Cancer Genome Atlas Research Network (2017). Integrated genomic characterization of oesophageal carcinoma. Nature.

[34] Ritchie M.E., Phipson B., Wu D., Hu Y., Law C.W., Shi W., Smyth G.K. (2015). limma powers differential expression analyses for RNA-sequencing and microarray studies. Nucleic Acids Res..

[35] Shannon P., Markiel A., Ozier O., Baliga N.S., Wang J.T., Ramage D., Amin N., Schwikowski B., Ideker T. (2003). Cytoscape: A software environment for integrated models of biomolecular interaction networks. Genome Res..

[36] Bader G.D., Hogue C.W.V. (2003). An automated method for finding molecular complexes in large protein interaction networks. BMC Bioinform..

[37] The Gene Ontology Consortium (2019). The Gene Ontology Resource: 20 years and still GOing strong. Nucleic Acids Res..

[38] Kanehisa M., Furumichi M., Sato Y., Ishiguro-Watanabe M., Tanabe M. (2021). KEGG: Integrating viruses and cellular organisms. Nucleic Acids Res..

[39] Kanehisa M., Furumichi M., Sato Y., Kawashima M., Ishiguro-Watanabe M. (2023). KEGG for taxonomy-based analysis of pathways and genomes. Nucleic Acids Res..

[40] Yu G., Wang L.-G., Han Y., He Q.-Y. (2012). clusterProfiler: An R package for comparing biological themes among gene clusters. OMICS J. Integr. Biol..

[41] Hopkins A.L. (2008). Network pharmacology: The next paradigm in drug discovery. Nat. Chem. Biol..

[42] Zhai Y., Liu L., Zhang F., Chen X., Wang H., Zhou J., Chai K., Liu J., Lei H., Lu P. (2025). Network pharmacology: A crucial approach in traditional Chinese medicine research. Chin. Med..

[43] Trott O., Olson A.J. (2010). AutoDock Vina: Improving the speed and accuracy of docking with a new scoring function, efficient optimization, and multithreading. J. Comput. Chem..

[44] Lomenick B., Hao R., Jonai N., Chin R.M., Aghajan M., Warburton S., Wang J., Wu R.P., Gomez F., Loo J.A. (2009). Target identification using drug affinity responsive target stability (DARTS). Proc. Natl. Acad. Sci. USA.

[45] McCarty K.S., Miller L.S., Cox E.B., Konrath J., McCarty K.S. (1986). Use of a monoclonal anti-estrogen receptor antibody in the immunohistochemical evaluation of human tumors. Cancer Res..

[46] Livak K.J., Schmittgen T.D. (2001). Analysis of relative gene expression data using real-time quantitative PCR and the 2^−ΔΔCT^ method. Methods.

[47] Bustin S.A., Benes V., Garson J.A., Hellemans J., Huggett J., Kubista M., Mueller R., Nolan T., Pfaffl M.W., Shipley G.L. (2009). The MIQE guidelines: Minimum information for publication of quantitative real-time PCR experiments. Clin. Chem..

[48] Holm S. (1979). A simple sequentially rejective multiple test procedure. Scand. J. Stat..

[49] Gingras A.-C., Raught B., Gygi S.P., Niedzwiecka A., Miron M., Burley S.K., Polakiewicz R.D., Wysłouch-Cieszyńska A., Aebersold R., Sonenberg N. (2001). Hierarchical phosphorylation of the translation inhibitor 4E-BP1. Genes Dev..

[50] Maehama T., Dixon J.E. (1998). The tumor suppressor, PTEN/MMAC1, dephosphorylates the lipid second messenger, phosphatidylinositol 3,4,5-trisphosphate. J. Biol. Chem..

[51] Scholzen T., Gerdes J. (2000). The Ki-67 protein: From the known and the unknown. J. Cell. Physiol..

[52] Fan T.-J., Han L.-H., Cong R.-S., Liang J. (2005). Caspase family proteases and apoptosis. Acta Biochim. Biophys. Sin..

[53] Porter A.G., Jänicke R.U. (1999). Emerging roles of caspase-3 in apoptosis. Cell Death Differ..

[54] Mega S., Miyamoto M., Ebihara Y., Takahashi R., Hase R., Li L., Shichinohe T., Kawarada Y., Uehara H., Kaneko H. (2005). Cyclin D1, E2F1 expression levels are associated with characteristics and prognosis of esophageal squamous cell carcinoma. Dis. Esophagus.

[55] Musgrove E.A., Caldon C.E., Barraclough J., Stone A., Sutherland R.L. (2011). Cyclin D as a therapeutic target in cancer. Nat. Rev. Cancer.

[56] Walker E.H., Pacold M.E., Perisic O., Stephens L., Hawkins P.T., Wymann M.P., Williams R.L. (2000). Structural determinants of phosphoinositide 3-kinase inhibition by wortmannin, LY294002, quercetin, myricetin and staurosporine. Mol. Cell.

[57] Wu W.-I., Voegtli W.C., Sturgis H.L., Dizon F.P., Vigers G.P.A., Brandhuber B.J. (2010). Crystal structure of human AKT1 with an allosteric inhibitor reveals a new mode of kinase inhibition. PLoS ONE.

[58] Sarbassov D.D., Guertin D.A., Ali S.M., Sabatini D.M. (2005). Phosphorylation and regulation of Akt/PKB by the rictor-mTOR complex. Science.

[59] Shen N., Wang T., Gan Q., Liu S., Wang L., Jin B. (2022). Plant flavonoids: Classification, distribution, biosynthesis, and antioxidant activity. Food Chem..

[60] Panche A.N., Diwan A.D., Chandra S.R. (2016). Flavonoids: An overview. J. Nutr. Sci..

[61] Zughaibi T.A., Suhail M., Tarique M., Tabrez S. (2021). Targeting PI3K/Akt/mTOR Pathway by Different Flavonoids: A Cancer Chemopreventive Approach. Int. J. Mol. Sci..

[62] Martínez Molina D., Jafari R., Ignatushchenko M., Seki T., Larsson E.A., Dan C., Sreekumar L., Cao Y., Nordlund P. (2013). Monitoring drug target engagement in cells and tissues using the cellular thermal shift assay. Science.

[63] Fan Y., Mao R., Yang J. (2013). NF-κB and STAT3 signaling pathways collaboratively link inflammation to cancer. Protein Cell.

[64] Ståhl P.L., Salmén F., Vickovic S., Lundmark A., Navarro J.F., Magnusson J., Giacomello S., Asp M., Westholm J.O., Huss M. (2016). Visualization and analysis of gene expression in tissue sections by spatial transcriptomics. Science.

[65] Goltsev Y., Samusik N., Kennedy-Darling J., Bhate S., Hale M., Vazquez G., Black S., Nolan G.P. (2018). Deep profiling of mouse splenic architecture with CODEX multiplexed imaging. Cell.

[66] Chen L., Zhu S., Liu T., Zhao X., Xiang T., Hu X., Wu C., Lin D. (2023). Aberrant epithelial cell interaction promotes esophageal squamous-cell carcinoma development and progression. Signal Transduct. Target. Ther..

