# Supplementary material for: Targeted Chemical Monitoring and Integrated Preclinical Evaluation of Zishui Qinggan Yin in 4-NQO-Induced Esophageal Precancer: Histopathological and PI3K/AKT/mTOR-Associated Changes
Source: Life (Basel). 2026 Sep 21;16(9):1574. doi: 10.3390/life16091574 (PMC13608247; doi:10.3390/life16091574)
Supplement: Supplementary file 1 [file life-16-01574-s001.zip › Approval of Laboratory Animal Ethical and Welfare.pdf]

河北医科大学 HEBEI MEDICAL UNIVERSITY

Laboratory Animal Ethical and Welfare Committee
Hebei Medical University

实验动物福利伦理审查同意书
Approval of Laboratory Animal Ethical and Welfare

编号 Approval No.	IACUC-Hebmu- 2023021

本《动物实验方案》经过河北医科大学实验动物福利伦理委员会审核，符合动物保护、动物福利和伦理原则，符合国家实验动物福利伦理的相关规定。

The protocol of animal experiment listed below was reviewed and approved by the Laboratory Animal Ethical and Welfare Committee of Hebei Medical University.

课题名称 Protocol Title	滋水清肝饮减轻4-NQO诱导的小鼠食管癌前病变进展:深度学习数字病理学与空间PI3K/AKT/mTOR表型分析<br>Zishui Qinggan Yin attenuates 4-NQO-induced esophageal precancer progression in mice: deep-learning digital pathology and spatial PI3K/AKT/mTOR profiling				
申请人 Applicant	吴忠冰<br>Zhong Bing Wu	职称/学位 Title/Degree	博士<br>PhD	邮箱 Email	19001631@hebmu.edu.cn
课题负责人 Principle Investigator (PI)	李晶<br>Jing Li	职称/学位 Title/Degree	教授/博士<br>Professor/PhD	邮箱 Email	lijing@hebmu.edu.cn
院系(部门) Department	中西医结合学院<br>College of Integrated Chinese and Western			申请日期 Application date	2023.4.4
动物种系 Species or Strains	C57BL/6J			动物数量 Quantity	63
计划执行时间 Period of Protocol	2023.5.1	实验动物使用许可证号 Number of Animal use permit		SYXK(冀) 2020-002	
审查意见 Results of inspection	☐ 符合动物福利伦理要求，可以进行实验 Agree<br>☐ 调整方案后，可以进行实验 Agree after modify				
兽医师 Chief Veterinary Officer	[signature]	日期 Date	2023.4.26.		

河北医科大学实验动物福利伦理委员会
Laboratory Animal Ethical and Welfare Committee of Hebei Medical Univeristy
主任委员（或授权人）：[signature]
日 期：2023.6.1

地址: 河北 石家庄市 中山东路 361 号 河北医科大学 邮编：050017
Add: No. 361 East Road, Shijiazhuang, Hebei Province, P. R. China. 050017
