# Supplementary material for: Targeted Chemical Monitoring and Integrated Preclinical Evaluation of Zishui Qinggan Yin in 4-NQO-Induced Esophageal Precancer: Histopathological and PI3K/AKT/mTOR-Associated Changes
Source: Life (Basel). 2026 Sep 21;16(9):1574. doi: 10.3390/life16091574 (PMC13608247; doi:10.3390/life16091574)
Supplement: Supplementary file 1 [file life-16-01574-s001.zip › Table S1.docx]

**Table S1. Botanical composition, medicinal parts, and mouse-equivalent doses of ZSQGY.**

| **Chinese/Pinyin name** | **Pharmaceutical name** | **Botanical source** | **Family** | **Medicinal part** | **Dose (g/kg/day)** |
| --- | --- | --- | --- | --- | --- |
| 白芍 / Bai Shao | Paeoniae Radix Alba | *Paeonia lactiflora Pall.* | Paeoniaceae | Root | 0.43 |
| 柴胡 / Chai Hu | Bupleuri Radix | *Bupleurum chinense DC. or Bupleurum scorzonerifolium Willd.* | Apiaceae | Root | 0.25 |
| 牡丹皮 / Dan Pi | Moutan Cortex | *Paeonia suffruticosa Andrews* | Paeoniaceae | Root bark | 0.334 |
| 当归 / Dang Gui | Angelicae Sinensis Radix | *Angelica sinensis (Oliv.) Diels* | Apiaceae | Root | 1.334 |
| 茯苓 / Fu Ling | Poria | *Wolfiporia cocos (F.A. Wolf) Ryvarden & Gilb.* | Polyporaceae | Sclerotium | 0.08 |
| 山栀子 / Shan Zhi Zi | Gardeniae Fructus | *Gardenia jasminoides J. Ellis* | Rubiaceae | Fruit | 0.10 |
| 山药 / Shan Yao | Dioscoreae Rhizoma | *Dioscorea polystachya Turcz.* | Dioscoreaceae | Rhizome | 0.75 |
| 山茱萸 / Shan Zhu Yu | Corni Fructus | *Cornus officinalis Siebold & Zucc.* | Cornaceae | Fruit | 0.30 |
| 熟地黄 / Shu Di | Rehmanniae Radix Praeparata | *Rehmannia glutinosa (Gaertn.) DC.* | Orobanchaceae | Prepared root tuber | 2.31 |
| 酸枣仁 / Suan Zao Ren | Ziziphi Spinosae Semen | *Ziziphus jujuba Mill. var. spinosa (Bunge) Hu ex H.F. Chow* | Rhamnaceae | Seed | 0.50 |
| 泽泻 / Ze Xie | Alismatis Rhizoma | *Alisma orientale (Sam.) Juzep.* | Alismataceae | Rhizome | 0.25 |
