# Supplementary material for: Targeted Chemical Monitoring and Integrated Preclinical Evaluation of Zishui Qinggan Yin in 4-NQO-Induced Esophageal Precancer: Histopathological and PI3K/AKT/mTOR-Associated Changes
Source: Life (Basel). 2026 Sep 21;16(9):1574. doi: 10.3390/life16091574 (PMC13608247; doi:10.3390/life16091574)
Supplement: Supplementary file 1 [file life-16-01574-s001.zip › Table S2.docx]

**Table S2.** QC-RSD values for targeted analytes.

QC-RSD values were calculated from three QC injections. The QC-RSD range was 0.208%-5.774%, and all analytes were below the 30% threshold. QC SD is presented using the same population-SD convention as the QC-RSD calculation.

| **Analyte** | **QC mean (ng/mL)** | **QC SD (ng/mL)** | **QC RSD (%)** |
| --- | --- | --- | --- |
| Gallic acid | 2578.51 | 87.515 | 3.394 |
| 3,4-Dihydroxybenzoic acid | 2184.97 | 68.438 | 3.132 |
| Protocatechualdehyde | 1937.11 | 25.985 | 1.341 |
| 4-Hydroxybenzoic acid | 1679.81 | 5.993 | 0.357 |
| Phthalic acid | 254.45 | 2.859 | 1.123 |
| Catechin | 198.10 | 2.515 | 1.270 |
| Vanillic acid | 1998.41 | 5.178 | 0.259 |
| Caffeic acid | 1984.77 | 41.499 | 2.091 |
| Syringic acid | 2030.30 | 88.590 | 4.363 |
| Epicatechin | 198.94 | 2.666 | 1.340 |
| Dihydromyricetin | 105.22 | 2.419 | 2.299 |
| Vanillin | 1666.13 | 19.690 | 1.182 |
| p-Hydroxycinnamic Acid | 1922.38 | 5.877 | 0.306 |
| Syringaldehyde | 1717.58 | 6.088 | 0.354 |
| Rutin | 178.79 | 6.912 | 3.866 |
| Vitexin | 181.63 | 2.717 | 1.496 |
| Trans-Ferulic acid | 3836.52 | 15.000 | 0.391 |
| Salicylic acid | 219.69 | 5.045 | 2.297 |
| Sinapic Acid | 1897.26 | 9.753 | 0.514 |
| Quercetin 3-β-D-glucoside | 174.13 | 1.058 | 0.607 |
| Luteoloside | 192.34 | 2.194 | 1.141 |
| (+)-Dihydroquercetin | 171.55 | 1.032 | 0.602 |
| Genistin | 156.87 | 0.987 | 0.629 |
| Benzoic acid | 1670.13 | 23.092 | 1.383 |
| Kaempferol-3-O-glucoside | 193.39 | 3.678 | 1.902 |
| (+)-Dihydrokaempferol | 186.41 | 1.480 | 0.794 |
| Resveratrol | 191.24 | 2.019 | 1.056 |
| Daidzein | 151.10 | 3.488 | 2.308 |
| Luteolin | 156.72 | 0.328 | 0.209 |
| Quercetin | 227.50 | 4.624 | 2.033 |
| Hydrocinnamic acid | 1700.23 | 3.542 | 0.208 |
| Trans-Cinnamic acid | 1733.35 | 4.021 | 0.232 |
| Naringenin Chalcone | 213.69 | 12.338 | 5.774 |
| Phloretin | 173.08 | 3.144 | 1.817 |
| Apigenin | 159.90 | 2.423 | 1.515 |
| Naringenin | 173.39 | 2.426 | 1.399 |
| Kaempferol | 181.76 | 2.690 | 1.480 |
| Isorhamnetin | 140.96 | 3.488 | 2.474 |
| Isoliquiritigenin | 156.62 | 5.959 | 3.805 |
| Gossypol | 416.77 | 16.498 | 3.959 |
