# Supplementary material for: Targeted Chemical Monitoring and Integrated Preclinical Evaluation of Zishui Qinggan Yin in 4-NQO-Induced Esophageal Precancer: Histopathological and PI3K/AKT/mTOR-Associated Changes
Source: Life (Basel). 2026 Sep 21;16(9):1574. doi: 10.3390/life16091574 (PMC13608247; doi:10.3390/life16091574)
Supplement: Supplementary file 1 [file life-16-01574-s001.zip › Table S3.docx]

**Table S3.** Sample-level concentration summary for targeted analytes.

Values summarize the three ZSQGY sample measurements. RSD was not calculated for analytes with a mean concentration of 0 and is shown as NA.

| **Analyte** | **Mean (ng/mL)** | **SD (ng/mL)** | **RSD (%)** |
| --- | --- | --- | --- |
| Gallic acid | 112,672.57 | 733.86 | 0.651 |
| 3,4-Dihydroxybenzoic acid | 1994.75 | 4.826 | 0.242 |
| Protocatechualdehyde | 683.95 | 9.703 | 1.419 |
| 4-Hydroxybenzoic acid | 1802.69 | 14.503 | 0.805 |
| Phthalic acid | 4836.91 | 35.651 | 0.737 |
| Catechin | 14,720.06 | 127.80 | 0.868 |
| Vanillic acid | 2039.49 | 26.282 | 1.289 |
| Caffeic acid | 3340.45 | 42.985 | 1.287 |
| Syringic acid | 920.82 | 15.160 | 1.646 |
| Epicatechin | 1864.42 | 22.083 | 1.184 |
| Dihydromyricetin | 41.287 | 1.224 | 2.965 |
| Vanillin | 736.21 | 1.815 | 0.247 |
| p-Hydroxycinnamic Acid | 1052.01 | 12.678 | 1.205 |
| Syringaldehyde | 251.39 | 2.056 | 0.818 |
| Rutin | 9314.47 | 51.502 | 0.553 |
| Vitexin | 102.71 | 1.688 | 1.643 |
| Trans-Ferulic acid | 9064.49 | 55.741 | 0.615 |
| Salicylic acid | 718.75 | 9.659 | 1.344 |
| Sinapic Acid | 1948.73 | 2.261 | 0.116 |
| Quercetin 3-β-D-glucoside | 1717.79 | 18.753 | 1.092 |
| Luteoloside | 31.100 | 0.159 | 0.510 |
| (+)-Dihydroquercetin | 49.867 | 0.821 | 1.646 |
| Genistin | 57.613 | 0.780 | 1.354 |
| Benzoic acid | 16,408.82 | 85.459 | 0.521 |
| Kaempferol-3-O-glucoside | 277.00 | 3.233 | 1.167 |
| (+)-Dihydrokaempferol | 54.820 | 1.535 | 2.799 |
| Resveratrol | 11.053 | 0.388 | 3.510 |
| Daidzein | 3.767 | 0.061 | 1.622 |
| Luteolin | 9.033 | 0.175 | 1.934 |
| Quercetin | 238.08 | 1.912 | 0.803 |
| Hydrocinnamic acid | 15.667 | 0.070 | 0.448 |
| Trans-Cinnamic acid | 194.09 | 2.257 | 1.163 |
| Naringenin Chalcone | 0.000 | 0.000 | NA |
| Phloretin | 10.707 | 0.223 | 2.083 |
| Apigenin | 3.613 | 0.012 | 0.320 |
| Naringenin | 46.407 | 0.234 | 0.503 |
| Kaempferol | 88.007 | 0.691 | 0.785 |
| Isorhamnetin | 123.88 | 1.161 | 0.937 |
| Isoliquiritigenin | 0.000 | 0.000 | NA |
| Gossypol | 539.27 | 2.813 | 0.522 |
