# Supplementary material for: Targeted Chemical Monitoring and Integrated Preclinical Evaluation of Zishui Qinggan Yin in 4-NQO-Induced Esophageal Precancer: Histopathological and PI3K/AKT/mTOR-Associated Changes
Source: Life (Basel). 2026 Sep 21;16(9):1574. doi: 10.3390/life16091574 (PMC13608247; doi:10.3390/life16091574)
Supplement: Supplementary file 1 [file life-16-01574-s001.zip › Table S4.docx]

**Table S4.** Representative targeted analytes and their analytical evidence status.

Representative entries were selected to show the range of analytical support in the predefined 40-analyte panel; they are not ranked by abundance or pharmacological importance.

| **Analyte** | **Chemical class** | **Analytical status** | **Evidence available** | **Permitted interpretation** |
| --- | --- | --- | --- | --- |
| Rutin | Flavonol glycoside | PROVISIONAL_IN_RANGE | Targeted TraceFinder match based on expected accurate mass and retention-time alignment; complete sample-to-standard MS/MS documentation was not available in the supplied files. | May support limited formulation traceability; does not establish systemic exposure, activity, or causal contribution. |
| Trans-Ferulic acid | Phenolic acid | PROVISIONAL_IN_RANGE | Targeted TraceFinder match based on expected accurate mass and retention-time alignment; complete sample-to-standard MS/MS documentation was not available in the supplied files. | May support limited formulation traceability; does not establish systemic exposure, activity, or causal contribution. |
| Quercetin | Flavonol | PROVISIONAL_IN_RANGE | Targeted TraceFinder match based on expected accurate mass and retention-time alignment; complete sample-to-standard MS/MS documentation was not available in the supplied files. | May support limited formulation traceability; does not establish systemic exposure, activity, or causal contribution. |
| Naringenin | Flavanone | PROVISIONAL_IN_RANGE | Targeted TraceFinder match based on expected accurate mass and retention-time alignment; complete sample-to-standard MS/MS documentation was not available in the supplied files. | May support limited formulation traceability; does not establish systemic exposure, activity, or causal contribution. |
| Kaempferol | Flavonol | PROVISIONAL_IN_RANGE | Targeted TraceFinder match based on expected accurate mass and retention-time alignment; complete sample-to-standard MS/MS documentation was not available in the supplied files. | May support limited formulation traceability; does not establish systemic exposure, activity, or causal contribution. |
| Isorhamnetin | O-methylated flavonol | PROVISIONAL_IN_RANGE | Targeted TraceFinder match based on expected accurate mass and retention-time alignment; complete sample-to-standard MS/MS documentation was not available in the supplied files. | May support limited formulation traceability; does not establish systemic exposure, activity, or causal contribution. |
| Gallic acid | Phenolic acid | ABOVE_PROVISIONAL_RANGE | Targeted response was reported, but it exceeded the vendor's provisional working range; validated dilution/reanalysis documentation was unavailable. | Detected response only; do not report a definitive content value without validated dilution and repeat analysis. |
| Catechin | Flavan-3-ol | ABOVE_PROVISIONAL_RANGE | Targeted response was reported, but it exceeded the vendor's provisional working range; validated dilution/reanalysis documentation was unavailable. | Detected response only; do not report a definitive content value without validated dilution and repeat analysis. |
| Benzoic acid | Aromatic carboxylic acid | ABOVE_PROVISIONAL_RANGE | Targeted response was reported, but it exceeded the vendor's provisional working range; validated dilution/reanalysis documentation was unavailable. | Detected response only; do not report a definitive content value without validated dilution and repeat analysis. |
| Luteolin | Flavone | BELOW_PROVISIONAL_RANGE | A targeted response was reported below the vendor's provisional working range; analyte-specific LLOQ documentation was unavailable. | Detected response only; do not interpret the numerical output as a valid quantitative content. |
| Apigenin | Flavone | BELOW_PROVISIONAL_RANGE | A targeted response was reported below the vendor's provisional working range; analyte-specific LLOQ documentation was unavailable. | Detected response only; do not interpret the numerical output as a valid quantitative content. |
| Naringenin Chalcone | Chalcone | REPORTED_NOT_FOUND / ND_REVIEW | No reportable sample signal was recorded in the supplied summary; raw peak, LOD, and integration-rule documentation was unavailable. | Do not describe as a detected constituent. If retained in network analysis, label it explicitly as a predefined computational input. |
| Isoliquiritigenin | Chalcone | REPORTED_NOT_FOUND / ND_REVIEW | No reportable sample signal was recorded in the supplied summary; raw peak, LOD, and integration-rule documentation was unavailable. | Do not describe as a detected constituent. If retained in network analysis, label it explicitly as a predefined computational input. |

**Note:** Analytical status refers only to the vendor-reported targeted analysis of the administered formulation batch. Detection or annotation does not demonstrate systemic exposure, target engagement, or biological activity. Authentic-standard and complete sample-to-standard MS/MS evidence was not available for every entry.
