# Supplementary material for: Targeted Chemical Monitoring and Integrated Preclinical Evaluation of Zishui Qinggan Yin in 4-NQO-Induced Esophageal Precancer: Histopathological and PI3K/AKT/mTOR-Associated Changes
Source: Life (Basel). 2026 Sep 21;16(9):1574. doi: 10.3390/life16091574 (PMC13608247; doi:10.3390/life16091574)
Supplement: Supplementary file 1 [file life-16-01574-s001.zip › Table S5.docx]

Mouse body weight, general condition, mortality, and interval-based sampling records

Table S5. Cage-side general condition, mortality, and interval-based sampling accounting

| **Group** | **Fur condition** | **Activity / feeding / defecation** | **Body-weight pattern** | **Mortality note** |
| --- | --- | --- | --- | --- |
| Control | Dense, glossy, and normal fur throughout the experiment | Normal activity and responsiveness; stable food intake | Week 28: 23.87 ± 1.61 g | No death was noted in the source table |
| 4-NQO model | Initially normal, followed by progressively dull, sparse fur and hair loss | Reduced activity, lethargy/somnolence, constipation, impaired feeding, and late-phase wasting | Week 24: 17.80 ± 2.64 g; week 28: 14.85 ± 1.69 g | Deaths: weeks 8, 18, 23, 25, and 27. Immediate tissue collection; included in the corresponding interval totals |
| ZSQGY-treated | Hair loss occurred after 4-NQO exposure and was most pronounced around weeks 16-17; gradual recovery from approximately week 18 | Improved activity and responsiveness after intervention | Week 24: 21.30 ± 0.97 g; week 28: 21.27 ± 0.55 g | Deaths: weeks 13 and 23. Immediate tissue collection; included in the corresponding interval totals |

# Weekly body weight by group

| **Week** | Control records (n) | **Control mean ± SD (g)** | 4-NQO records (n) | **4-NQO mean ± SD (g)** | ZSQGY records (n) | **ZSQGY mean ± SD (g)** | Event and sampling-accounting note |
| --- | --- | --- | --- | --- | --- | --- | --- |
| 0 | 19 | 14.41 ± 1.12 | 22 | 14.56 ± 1.09 | 22 | 14.47 ± 0.95 | Arrival at animal facility |
| 1 | 19 | 15.48 ± 1.53 | 22 | 17.03 ± 0.97 | 22 | 16.81 ± 0.78 | Start of 4-NQO administration |
| 2 | 19 | 14.22 ± 0.91 | 22 | 15.51 ± 1.93 | 22 | 15.35 ± 1.47 |  |
| 3 | 19 | 17.28 ± 1.72 | 22 | 18.36 ± 0.75 | 22 | 17.98 ± 0.75 |  |
| 4 | 19 | 18.32 ± 1.12 | 22 | 17.57 ± 0.71 | 22 | 17.60 ± 0.78 | 2023-03-25 |
| 5 | 19 | 19.06 ± 1.06 | 22 | 18.33 ± 0.79 | 22 | 18.79 ± 0.79 |  |
| 6 | 19 | 19.14 ± 0.93 | 22 | 19.22 ± 0.92 | 22 | 19.03 ± 0.92 |  |
| 7 | 19 | 19.84 ± 0.98 | 22 | 19.70 ± 1.08 | 22 | 19.39 ± 0.88 |  |
| 8 | 19 | 19.75 ± 1.24 | 22 | 19.97 ± 0.95 | 22 | 19.97 ± 0.75 | Death: 4-NQO (n=1); immediate collection |
| 9 | 19 | 20.02 ± 0.90 | 22 | 20.19 ± 0.86 | 22 | 19.27 ± 0.75 |  |
| 10 | 19 | 17.11 ± 2.15 | 22 | 20.40 ± 1.20 | 22 | 20.05 ± 0.77 |  |
| 11 | 19 | 17.61 ± 0.64 | 22 | 19.85 ± 1.15 | 22 | 19.87 ± 0.69 |  |
| 12 | 19 | 18.01 ± 1.90 | 22 | 20.47 ± 0.92 | 22 | 20.09 ± 0.85 | Interval end; target total completed |
| 13 | 19 | 20.59 ± 1.25 | 21 | 20.15 ± 1.03 | 21 | 20.57 ± 1.07 | Death: ZSQGY (n=1); immediate collection; 4-NQO stopped |
| 14 | 19 | 19.22 ± 1.21 | 21 | 19.78 ± 1.10 | 21 | 20.61 ± 0.89 |  |
| 15 | 19 | 19.53 ± 1.26 | 21 | 20.60 ± 0.99 | 20 | 21.26 ± 1.01 |  |
| 16 | 19 | 20.63 ± 1.80 | 21 | 20.80 ± 1.07 | 17 | 20.68 ± 2.24 | Interval end; target totals C/M/Z = 4/5/5 |
| 17 | 15 | 21.93 ± 1.72 | 16 | 20.35 ± 1.16 | 16 | 19.88 ± 0.94 |  |
| 18 | 14 | 21.59 ± 1.48 | 16 | 20.18 ± 0.77 | 16 | 20.60 ± 0.97 | Death: 4-NQO (n=1); immediate collection |
| 19 | 14 | 21.89 ± 1.46 | 15 | 19.93 ± 0.94 | 16 | 20.71 ± 0.90 |  |
| 20 | 14 | 21.83 ± 1.08 | 15 | 20.89 ± 0.87 | 16 | 20.84 ± 0.94 | Interval end; target totals C/M/Z = 4/5/5 |
| 21 | 10 | 22.24 ± 1.07 | 11 | 19.96 ± 1.03 | 11 | 21.24 ± 0.62 |  |
| 22 | 10 | 21.56 ± 1.26 | 11 | 19.15 ± 1.74 | 11 | 21.49 ± 1.17 |  |
| 23 | 10 | 22.82 ± 0.99 | 10 | 19.19 ± 1.58 | 10 | 21.08 ± 0.60 | Deaths: 4-NQO (n=1), ZSQGY (n=1); immediate collection |
| 24 | 10 | 22.78 ± 0.75 | 10 | 17.80 ± 2.64 | 10 | 21.30 ± 0.97 | Interval end; target totals C/M/Z = 4/5/5 |
| 25 | 6 | 22.97 ± 1.49 | 5 | 18.08 ± 2.26 | 6 | 21.17 ± 0.64 | Death: 4-NQO (n=1); immediate collection |
| 26 | 6 | 23.00 ± 1.26 | 5 | 17.30 ± 2.11 | 6 | 21.47 ± 1.03 |  |
| 27 | 6 | 22.47 ± 1.13 | 5 | 15.54 ± 2.55 | 6 | 21.17 ± 0.85 | Death: 4-NQO (n=1); immediate collection |
| 28 | 6 | 23.87 ± 1.61 | 6 | 14.85 ± 1.69 | 6 | 21.27 ± 0.55 | Scheduled endpoint; remaining mice euthanized as planned |

# Interval-based mortality and sampling accounting

| **Interval ending (week)** | **Group** | **Prespecified tissue total (n)** | **Death-associated immediate collection (n)** | **Scheduled euthanasia at interval end (n)** |
| --- | --- | --- | --- | --- |
| 12 | Control | 1 | 0 | 1 |
| 12 | 4-NQO | 1 | 1 (week 8) | 0 |
| 12 | ZSQGY | 1 | 0 | 1 |
| 16 | Control | 4 | 0 | 4 |
| 16 | 4-NQO | 5 | 0 | 5 |
| 16 | ZSQGY | 5 | 1 (week 13) | 4 |
| 20 | Control | 4 | 0 | 4 |
| 20 | 4-NQO | 5 | 1 (week 18) | 4 |
| 20 | ZSQGY | 5 | 0 | 5 |
| 24 | Control | 4 | 0 | 4 |
| 24 | 4-NQO | 5 | 1 (week 23) | 4 |
| 24 | ZSQGY | 5 | 1 (week 23) | 4 |
| 28 | Control | 6 | 0 | 6 |
| 28 | 4-NQO | 6 | 2 (weeks 25 and 27) | 4 |
| 28 | ZSQGY | 6 | 0 | 6 |
| **Total** | **Control** | **19** | **0** | **19** |
| **Total** | **4-NQO** | **22** | **5** | **17** |
| **Total** | **ZSQGY** | **22** | **2** | **20** |

Accounting note: Prespecified numbers refer to total mice contributing tissue within each sampling interval. Every spontaneous death was followed by immediate tissue collection and preservation and was counted within the relevant interval total; scheduled euthanasia at the end of the interval was reduced accordingly. Deaths were therefore not added to the planned sampling numbers. Weekly records (n) in the body-weight table reproduce the number of observations represented in the source summary and are not end-of-week survivor counts. Weights from mice that died were not carried forward; the week-28 body-weight summary represents scheduled endpoint measurements.
