# Supplementary material for: Targeted Chemical Monitoring and Integrated Preclinical Evaluation of Zishui Qinggan Yin in 4-NQO-Induced Esophageal Precancer: Histopathological and PI3K/AKT/mTOR-Associated Changes
Source: Life (Basel). 2026 Sep 21;16(9):1574. doi: 10.3390/life16091574 (PMC13608247; doi:10.3390/life16091574)
Supplement: Supplementary file 1 [file life-16-01574-s001.zip › Table S6.docx]

Table S6. Patch-level model performance in mouse-grouped training and internal test subsets, with retained run-time labels.

| **ModelName** | **Acc** | **AUC** | **95% CI** | **Sensitivity** | **Specificity** | **PPV** | **NPV** | **Class** | **Cohort** |
| --- | --- | --- | --- | --- | --- | --- | --- | --- | --- |
| densenet121 | 0.896 | 0.968 | 0.9652-0.9714 | 0.922 | 0.891 | 0.627 | 0.983 | Moderate Dysplasia | train |
| densenet121 | 0.817 | 0.928 | 0.9232-0.9326 | 0.894 | 0.792 | 0.582 | 0.958 | Mild Dysplasia | train |
| densenet121 | 0.880 | 0.959 | 0.9551-0.9627 | 0.900 | 0.877 | 0.587 | 0.978 | Severe Dysplasia | train |
| densenet121 | 0.911 | 0.975 | 0.9720-0.9771 | 0.912 | 0.911 | 0.752 | 0.972 | Normal | train |
| densenet121 | 0.833 | 0.927 | 0.9216-0.9321 | 0.860 | 0.827 | 0.519 | 0.965 | Inflammatory | train |
| densenet121 | 0.937 | 0.985 | 0.9795-0.9904 | 0.939 | 0.937 | 0.217 | 0.999 | CarcinomaInSitu | train |
| densenet121 | 0.879 | 0.946 | 0.9396-0.9533 | 0.874 | 0.880 | 0.595 | 0.972 | Moderate Dysplasia | test |
| densenet121 | 0.801 | 0.889 | 0.8800-0.8989 | 0.826 | 0.793 | 0.575 | 0.931 | Mild Dysplasia | test |
| densenet121 | 0.878 | 0.938 | 0.9300-0.9469 | 0.836 | 0.886 | 0.589 | 0.965 | Severe Dysplasia | test |
| densenet121 | 0.911 | 0.955 | 0.9489-0.9615 | 0.846 | 0.929 | 0.772 | 0.955 | Normal | test |
| densenet121 | 0.825 | 0.891 | 0.8810-0.9019 | 0.780 | 0.835 | 0.505 | 0.946 | Inflammatory | test |
| densenet121 | 0.962 | 0.976 | 0.9578-0.9943 | 0.916 | 0.963 | 0.276 | 0.999 | CarcinomaInSitu | test |
| vgg19 | 0.845 | 0.938 | 0.9332-0.9430 | 0.891 | 0.836 | 0.521 | 0.975 | Moderate Dysplasia | train |
| vgg19 | 0.715 | 0.832 | 0.8249-0.8400 | 0.821 | 0.680 | 0.454 | 0.921 | Mild Dysplasia | train |
| vgg19 | 0.814 | 0.912 | 0.9057-0.9179 | 0.839 | 0.809 | 0.461 | 0.962 | Severe Dysplasia | train |
| vgg19 | 0.871 | 0.943 | 0.9389-0.9476 | 0.841 | 0.879 | 0.674 | 0.949 | Normal | train |
| vgg19 | 0.744 | 0.862 | 0.8545-0.8692 | 0.840 | 0.723 | 0.397 | 0.954 | Inflammatory | train |
| vgg19 | 0.937 | 0.968 | 0.9589-0.9776 | 0.879 | 0.938 | 0.210 | 0.998 | CarcinomaInSitu | train |
| vgg19 | 0.863 | 0.946 | 0.9388-0.9524 | 0.885 | 0.859 | 0.560 | 0.974 | Moderate Dysplasia | test |
| vgg19 | 0.701 | 0.836 | 0.8246-0.8470 | 0.852 | 0.650 | 0.453 | 0.928 | Mild Dysplasia | test |
| vgg19 | 0.833 | 0.925 | 0.9170-0.9333 | 0.876 | 0.825 | 0.494 | 0.972 | Severe Dysplasia | test |
| vgg19 | 0.881 | 0.946 | 0.9397-0.9525 | 0.835 | 0.894 | 0.691 | 0.950 | Normal | test |
| vgg19 | 0.711 | 0.868 | 0.8569-0.8787 | 0.899 | 0.670 | 0.371 | 0.968 | Inflammatory | test |
| vgg19 | 0.879 | 0.970 | 0.9556-0.9843 | 0.940 | 0.878 | 0.107 | 0.999 | CarcinomaInSitu | test |
| inception_v3 | 0.881 | 0.945 | 0.9399-0.9493 | 0.860 | 0.886 | 0.600 | 0.969 | Moderate Dysplasia | train |
| inception_v3 | 0.785 | 0.864 | 0.8569-0.8706 | 0.755 | 0.794 | 0.543 | 0.909 | Mild Dysplasia | train |
| inception_v3 | 0.829 | 0.924 | 0.9186-0.9300 | 0.864 | 0.822 | 0.487 | 0.969 | Severe Dysplasia | train |
| inception_v3 | 0.879 | 0.949 | 0.9445-0.9529 | 0.853 | 0.887 | 0.691 | 0.953 | Normal | train |
| inception_v3 | 0.743 | 0.875 | 0.8675-0.8817 | 0.869 | 0.715 | 0.399 | 0.962 | Inflammatory | train |
| inception_v3 | 0.871 | 0.972 | 0.9634-0.9808 | 0.957 | 0.869 | 0.120 | 0.999 | CarcinomaInSitu | train |
| inception_v3 | 0.894 | 0.955 | 0.9495-0.9615 | 0.875 | 0.898 | 0.635 | 0.973 | Moderate Dysplasia | test |
| inception_v3 | 0.742 | 0.857 | 0.8454-0.8678 | 0.815 | 0.717 | 0.494 | 0.920 | Mild Dysplasia | test |
| inception_v3 | 0.853 | 0.934 | 0.9265-0.9423 | 0.864 | 0.850 | 0.530 | 0.970 | Severe Dysplasia | test |
| inception_v3 | 0.879 | 0.946 | 0.9393-0.9537 | 0.867 | 0.882 | 0.677 | 0.959 | Normal | test |
| inception_v3 | 0.793 | 0.883 | 0.8726-0.8933 | 0.801 | 0.791 | 0.454 | 0.949 | Inflammatory | test |
| inception_v3 | 0.915 | 0.976 | 0.9620-0.9909 | 0.940 | 0.915 | 0.147 | 0.999 | CarcinomaInSitu | test |
