# Supplementary material for: Targeted Chemical Monitoring and Integrated Preclinical Evaluation of Zishui Qinggan Yin in 4-NQO-Induced Esophageal Precancer: Histopathological and PI3K/AKT/mTOR-Associated Changes
Source: Life (Basel). 2026 Sep 21;16(9):1574. doi: 10.3390/life16091574 (PMC13608247; doi:10.3390/life16091574)
Supplement: Supplementary file 1 [file life-16-01574-s001.zip › Table S7.docx]

**Table S7. MCODE cluster analysis information.**

| **Cluster** | **Network** | **Nodes** | **Edges** | **Node IDs** |
| --- | --- | --- | --- | --- |
| 1 |  | 45 | 847 | ICAM1, PDGFRB, MMP9, EP300, TERT, RELA, XIAP, MAPK8, MCL1, CDK1, AR, PTGS2, PARP1, MAP2K1, MAPK14, HSP90AA1, GSK3B, MAPK1, CCNA2, CDK2, ALB, CTNNB1, EGFR, ERBB2, CXCR4, IGF1R, STAT3, STAT1, AKT1, KDR, HIF1A, NFE2L2, CCND1, DNMT1, CDK4, TLR4, ESR1, TNF, MMP2, CCNB1, PTK2, BCL2, PPARG, BCL2L1, SRC |
| 2 |  | 23 | 70 | PIK3CA, ABCG2, MMP14, CCNE1, HDAC2, MMP1, NTRK1, CCND2, SERPINE1, PRKCA, AURKA, MMP3, PDGFRA, PIK3CG, CCNA1, MET, ABCB1, HNF4A, WEE1, PLAU, CHEK1, CDK6, KIT |
| 3 |  | 23 | 51 | NOX4, PLG, HDAC3, XDH, PLK1, TLR9, AXL, MPO, TOP2A, IDO1, MMP13, CCNB2, CCNE2, PLAT, FYN, CDK3, CCND3, PIK3CB, ACE, CDC25B, FGFR1, NOS2, PTPN1 |
| 4 |  | 4 | 5 | PON1, TPMT, CYP2C9, CYP1A2 |
| 5 |  | 4 | 5 | PFKFB3, FBP1, PDK1, EGLN1 |
| 6 |  | 17 | 24 | HMGCR, NEK2, ITGB2, CDK5, F3, ELANE, COL18A1, RXRA, DPP4, AURKB, CD38, FEN1, CTSB, SIRT2, CYP1A1, CCR5, PTGS1 |
