# Supplementary material for: Targeted Chemical Monitoring and Integrated Preclinical Evaluation of Zishui Qinggan Yin in 4-NQO-Induced Esophageal Precancer: Histopathological and PI3K/AKT/mTOR-Associated Changes
Source: Life (Basel). 2026 Sep 21;16(9):1574. doi: 10.3390/life16091574 (PMC13608247; doi:10.3390/life16091574)
Supplement: Supplementary file 1 [file life-16-01574-s001.zip › Table S8.docx]

Table S8. Topology records for archived candidates within the revised 38-candidate computational panel.

| **Name** | **Average Shortest Path Length** | **Betweenness Centrality** | **Closeness Centrality** | **Degree** |
| --- | --- | --- | --- | --- |
| Luteolin | 2.346774 | 0.016614 | 0.426117 | 64 |
| Isorhamnetin | 2.362903 | 0.016953 | 0.423208 | 62 |
| Kaempferol | 2.362903 | 0.01505 | 0.423208 | 62 |
| p-Hydroxycinnamic Acid | 2.354839 | 0.035826 | 0.424658 | 62 |
| Quercetin | 2.379032 | 0.015435 | 0.420339 | 61 |
| Apigenin | 2.379032 | 0.01506 | 0.420339 | 60 |
| Naringenin | 2.403226 | 0.026852 | 0.416107 | 56 |
| Sinapic Acid | 2.435484 | 0.024366 | 0.410596 | 52 |
| Trans-Ferulic acid | 2.556452 | 0.010546 | 0.391167 | 38 |
| Dihydromyricetin | 2.556452 | 0.012024 | 0.391167 | 37 |
| Resveratrol | 2.572581 | 0.009507 | 0.388715 | 35 |
| Caffeic acid | 2.58871 | 0.008342 | 0.386293 | 33 |
| Phloretin | 2.685484 | 0.007585 | 0.372372 | 30 |
| Daidzein | 2.629032 | 0.006058 | 0.380368 | 28 |
| Vanillic acid | 2.709677 | 0.003769 | 0.369048 | 21 |
| Syringic acid | 2.725806 | 0.003478 | 0.366864 | 19 |
| Trans-Cinnamic acid | 2.733871 | 0.001664 | 0.365782 | 16 |
| 3,4-Dihydroxybenzoic acid | 2.741935 | 0.001711 | 0.364706 | 15 |
| Gallic acid | 2.75 | 0.001489 | 0.363636 | 14 |
| Salicylic acid | 2.758065 | 0.001585 | 0.362573 | 13 |
| Luteoloside | 2.774194 | 9.97E-04 | 0.360465 | 12 |
| 4-Hydroxybenzoic acid | 2.774194 | 7.79E-04 | 0.360465 | 11 |
| Gossypol | 2.903226 | 0.001042 | 0.344444 | 11 |
| Quercetin 3-β-D-glucoside | 2.782258 | 6.95E-04 | 0.35942 | 11 |
| Kaempferol-3-O-glucoside | 2.790323 | 5.81E-04 | 0.358382 | 10 |
| Rutin | 2.790323 | 4.71E-04 | 0.358382 | 10 |
| Vanillin | 2.806452 | 0.001001 | 0.356322 | 9 |
| Hydrocinnamic acid | 3 | 3.91E-04 | 0.333333 | 7 |
| Genistin | 2.830645 | 2.92E-04 | 0.353276 | 6 |
| Protocatechualdehyde | 2.846774 | 4.55E-04 | 0.351275 | 6 |
| Syringaldehyde | 2.83871 | 4.96E-04 | 0.352273 | 6 |
| Benzoic acid | 2.854839 | 4.53E-05 | 0.350282 | 5 |
| Vitexin | 2.887097 | 3.56E-05 | 0.346369 | 2 |
| Phthalic acid | 2.895161 | 0 | 0.345404 | 1 |
