# Supplementary material for: Targeted Chemical Monitoring and Integrated Preclinical Evaluation of Zishui Qinggan Yin in 4-NQO-Induced Esophageal Precancer: Histopathological and PI3K/AKT/mTOR-Associated Changes
Source: Life (Basel). 2026 Sep 21;16(9):1574. doi: 10.3390/life16091574 (PMC13608247; doi:10.3390/life16091574)
Supplement: Supplementary file 1 [file life-16-01574-s001.zip › Table S9.docx]

Table S9. Unambiguous historical molecular-docking records for retained candidates at PI3Kalpha and AKT1.

|  | **Binding Energy/(kcal/mol)** | | |
| --- | --- | --- | --- |
| **Active Ingredient of Interest** | **PI3Kα** | **Active Ingredient of Interest** | **AKT1** |
| Luteolin | -8.152 | Quercetin | -8.282 |
| Kaempferol | -7.99 | Isorhamnetin | -7.852 |
| Phloretin | -7.331 | Apigenin | -7.456 |
| Daidzein | -6.82 | Naringenin | -7.354 |
| Gossypol | -6.693 | Rutin | -7.249 |
| Quercetin | -6.399 | Luteolin | -7.241 |
| Gallic acid | -5.911 | Vitexin | -6.913 |
| Caffeic Acid | -5.647 | Resveratrol | -6.729 |
| Isorhamnetin | -5.133 | Dihydromyricetin | -6.496 |
| Sinapic acid | -5.039 | Vanillic Acid | -6.152 |
| 4-Hydroxybenzoic acid | -4.651 | 3,4-Dihydroxybenzoic acid | -6.134 |
|  |  | Syringaldehyde | -6.03 |
| Naringenin | -4.35 |  |  |
| Vitexin | -4.19 | Syringic Acid | -5.968 |
| Apigenin | -4.129 | Kaempferol | -5.892 |
| Dihydromyricetin | -3.814 | 4-Hydroxybenzoic acid | -5.864 |
|  |  | Benzoic acid | -5.778 |
|  |  | Hydrocinnamic acid | -5.733 |
|  |  | Quercetin 3-β-D-glucoside | -5.624 |
|  |  | Protocatechualdehyde | -5.466 |
|  |  | Luteoloside | -5.29 |
|  |  |  |  |
|  |  | Caffeic acid | -4.384 |
|  |  | Gallic acid | -2.977 |
