# Supplementary material for: Targeted Chemical Monitoring and Integrated Preclinical Evaluation of Zishui Qinggan Yin in 4-NQO-Induced Esophageal Precancer: Histopathological and PI3K/AKT/mTOR-Associated Changes
Source: Life (Basel). 2026 Sep 21;16(9):1574. doi: 10.3390/life16091574 (PMC13608247; doi:10.3390/life16091574)
Supplement: Supplementary file 1 [file life-16-01574-s001.zip › Table S10.docx]

**Table S10. Complete exploratory DARTS summary statistics.**

Cohort A (original 4-NQO model-background/vehicle-gavage lysates). Relative protease-resistant target signal normalized to the corresponding same-mouse Vehicle + Pronase condition.

| **Target** | **Treatment** | **Biological n** | **Mean relative protection** | **SD** | **Median** | **Minimum** | **Maximum** | **Bootstrap 95% CI low** | **Bootstrap 95% CI high** | **Replicates above Vehicle** | **Exact two-sided p** | **Holm-adjusted p** |
| --- | --- | --- | --- | --- | --- | --- | --- | --- | --- | --- | --- | --- |
| PI3K p110alpha | Vehicle | 6 | 1 | 0 | 1 | 1 | 1 | 1 | 1 | 0 | 1 | 1 |
| PI3K p110alpha | ZSQGY | 6 | 1.645 | 0.297 | 1.617 | 1.343 | 2.134 | 1.438 | 1.873 | 6 | 0.0312 | 0.1562 |
| PI3K p110alpha | Quercetin | 6 | 1.415 | 0.544 | 1.214 | 0.879 | 2.405 | 1.077 | 1.855 | 5 | 0.0625 | 0.25 |
| PI3K p110alpha | Luteolin | 6 | 1.27 | 0.6 | 1.154 | 0.537 | 2.353 | 0.876 | 1.762 | 5 | 0.375 | 0.75 |
| PI3K p110alpha | MK-2206 | 6 | 0.885 | 0.21 | 0.885 | 0.569 | 1.223 | 0.727 | 1.046 | 1 | 0.25 | 0.75 |
| PI3K p110alpha | LY294002 | 6 | 0.857 | 0.452 | 0.787 | 0.407 | 1.64 | 0.554 | 1.206 | 2 | 0.4375 | 0.75 |
| AKT1-immunoreactive | Vehicle | 6 | 1 | 0 | 1 | 1 | 1 | 1 | 1 | 0 | 1 | 1 |
| AKT1-immunoreactive | ZSQGY | 6 | 2.04 | 1.148 | 2.002 | 0.87 | 4.12 | 1.31 | 2.946 | 5 | 0.0938 | 0.4688 |
| AKT1-immunoreactive | Quercetin | 6 | 2.532 | 2.282 | 2.158 | 0.355 | 6.853 | 1.193 | 4.352 | 5 | 0.0938 | 0.4688 |
| AKT1-immunoreactive | Luteolin | 6 | 2.428 | 2.657 | 1.719 | 0 | 7.507 | 0.862 | 4.616 | 4 | 0.1875 | 0.4688 |
| AKT1-immunoreactive | MK-2206 | 6 | 2.238 | 1.874 | 2.042 | 0 | 5.643 | 1.013 | 3.773 | 5 | 0.0938 | 0.4688 |
| AKT1-immunoreactive | LY294002 | 6 | 0.736 | 0.695 | 0.821 | 0 | 1.869 | 0.274 | 1.231 | 1 | 0.2812 | 0.4688 |
