# Supplementary material for: Targeted Chemical Monitoring and Integrated Preclinical Evaluation of Zishui Qinggan Yin in 4-NQO-Induced Esophageal Precancer: Histopathological and PI3K/AKT/mTOR-Associated Changes
Source: Life (Basel). 2026 Sep 21;16(9):1574. doi: 10.3390/life16091574 (PMC13608247; doi:10.3390/life16091574)
Supplement: Supplementary file 1 [file life-16-01574-s001.zip › Table S11.docx]

**Table S11. Individual-animal exploratory DARTS data.**

Cohort A (original 4-NQO model-background/vehicle-gavage lysates). Local computer paths, ROI coordinates, and script-only fields were removed. Image identifiers are anonymized for submission. Mouse IDs are cohort-specific.

| **Mouse ID** | **Target** | **Lane** | **Lane condition** | **Corrected density** | **Nonnegative density** | **No-Pronase density** | **Vehicle + Pronase density** | **Protected fraction vs No-Pronase** | **Relative protection vs Vehicle** | **Source-export status** |
| --- | --- | --- | --- | --- | --- | --- | --- | --- | --- | --- |
| M01 | AKT1-immunoreactive | 1 | Vehicle, no Pronase | 1,147,556 | 1,147,556 | 1,147,556 | 714,728 | 1 | 1.606 | No |
| M01 | AKT1-immunoreactive | 2 | Vehicle + Pronase | 714,728 | 714,728 | 1,147,556 | 714,728 | 0.623 | 1 | No |
| M01 | AKT1-immunoreactive | 3 | ZSQGY + Pronase | 1,528,286 | 1,528,286 | 1,147,556 | 714,728 | 1.332 | 2.138 | No |
| M01 | AKT1-immunoreactive | 4 | Quercetin + Pronase | 1,311,208 | 1,311,208 | 1,147,556 | 714,728 | 1.143 | 1.835 | No |
| M01 | AKT1-immunoreactive | 5 | Luteolin + Pronase | 690,848 | 690,848 | 1,147,556 | 714,728 | 0.602 | 0.967 | No |
| M01 | AKT1-immunoreactive | 6 | MK-2206 + Pronase | 1,702,854 | 1,702,854 | 1,147,556 | 714,728 | 1.484 | 2.383 | No |
| M01 | AKT1-immunoreactive | 7 | LY294002 + Pronase | 1,335,540 | 1,335,540 | 1,147,556 | 714,728 | 1.164 | 1.869 | No |
| M02 | AKT1-immunoreactive | 1 | Vehicle, no Pronase | 305,046 | 305,046 | 305,046 | 87,506 | 1 | 3.486 | No |
| M02 | AKT1-immunoreactive | 2 | Vehicle + Pronase | 87,506 | 87,506 | 305,046 | 87,506 | 0.287 | 1 | No |
| M02 | AKT1-immunoreactive | 3 | ZSQGY + Pronase | 360,486 | 360,486 | 305,046 | 87,506 | 1.182 | 4.12 | No |
| M02 | AKT1-immunoreactive | 4 | Quercetin + Pronase | 599,636 | 599,636 | 305,046 | 87,506 | 1.966 | 6.853 | No |
| M02 | AKT1-immunoreactive | 5 | Luteolin + Pronase | 656,908 | 656,908 | 305,046 | 87,506 | 2.153 | 7.507 | No |
| M02 | AKT1-immunoreactive | 6 | MK-2206 + Pronase | 493,814 | 493,814 | 305,046 | 87,506 | 1.619 | 5.643 | No |
| M02 | AKT1-immunoreactive | 7 | LY294002 + Pronase | -262,664 | 0 | 305,046 | 87,506 | 0 | 0 | No |
| M03 | AKT1-immunoreactive | 1 | Vehicle, no Pronase | 88,765.5 | 88,765.5 | 88,765.5 | 34,354.5 | 1 | 2.584 | Yes |
| M03 | AKT1-immunoreactive | 2 | Vehicle + Pronase | 34,354.5 | 34,354.5 | 88,765.5 | 34,354.5 | 0.387 | 1 | Yes |
| M03 | AKT1-immunoreactive | 3 | ZSQGY + Pronase | 66,367.5 | 66,367.5 | 88,765.5 | 34,354.5 | 0.748 | 1.932 | Yes |
| M03 | AKT1-immunoreactive | 4 | Quercetin + Pronase | 89,326 | 89,326 | 88,765.5 | 34,354.5 | 1.006 | 2.6 | Yes |
| M03 | AKT1-immunoreactive | 5 | Luteolin + Pronase | 91,291.5 | 91,291.5 | 88,765.5 | 34,354.5 | 1.028 | 2.657 | Yes |
| M03 | AKT1-immunoreactive | 6 | MK-2206 + Pronase | 69,077.5 | 69,077.5 | 88,765.5 | 34,354.5 | 0.778 | 2.011 | Yes |
| M03 | AKT1-immunoreactive | 7 | LY294002 + Pronase | 29,983.5 | 29,983.5 | 88,765.5 | 34,354.5 | 0.338 | 0.873 | Yes |
| M04 | AKT1-immunoreactive | 1 | Vehicle, no Pronase | 2,528,692 | 2,528,692 | 2,528,692 | 715,260 | 1 | 3.535 | No |
| M04 | AKT1-immunoreactive | 2 | Vehicle + Pronase | 715,260 | 715,260 | 2,528,692 | 715,260 | 0.283 | 1 | No |
| M04 | AKT1-immunoreactive | 3 | ZSQGY + Pronase | 622,060 | 622,060 | 2,528,692 | 715,260 | 0.246 | 0.87 | No |
| M04 | AKT1-immunoreactive | 4 | Quercetin + Pronase | 254,052 | 254,052 | 2,528,692 | 715,260 | 0.1 | 0.355 | No |
| M04 | AKT1-immunoreactive | 5 | Luteolin + Pronase | -385,282 | 0 | 2,528,692 | 715,260 | 0 | 0 | No |
| M04 | AKT1-immunoreactive | 6 | MK-2206 + Pronase | -1,237,686 | 0 | 2,528,692 | 715,260 | 0 | 0 | No |
| M04 | AKT1-immunoreactive | 7 | LY294002 + Pronase | -2,545,156 | 0 | 2,528,692 | 715,260 | 0 | 0 | No |
| M05 | AKT1-immunoreactive | 1 | Vehicle, no Pronase | 137,174 | 137,174 | 137,174 | 50,138 | 1 | 2.736 | Yes |
| M05 | AKT1-immunoreactive | 2 | Vehicle + Pronase | 50,138 | 50,138 | 137,174 | 50,138 | 0.366 | 1 | Yes |
| M05 | AKT1-immunoreactive | 3 | ZSQGY + Pronase | 103,858 | 103,858 | 137,174 | 50,138 | 0.757 | 2.071 | Yes |
| M05 | AKT1-immunoreactive | 4 | Quercetin + Pronase | 124,437 | 124,437 | 137,174 | 50,138 | 0.907 | 2.482 | Yes |
| M05 | AKT1-immunoreactive | 5 | Luteolin + Pronase | 109,299 | 109,299 | 137,174 | 50,138 | 0.797 | 2.18 | Yes |
| M05 | AKT1-immunoreactive | 6 | MK-2206 + Pronase | 103,965 | 103,965 | 137,174 | 50,138 | 0.758 | 2.074 | Yes |
| M05 | AKT1-immunoreactive | 7 | LY294002 + Pronase | 38,538 | 38,538 | 137,174 | 50,138 | 0.281 | 0.769 | Yes |
| M06 | AKT1-immunoreactive | 1 | Vehicle, no Pronase | 180,558.5 | 180,558.5 | 180,558.5 | 123,998 | 1 | 1.456 | Yes |
| M06 | AKT1-immunoreactive | 2 | Vehicle + Pronase | 123,998 | 123,998 | 180,558.5 | 123,998 | 0.687 | 1 | Yes |
| M06 | AKT1-immunoreactive | 3 | ZSQGY + Pronase | 137,330 | 137,330 | 180,558.5 | 123,998 | 0.761 | 1.108 | Yes |
| M06 | AKT1-immunoreactive | 4 | Quercetin + Pronase | 132,249 | 132,249 | 180,558.5 | 123,998 | 0.732 | 1.067 | Yes |
| M06 | AKT1-immunoreactive | 5 | Luteolin + Pronase | 156,038 | 156,038 | 180,558.5 | 123,998 | 0.864 | 1.258 | Yes |
| M06 | AKT1-immunoreactive | 6 | MK-2206 + Pronase | 163,141 | 163,141 | 180,558.5 | 123,998 | 0.904 | 1.316 | Yes |
| M06 | AKT1-immunoreactive | 7 | LY294002 + Pronase | 112,536 | 112,536 | 180,558.5 | 123,998 | 0.623 | 0.908 | Yes |
| M01 | PI3K p110alpha | 1 | Vehicle, no Pronase | 2,101,402 | 2,101,402 | 2,101,402 | 869,914 | 1 | 2.416 | No |
| M01 | PI3K p110alpha | 2 | Vehicle + Pronase | 869,914 | 869,914 | 2,101,402 | 869,914 | 0.414 | 1 | No |
| M01 | PI3K p110alpha | 3 | ZSQGY + Pronase | 1,856,702 | 1,856,702 | 2,101,402 | 869,914 | 0.884 | 2.134 | No |
| M01 | PI3K p110alpha | 4 | Quercetin + Pronase | 1,425,052 | 1,425,052 | 2,101,402 | 869,914 | 0.678 | 1.638 | No |
| M01 | PI3K p110alpha | 5 | Luteolin + Pronase | 1,188,384 | 1,188,384 | 2,101,402 | 869,914 | 0.566 | 1.366 | No |
| M01 | PI3K p110alpha | 6 | MK-2206 + Pronase | 714,182 | 714,182 | 2,101,402 | 869,914 | 0.34 | 0.821 | No |
| M01 | PI3K p110alpha | 7 | LY294002 + Pronase | 1,426,294 | 1,426,294 | 2,101,402 | 869,914 | 0.679 | 1.64 | No |
| M02 | PI3K p110alpha | 1 | Vehicle, no Pronase | 2,151,522 | 2,151,522 | 2,151,522 | 1,383,518 | 1 | 1.555 | No |
| M02 | PI3K p110alpha | 2 | Vehicle + Pronase | 1,383,518 | 1,383,518 | 2,151,522 | 1,383,518 | 0.643 | 1 | No |
| M02 | PI3K p110alpha | 3 | ZSQGY + Pronase | 2,229,298 | 2,229,298 | 2,151,522 | 1,383,518 | 1.036 | 1.611 | No |
| M02 | PI3K p110alpha | 4 | Quercetin + Pronase | 1,215,852 | 1,215,852 | 2,151,522 | 1,383,518 | 0.565 | 0.879 | No |
| M02 | PI3K p110alpha | 5 | Luteolin + Pronase | 742,332 | 742,332 | 2,151,522 | 1,383,518 | 0.345 | 0.537 | No |
| M02 | PI3K p110alpha | 6 | MK-2206 + Pronase | 1,282,042 | 1,282,042 | 2,151,522 | 1,383,518 | 0.596 | 0.927 | No |
| M02 | PI3K p110alpha | 7 | LY294002 + Pronase | 1,149,978 | 1,149,978 | 2,151,522 | 1,383,518 | 0.534 | 0.831 | No |
| M03 | PI3K p110alpha | 1 | Vehicle, no Pronase | 47,925.5 | 47,925.5 | 47,925.5 | 24,652 | 1 | 1.944 | Yes |
| M03 | PI3K p110alpha | 2 | Vehicle + Pronase | 24,652 | 24,652 | 47,925.5 | 24,652 | 0.514 | 1 | Yes |
| M03 | PI3K p110alpha | 3 | ZSQGY + Pronase | 44,509 | 44,509 | 47,925.5 | 24,652 | 0.929 | 1.805 | Yes |
| M03 | PI3K p110alpha | 4 | Quercetin + Pronase | 59,296 | 59,296 | 47,925.5 | 24,652 | 1.237 | 2.405 | Yes |
| M03 | PI3K p110alpha | 5 | Luteolin + Pronase | 58,002 | 58,002 | 47,925.5 | 24,652 | 1.21 | 2.353 | Yes |
| M03 | PI3K p110alpha | 6 | MK-2206 + Pronase | 30,143 | 30,143 | 47,925.5 | 24,652 | 0.629 | 1.223 | Yes |
| M03 | PI3K p110alpha | 7 | LY294002 + Pronase | 18,312 | 18,312 | 47,925.5 | 24,652 | 0.382 | 0.743 | Yes |
| M04 | PI3K p110alpha | 1 | Vehicle, no Pronase | 935,910 | 935,910 | 935,910 | 1,317,076 | 1 | 0.711 | No |
| M04 | PI3K p110alpha | 2 | Vehicle + Pronase | 1,317,076 | 1,317,076 | 935,910 | 1,317,076 | 1.407 | 1 | No |
| M04 | PI3K p110alpha | 3 | ZSQGY + Pronase | 2,135,914 | 2,135,914 | 935,910 | 1,317,076 | 2.282 | 1.622 | No |
| M04 | PI3K p110alpha | 4 | Quercetin + Pronase | 1,652,288 | 1,652,288 | 935,910 | 1,317,076 | 1.765 | 1.255 | No |
| M04 | PI3K p110alpha | 5 | Luteolin + Pronase | 1,606,498 | 1,606,498 | 935,910 | 1,317,076 | 1.717 | 1.22 | No |
| M04 | PI3K p110alpha | 6 | MK-2206 + Pronase | 749,050 | 749,050 | 935,910 | 1,317,076 | 0.8 | 0.569 | No |
| M04 | PI3K p110alpha | 7 | LY294002 + Pronase | 612,656 | 612,656 | 935,910 | 1,317,076 | 0.655 | 0.465 | No |
| M05 | PI3K p110alpha | 1 | Vehicle, no Pronase | 181,247 | 181,247 | 181,247 | 148,363 | 1 | 1.222 | Yes |
| M05 | PI3K p110alpha | 2 | Vehicle + Pronase | 148,363 | 148,363 | 181,247 | 148,363 | 0.819 | 1 | Yes |
| M05 | PI3K p110alpha | 3 | ZSQGY + Pronase | 199,225 | 199,225 | 181,247 | 148,363 | 1.099 | 1.343 | Yes |
| M05 | PI3K p110alpha | 4 | Quercetin + Pronase | 168,836 | 168,836 | 181,247 | 148,363 | 0.932 | 1.138 | Yes |
| M05 | PI3K p110alpha | 5 | Luteolin + Pronase | 161,595 | 161,595 | 181,247 | 148,363 | 0.892 | 1.089 | Yes |
| M05 | PI3K p110alpha | 6 | MK-2206 + Pronase | 135,017 | 135,017 | 181,247 | 148,363 | 0.745 | 0.91 | Yes |
| M05 | PI3K p110alpha | 7 | LY294002 + Pronase | 156,719 | 156,719 | 181,247 | 148,363 | 0.865 | 1.056 | Yes |
| M06 | PI3K p110alpha | 1 | Vehicle, no Pronase | 122,799 | 122,799 | 122,799 | 126,499 | 1 | 0.971 | Yes |
| M06 | PI3K p110alpha | 2 | Vehicle + Pronase | 126,499 | 126,499 | 122,799 | 126,499 | 1.03 | 1 | Yes |
| M06 | PI3K p110alpha | 3 | ZSQGY + Pronase | 171,526 | 171,526 | 122,799 | 126,499 | 1.397 | 1.356 | Yes |
| M06 | PI3K p110alpha | 4 | Quercetin + Pronase | 148,544 | 148,544 | 122,799 | 126,499 | 1.21 | 1.174 | Yes |
| M06 | PI3K p110alpha | 5 | Luteolin + Pronase | 133,853 | 133,853 | 122,799 | 126,499 | 1.09 | 1.058 | Yes |
| M06 | PI3K p110alpha | 6 | MK-2206 + Pronase | 108,715 | 108,715 | 122,799 | 126,499 | 0.885 | 0.859 | Yes |
| M06 | PI3K p110alpha | 7 | LY294002 + Pronase | 51,501 | 51,501 | 122,799 | 126,499 | 0.419 | 0.407 | Yes |
