# Supplementary material for: Targeted Chemical Monitoring and Integrated Preclinical Evaluation of Zishui Qinggan Yin in 4-NQO-Induced Esophageal Precancer: Histopathological and PI3K/AKT/mTOR-Associated Changes
Source: Life (Basel). 2026 Sep 21;16(9):1574. doi: 10.3390/life16091574 (PMC13608247; doi:10.3390/life16091574)
Supplement: Supplementary file 1 [file life-16-01574-s001.zip › Table S12.docx]

**Table S12. DARTS assay-quality-control summary.**

Cohort A (original 4-NQO model-background/vehicle-gavage lysates). Pronase-window screening and target-appropriate positive-control results. PI3K p110alpha assay performance is treated as technically inconclusive.

| **Mouse ID** | **Target** | **No-Pronase density** | **Vehicle + Pronase density** | **Protected fraction** | **Window low** | **Window high** |
| --- | --- | --- | --- | --- | --- | --- |
| M01 | AKT1-immunoreactive | 1,147,556 | 714,728 | 0.623 | 0.2 | 0.6 |
| M01 | PI3K p110alpha | 2,101,402 | 869,914 | 0.414 | 0.2 | 0.6 |
| M02 | AKT1-immunoreactive | 305,046 | 87,506 | 0.287 | 0.2 | 0.6 |
| M02 | PI3K p110alpha | 2,151,522 | 1,383,518 | 0.643 | 0.2 | 0.6 |
| M03 | AKT1-immunoreactive | 88,765.5 | 34,354.5 | 0.387 | 0.2 | 0.6 |
| M03 | PI3K p110alpha | 47,925.5 | 24,652 | 0.514 | 0.2 | 0.6 |
| M04 | AKT1-immunoreactive | 2,528,692 | 715,260 | 0.283 | 0.2 | 0.6 |
| M04 | PI3K p110alpha | 935,910 | 1,317,076 | 1.407 | 0.2 | 0.6 |
| M05 | AKT1-immunoreactive | 137,174 | 50,138 | 0.366 | 0.2 | 0.6 |
| M05 | PI3K p110alpha | 181,247 | 148,363 | 0.819 | 0.2 | 0.6 |
| M06 | AKT1-immunoreactive | 180,558.5 | 123,998 | 0.687 | 0.2 | 0.6 |
| M06 | PI3K p110alpha | 122,799 | 126,499 | 1.03 | 0.2 | 0.6 |
| Target-appropriate positive controls |  |  |  |  |  |  |
| Mouse ID | Target | Positive control | Relative protection vs Vehicle |  |  |  |
| M01 | AKT1-immunoreactive | MK-2206 | 2.4 |  |  |  |
| M01 | PI3K p110alpha | LY294002 | 1.6 |  |  |  |
| M02 | AKT1-immunoreactive | MK-2206 | 5.6 |  |  |  |
| M02 | PI3K p110alpha | LY294002 | 0.8 |  |  |  |
| M03 | AKT1-immunoreactive | MK-2206 | 2.0 |  |  |  |
| M03 | PI3K p110alpha | LY294002 | 0.7 |  |  |  |
| M04 | AKT1-immunoreactive | MK-2206 | 0 |  |  |  |
| M04 | PI3K p110alpha | LY294002 | 0.5 |  |  |  |
| M05 | AKT1-immunoreactive | MK-2206 | 2.1 |  |  |  |
| M05 | PI3K p110alpha | LY294002 | 1.1 |  |  |  |
| M06 | AKT1-immunoreactive | MK-2206 | 1.3 |  |  |  |
| M06 | PI3K p110alpha | LY294002 | 0.4 |  |  |  |
