# Supplementary material for: Targeted Chemical Monitoring and Integrated Preclinical Evaluation of Zishui Qinggan Yin in 4-NQO-Induced Esophageal Precancer: Histopathological and PI3K/AKT/mTOR-Associated Changes
Source: Life (Basel). 2026 Sep 21;16(9):1574. doi: 10.3390/life16091574 (PMC13608247; doi:10.3390/life16091574)
Supplement: Supplementary file 1 [file life-16-01574-s001.zip › Table S13.docx]

**Table S13. Beta-actin technical quality-control summary.**

Cohort A (original 4-NQO model-background/vehicle-gavage lysates). Beta-actin was evaluated only as a lane-quality indicator because Pronase can degrade beta-actin; it was not used as the primary DARTS denominator.

| **Mouse ID** | **Number of lanes** | **Mean corrected density** | **SD** | **Coefficient of variation** | **Maximum zero-pixel fraction** |
| --- | --- | --- | --- | --- | --- |
| M01 | 7 | 5,152,862.3 | 1623229.267 | 0.315 |  |
| M02 | 7 | 2,334,103.9 | 581593.445 | 0.249 |  |
| M03 | 7 | 155,218.7 | 21874.562 | 0.141 | 0.678 |
| M04 | 7 | 1,144,618.6 | 1007718.519 | 0.88 |  |
| M05 | 7 | 215,230 | 34084.047 | 0.158 | 0.655 |
| M06 | 7 | 192,383.6 | 34005.777 | 0.177 | 0.559 |
