# Supplementary material for: Targeted Chemical Monitoring and Integrated Preclinical Evaluation of Zishui Qinggan Yin in 4-NQO-Induced Esophageal Precancer: Histopathological and PI3K/AKT/mTOR-Associated Changes
Source: Life (Basel). 2026 Sep 21;16(9):1574. doi: 10.3390/life16091574 (PMC13608247; doi:10.3390/life16091574)
Supplement: Supplementary file 1 [file life-16-01574-s001.zip › Table S14.docx]

**Table S14.** Mouse-level spatial correlation analysis of IHC pathway and phenotype readouts

Correlation analyses were performed using animal-level values after averaging ROI-level measurements within each mouse. Spearman rank correlation was used because histologic grade is an ordered variable and sample size was limited.

| **Variable 1** | **Variable 2** | **Analysis level** | **n** | **Spearman rho** | **P value** | **Significance** | **Direction / interpretation** |
| --- | --- | --- | --- | --- | --- | --- | --- |
| p-AKT H-score | Ki-67 labeling index | Mouse-level means | 18 | 0.676 | 0.002 | ** | Positive association between AKT phosphorylation readout and epithelial proliferation |
| p-mTOR H-score | Ki-67 labeling index | Mouse-level means | 18 | 0.899 | <0.001 | *** | Positive association between mTOR activation readout and epithelial proliferation |
| p-S6K1 H-score | Histologic grade | Mouse-level means | 18 | 0.716 | <0.001 | *** | Positive association between mTORC1 downstream output and lesion grade |
| p-4E-BP1 H-score | Histologic grade | Mouse-level means | 18 | 0.385 | 0.115 | ns | Positive association between mTORC1 downstream output and lesion grade |
| PTEN H-score | Histologic grade | Mouse-level means | 18 | -0.777 | <0.001 | *** | Negative association between PTEN expression and lesion grade |
| Cleaved caspase-3 H-score | Ki-67 labeling index | Mouse-level means | 18 | -0.259 | 0.299 | ns | Inverse association between apoptosis-related signal and epithelial proliferation |

Abbreviations and metrics: p-AKT, p-mTOR, p-S6K1, p-4E-BP1, PTEN, and cleaved caspase-3 were quantified by H-score. Ki-67 was expressed as positive epithelial cells (%).

Histologic grade was encoded from ROI classifications as: Negative = 0, change region = 1, mild dysplasia = 2, moderate dysplasia = 3, severe dysplasia = 4. For the main correlation table, a mouse-level histologic grade score was calculated as the mean of all available ROI grade codes within each mouse.

P values are approximate two-sided P values for Spearman correlation. *P < 0.05, **P < 0.01, ***P < 0.001; ns, not significant.

supporting information. Animal-level histologic grade scores used for correlation analyses

| **Group** | **Animal ID** | **Mean ROI grade** | **Maximum ROI grade** | **ROI records** |
| --- | --- | --- | --- | --- |
| Control | 1 | 0.000 | 0 | 18 |
| Control | 4 | 0.000 | 0 | 15 |
| Control | 7 | 0.000 | 0 | 14 |
| Control | 10 | 0.375 | 3 | 8 |
| Control | 13 | 0.000 | 0 | 7 |
| Control | 16 | 0.000 | 0 | 7 |
| ZSQGY | 2 | 1.205 | 4 | 44 |
| ZSQGY | 5 | 1.421 | 2 | 19 |
| ZSQGY | 8 | 1.158 | 2 | 19 |
| ZSQGY | 11 | 3.000 | 4 | 8 |
| ZSQGY | 14 | 2.286 | 4 | 7 |
| ZSQGY | 17 | 2.571 | 4 | 7 |
| 4-NQO | 3 | 3.042 | 4 | 48 |
| 4-NQO | 6 | 3.050 | 4 | 20 |
| 4-NQO | 9 | 3.000 | 3 | 19 |
| 4-NQO | 12 | 3.000 | 3 | 12 |
| 4-NQO | 15 | 3.000 | 3 | 8 |
| 4-NQO | 18 | 3.000 | 3 | 9 |

This supporting table is included to make the histologic-grade coding used in Table S14 transparent and reproducible from the ROI-level annotations.
