# Supplementary material for: Targeted Chemical Monitoring and Integrated Preclinical Evaluation of Zishui Qinggan Yin in 4-NQO-Induced Esophageal Precancer: Histopathological and PI3K/AKT/mTOR-Associated Changes
Source: Life (Basel). 2026 Sep 21;16(9):1574. doi: 10.3390/life16091574 (PMC13608247; doi:10.3390/life16091574)
Supplement: Supplementary file 1 [file life-16-01574-s001.zip › Table S15.docx]

**DARTS Supplementary Results Table S15**

Table S15. Individual measurements and normalized values for Cohort B

Cohort B comprised six matched sample sets from 4-NQO-exposed mice that had received ZSQGY in vivo. The supplied lane-integrated signals are shown without additional local-background subtraction.

A. Supplied lane-integrated signals (a.u.). No Pronase is the undigested input; all other conditions include Pronase.

| **Sample** | **No Pronase** | **Vehicle** | **Z50** | **Z100** | **Z200** | **Q50** | **L50** |
| --- | --- | --- | --- | --- | --- | --- | --- |
| M01 | 10000 | 4000 | 4600 | 6800 | 7680 | 6000 | 5400 |
| M02 | 12000 | 5040 | 6653 | 10080 | 11088 | 8820 | 7812 |
| M03 | 9000 | 3420 | 4378 | 6327 | 6156 | 5472 | 4959 |
| M04 | 11000 | 4510 | 6359 | 10148 | 10734 | 8118 | 7667 |
| M05 | 10500 | 3990 | 4708 | 6384 | 7780 | 5786 | 5187 |
| M06 | 9500 | 4085 | 3840 | 4494 | 5310 | 4289 | 4494 |

B. Relative protection versus the matched vehicle-plus-Pronase condition.

| **Sample** | **Vehicle** | **Z50** | **Z100** | **Z200** | **Q50** | **L50** |
| --- | --- | --- | --- | --- | --- | --- |
| M01 | 1.0000 | 1.1500 | 1.7000 | 1.9200 | 1.5000 | 1.3500 |
| M02 | 1.0000 | 1.3200 | 2.0000 | 2.2000 | 1.7500 | 1.5500 |
| M03 | 1.0000 | 1.2801 | 1.8500 | 1.8000 | 1.6000 | 1.4500 |
| M04 | 1.0000 | 1.4100 | 2.2501 | 2.3800 | 1.8000 | 1.7000 |
| M05 | 1.0000 | 1.1799 | 1.6000 | 1.9499 | 1.4501 | 1.3000 |
| M06 | 1.0000 | 0.9400 | 1.1001 | 1.2999 | 1.0499 | 1.1001 |

Z50, Z100 and Z200 denote ZSQGY at 50, 100 and 200 microgram/mL; Q50 and L50 denote quercetin and luteolin at 50 micromolar. Each ratio equals the treatment integral divided by the same sample's Vehicle integral. Beta-actin is not the denominator. All six rows are retained.
