# Supplementary material for: Targeted Chemical Monitoring and Integrated Preclinical Evaluation of Zishui Qinggan Yin in 4-NQO-Induced Esophageal Precancer: Histopathological and PI3K/AKT/mTOR-Associated Changes
Source: Life (Basel). 2026 Sep 21;16(9):1574. doi: 10.3390/life16091574 (PMC13608247; doi:10.3390/life16091574)
Supplement: Supplementary file 1 [file life-16-01574-s001.zip › Table S16.docx]

**DARTS Supplementary Results Table S16**

Table S16. Exploratory summary statistics and tests for Cohort B

A. Five treatment-versus-vehicle comparisons. Vehicle is fixed at 1 by normalization.

| **Condition** | **Mean ± SD** | **95% CI** | **>Vehicle** | **Exact P** | **Holm P** |
| --- | --- | --- | --- | --- | --- |
| ZSQGY 50 | 1.213 ± 0.164 | 1.088-1.323 | 5/6 | 0.06250 | 0.15625 |
| ZSQGY 100 | 1.750 ± 0.392 | 1.450-2.017 | 6/6 | 0.03125 | 0.15625 |
| ZSQGY 200 | 1.925 ± 0.371 | 1.642-2.177 | 6/6 | 0.03125 | 0.15625 |
| Quercetin 50 | 1.525 ± 0.270 | 1.317-1.700 | 6/6 | 0.03125 | 0.15625 |
| Luteolin 50 | 1.408 ± 0.208 | 1.258-1.558 | 6/6 | 0.03125 | 0.15625 |

ZSQGY concentrations are in microgram/mL; candidate-compound concentrations are in micromolar.

B. Paired differences between ZSQGY concentrations, expressed in relative-protection units.

| **Comparison** | **Mean difference** | **95% CI** | **Positive pairs** | **Exact P** | **Holm P** |
| --- | --- | --- | --- | --- | --- |
| Z100 - Z50 | 0.537 | 0.358-0.697 | 6/6 | 0.03125 | 0.09375 |
| Z200 - Z50 | 0.712 | 0.542-0.870 | 6/6 | 0.03125 | 0.09375 |
| Z200 - Z100 | 0.175 | 0.075-0.263 | 5/6 | 0.06250 | 0.09375 |

The exact Friedman test across Z50, Z100 and Z200 yielded statistic 10.333, P = 0.00167181 and Kendall's W = 0.861 (46,656 within-sample rank permutations). This omnibus test concerns differences among concentrations, not comparisons with vehicle. Five samples increased at both concentration steps; M03 decreased from 1.85 to 1.80 between Z100 and Z200.

All pairwise P values are from exact two-sided sign-flip tests of the mean paired difference (64 sign assignments). Holm adjustment is applied separately to the five comparisons in A and three comparisons in B. Confidence intervals are marginal 95% percentile intervals from all 46,656 ordered bootstrap resamples of the six samples.
