# Supplementary material for: Targeted Chemical Monitoring and Integrated Preclinical Evaluation of Zishui Qinggan Yin in 4-NQO-Induced Esophageal Precancer: Histopathological and PI3K/AKT/mTOR-Associated Changes
Source: Life (Basel). 2026 Sep 21;16(9):1574. doi: 10.3390/life16091574 (PMC13608247; doi:10.3390/life16091574)
Supplement: Supplementary file 1 [file life-16-01574-s001.zip › Table S17.docx]

**DARTS Supplementary Results Table S17**

Table S17. Residual signal after Pronase digestion for Cohort B

Residual signal equals 100 times Vehicle/no-Pronase integral. This table documents partial digestion at the tested condition.

| **Sample** | **No Pronase integral** | **Vehicle integral** | **Residual signal (%)** |
| --- | --- | --- | --- |
| M01 | 10000 | 4000 | 40.0 |
| M02 | 12000 | 5040 | 42.0 |
| M03 | 9000 | 3420 | 38.0 |
| M04 | 11000 | 4510 | 41.0 |
| M05 | 10500 | 3990 | 38.0 |
| M06 | 9500 | 4085 | 43.0 |

Mean +/- SD: 40.3 +/- 2.1%; range: 38.0-43.0%; marginal bootstrap 95% CI for the mean: 38.8-41.8%. This does not establish a Pronase dose-response, validate an optimal protease-to-protein ratio, or establish detector linearity.

Mouse identifiers in Tables S15-S17 are Cohort B-specific and are not cross-cohort identifiers.
